# Health literacy and adherence to the pharmacological treatment by people with arterial hypertension

**DOI:** 10.1590/0034-7167-2022-0008

**Published:** 2022-08-08

**Authors:** Iorana Candido da Silva, Maria Rayssa do Nascimento Nogueira, Tahissa Frota Cavalcante, Gilvan Ferreira Felipe, Huana Carolina Cândido Morais, Rafaella Pessoa Moreira, Andressa Suelly Saturnino de Oliveira

**Affiliations:** I Universidade da Integração Internacional da Lusofonia Afro-Brazileira. Redenção, Ceará, Brazil

**Keywords:** Hypertension, Health Literacy, Medication Adherence, Nursing, Chronic Disease, Hipertensión, Alfabetización en Salud, Cumplimiento de la Medicación, Enfermería, Enfermedad Crónica, Hipertensão, Letramento em Saúde, Adesão à, Medicação, Enfermagem, Doença Crônica.

## Abstract

**Objective:**

To analyze the association between health literacy and the adherence to the pharmacological treatment of Brazilians with arterial hypertension.

**Methods:**

Cross-sectional study with 234 participants who responded an on-line sociodemographic and clinical characterization questionnaire, in addition to evaluation of health literacy and of the adherence to their pharmacological treatment. Data were analyzed using descriptive statistics and difference and correlation tests.

**Results:**

People with post-graduation, who were actively working and did not smoke, had better health literacy results. Elders and those who were retired or lived on government subsidies adhered better to the medication treatment. There was a correlation between the numerical dimension (r_s_=0.189; p=0.004) and the global health literacy result (r_2_=0.170; p=0.009) with the adherence.

**Conclusions:**

A better numerical and global understanding of health literacy was associated with better adherence to arterial hypertension medication treatment.

## RESUMO

**Objetivo:**

Analisar a associação entre o letramento em saúde e a adesão ao tratamento farmacológico de brasileiros com hipertensão arterial.

**Métodos:**

Estudo transversal, desenvolvido com 234 participantes, que responderam um questionário on-line de caracterização sociodemográfica, clínica, avaliação do letramento em saúde e da adesão ao tratamento farmacológico.Os dados foram analisados com estatística descritiva e testes de diferença e correlação.

**Resultados:**

Pessoas com pós-graduação, que trabalhavam e não tabagistas obtiveram melhores resultados de letramento em saúde. Idosos e aposentados/pensionistas apresentaram melhor adesão ao tratamento medicamentoso. Houve correlação entre a dimensão numérica (r_s_=0,189; p=0,004) e o resultado global de letramento em saúde (r_s_ = 0,170; p=0,009) com a adesão.

**Conclusões:**

Melhor compreensão numérica e global de letramento em saúde estiveram associados a melhores resultados de adesão ao tratamento medicamentoso da hipertensão arterial.

## RESUMEN

**Objetivo:**

Analizar relación entre alfabetización en salud y adhesión al tratamiento farmacológico de brasileños con hipertensión arterial.

**Métodos:**

Estudio transversal, desarrollado con 234 participantes, que respondieron una encuesta online de caracterización sociodemográfica, clínica, evaluación de la alfabetización en salud y de la adhesión al tratamiento farmacológico. Los datos fueron analizados con estadística descriptiva y testes de diferencia y correlación.

**Resultados:**

Personas con postgrado, que trabajabany no tabaquistas obtuvieron mejores resultados de alfabetización en salud. Ancianos yjubilados/pensionistas presentaron mejor adhesión al tratamiento medicamentoso. Hubo correlación entre la dimensión numérica (r_s_ = 0,189; p = 0,004) yel resultado global de alfabetización en salud (r_s_ = 0,170; p = 0,009) con la adhesión.

**Conclusiones:**

Mejor comprensión numérica y global de alfabetización en salud estuvieron relacionados a mejores resultados de adhesión al tratamiento medicamentoso de la hipertensión arterial.

## INTRODUCTION

The quality of health care and the success of the management of the patient’s clinical condition involve their level of understanding about the disease^(1)^. Considering that, health literacy (HL) is an important factor to be considered so positive results can be achieved. Its definition is the understanding of health recommendations and the continuation of the treatment, involving two fields of knowledge: health and education. Education is related with the literacy process^(2)^; health is associated with the perspective of health promotion and prevention^(3)^.

The continuation of arterial hypertension (AH) therapies is influenced by the level of health literacy (HL), since the ability to understand health orientations is significant to maintain one’s quality of life^(4)^. HL contributes for the control of arterial pressure, the practice of physical activities, and a better understanding about health. Furthermore, it is relevant and significant for education in health, providing positive results in the long term^(5-9)^.

The adherence to treatment, defined by the fulfillment of recommendations from health professionals, is another essential factor to reach good therapeutic results. In a pharmacological treatment, non-adherence takes place when the person stops using medication with no recommendation to do so from trained professionals, or use these medications incorrectly, with delays or interruptions^(10)^.

In Brazilian scientific literature, studies address HL and adherence to pharmacological treatment as separate issues. The evaluation of HL in patients with different clinical conditions, such as diabetes, renal and cardiovascular diseases; literature reviews about instruments that evaluate both constructs; adherence to medication treatment as related to self-care; and adherence in elders^(4,11-16)^. In international literature, researches portrait the effect of HL and the efficacy of self-care in quality of life; the association between HL and the adherence to medication between Hispanic people with AH^(17-18)^.

Researches that analyze the association between HL and adherence to AH medication treatment are few. When they take place, they generally include participants from the same setting, such as the same city/state, which are under the same local health agendas. The intention, in this study, was to analyze this association in the country as a whole, considering people from all Brazilian regions.

The guiding question for this research was: “Is there an association between the HL levels and the adherence of Brazilians with AH to pharmacological treatment?” The nurse, health professional who often has the first contact with the user and carries out several follow up consultations after diagnosis, must be attentive to these aspects so the care provided in health units is adequate. Considering aspects that can influence the adherence to treatment increases the autonomy of the user and improves their engagement, since they understand what is being proposed to them.

The development of studies with this topic allows one to know the level of understanding of the patients in regard to the information provided about their health-disease process, so health actions can be planned and developed. Moreover, HL knowledge can aid in the professional practice in regard to health education using active methodologies, which attend to the needs of individuals in a social, cultural, and educational context^(19)^.

## OBJECTIVE

To analyze the association between health literacy and the adherence to the pharmacological treatment of Brazilians with arterial hypertension.

## METHODS

### Ethical aspects

This research was approved by the Research Ethics Committee at the Universidade Internacional da Lusofonia Afro-Brasileira (UNILAB). It respected and adhered to all ethical precepts in Resolution No. 466/2012 and in the Circular Letter No. 2/2021 from the National Council of Research Ethics.

### Design, period, and place of study

Cross-sectional study developed according with the precepts prescribed by the checklist STROBE. Data collection took place in February 2021. Its setting was the Internet. The promotion of the research and recruiting of participants was carried out in the social networks Instagram, WhatsApp, and Facebook.

### Population or sample; criteria for inclusion and exclusion.

The study counted on the participation of 234 people. The sample was defined using a calculation for an infinite population, using a formula for descriptive studies. The proportion used was 32.3% (prevalence of AH, in Brazil^(20)^, with an error of 6% and a confidence level of 95%). The sample was non-probabilistic (intentional). The inclusion criteria presented for the users of the social networks were: being 18 years old or older, Brazilian living in Brazil, having a diagnosis of AH, and using at least one antihypertensive medication.

### Study protocol

Recruitment was carried out via dissemination in social networks, with virtual invitations that clearly presented the eligibility criteria. The interest in participating in the research was manifested by clicking in the link available, which showed general information about the study and the Free and Informed Consent Form (FICF) before the data collection instrument.

The electronic questionnaire, produced on Google Forms, was divided in four parts: 1) sociodemographic characterization; 2) clinical characterization (related with the disease and the lifestyle); 3) HL evaluation - Test of Functional Health Literacy in Adults - Short version (S-TOFHLA, Portuguese language version); and 4) evaluation of the adherence to the pharmacological treatment - Morisky-Green Test (MGT, Portuguese language version).

The S-TOFHLA is formed by dimensions of reading comprehension (36 questions, 2 points for each correct item, to a maximum of 72 points) and numeracy (4 question, 7 points each correct item, to maximum of 28 points). According with the performance in the test, the HL of the individual can be classified as inadequate (0 to 53 points), marginal (54 to 66 points), or adequate (67 to 100 points )^(21)^.

The MGT is formed by four questions with two possible responses. The alternative “yes” is worth 0 (zero) points, indicating non-adherence; the alternative “no” is worth 1 (one) point, indicating adherence. The results can be classified as: high adherence (4 points), medium adherence (2 or 3 points), and low adherence (0 or 1 point)^(22)^.

### Analysis of results and statistics

An on-line spreadsheet was created automatically via Google, associated with the collection instrument. The statistical package IBM SPSS Statistics, version 22 (license code 9DNCAF2O3QVDV7FBIO696OO6GWLNXZPPRYTPWF2PPX7C8T6Y24LMVV2ET7DOLX5CXL5YDLL79UPGEFCPDXP5Q8O5E) was used for a descriptive and inferential analysis, after data from the original on-line spreadsheet was imported.

The absolute and relative frequencies of variables regarding parts 1 and 2 of the data collection instruments were calculated. The mean and standard deviation of parts 3 and 4 of the instrument were also calculated. The results of the two S-TOFHLA dimensions (independent and added up) and of the MGT were submitted to normality and distribution analysis using the Kolmogorov-Smirnov test.

After the heterogeneity of the distribution of quantitative variables was verified, the non-parametric tests of Mann-Whitney and Kruskal-Wallis were used to verify the existence of any association between sociodemographic and clinical characterization variables and the results of S-TOFHLA and MGT. The association between the results of S-TOFHLA and MGT were analyzed using Spearman’s correlation coefficient. *P*-values below 0.05 were considered to be significant^(23)^.

## RESULTS

The majority of the 234 participants were women (n = 206; 88.0%), with a mean age of 50 + 10.6 years old, high school education (n = 102; 43.6%), a job that allowed them a monthly income (n = 95; 40.6%), and lived in the Southeast of Brazil (n = 109; 46.6%). The sample included the same proportion of people with and without partners(a) (Table 1).

**Table 1:** Health literacy and adherence to the pharmacological treatment according with participant sociodemographic characteristics of the participants (n=234), Brazil, 2021

Variables	n	%	S-TOFHLA dimensions		S-TOFHLA	*p* value	MGT	*p* value
			Reading	Numeracy	Mean + SD		Mean + SD	
			Mean + SD	Mean + SD				
Sex								
Female	206	88.0	71.2 ± 3.3	23.4 ± 6.4	94.6 ± 8.0	0.644^*^	2.5 ± 1.4	0.638^*^
Male	28	12.0	70.2 ± 4.6	25.2 ± 3.4	95.5 ± 5.0		2.5± 1.3	
Age group								
Adults	190	81.2	71.1 ± 3.5	23.7 ± 5.9	94.8 ± 7.4	0.577^*^	2.4 ± 1.4	0.008^*^
Elders	44	18.8	70.8 ± 3.4	23.2 ± 7.2	94.5 ± 8.7		3.0 ± 1.2	
Years of formal education								
Elementary school	20	8.5	70.2 ± 7.1	22.0 ± 6.9	92.2 ± 11.1	0.012^‡^	2.2 ± 1.2	0.092^‡^
High School	102	43.6	70.8 ± 3.5	22.4 ± 6.8	93.3 ± 8.6		2.4 ± 1.4	
Higher education	57	24.4	71.6 ± 0.8	24.4 ± 5.6	96.1 ± 5.5		2.9 ± 1.1	
Post-graduation	55	23.5	71.2 ± 3.3	25.5 ± 4.3	96.8 ± 5.3		2.5 ± 1.3	
Marital status								
Has a partner	117	50.0	71.1 ± 3.4	23.8 ±5.9	94.9 ± 7.6	0.463^*^	2.6 ± 1.4	0.691^*^
Does not have a partner	117	50.0	71.0 ± 3.7	23.3 ± 6.3	94.4 ± 7.8		2.5 ± 1.3	
Region of residence								
Midwest	16	6.8	71.7 ± 0.6	24. 9 ± 4.4	96.6 ± 4.6	0.727^‡^	2.2 ± 1.4	0.087^‡^
Northeast	66	28.2	69.9 ± 6.0	23.2 ± 6.7	93.1 ± 10.0		2.2 ± 1.5	
North	9	3.8	71.7 ± 0.6	22.5 ± 6.8	94.3 ± 6.8		2.5 ± 1.0	
Southeast	109	46.6	71.4 ± 1.9	24.0 ± 5.7	95.5 ± 6.1		2.7 + 1.3	
South	34	14.5	71.7 ± 0.7	22.6 ± 6.9	94.3 ± 7.1		2.9 ± 1.4	
Paid work								
Yes	95	40.6	71.4 ± 2.6	24.6 ± 5.3	96.0 ± 5.8	0.034^‡^	2.4 ± 1.3	0.023^‡^
No	89	38.0	70.8 ± 4.4	22.3 ± 6.7	93.1 ± 8.8		2.3 ± 1.4	
Retired/receives subsidies	50	21.4	70.8 ± 3.4	24.0 ± 6.1	94.9 ± 8.3		3.0 ± 1.1	

* *Statistically significant according with Mann-Whitney’s (p<0.05);*

‡ *statistically significant according with Kruskal-Wallis’s; S-TOFHLA - Test of Functional Health Literacy in Adults - Short version; MGT - Morisky-Green Test; SD - standard deviation.*

The general mean of the evaluation instrument of HL (S-TOFHLA) was 94.7 ± 7.7, indicating an adequate level. The respondents had the same performance in reading comprehension (71.2 ± 3.5) and numeracy (23.6 ± 6.1). Based on the results of the sum totals, 98.7% were classified as presenting adequate HL, while 1.3% presented marginal HL.

The HL was classified as adequate regardless of sex, age group, marital status, or region of residence. Although the classification was the same, higher S-TOFHLA scores were found among people with post-graduation (96. 8± 5.3; p=0.012) and those who had a job (96.0 ± 5.8; p=0.034), suggesting that people with those sociodemographic characteristics had better HL results (Table 1).

The median of the summed results of the sample, found using the MGT, was 3 (IIQ = 2), which is equivalent to the medium adherence to the pharmacological treatment of AH. Participants with the three degrees of adherence of the instrument were found: 24.4% (n=55) had low adherence; 40.2% (n=93), a medium one; and 35.5% (n=83), a high adherence.

Analyzing these elements in isolation, those with the highest frequency of adherence were the answers that indicated that medication was not interrupted even when the person felt better (79.9%) or worse (64.5%).

The mean adherence level was not related with sex, educational level, marital status, and region of residence. Even with this degree, higher scores were found among elder (3.0 ± 1.2; p = 0.008) and those who are retired or live on government subsidies (3.0 ± 1.1; p = 0.023),

Most participants were obese (n = 127; 54.3%), did not smoke (n = 209; 89.3%), did not drink alcohol (n = 118; 50.4%) or did so infrequently (n = 110; 47.0%) and was sedentary (n = 154; 65.8%). They reported a long time living with AH, since diagnosis, which varied from less than one year to more than ten years, the latter being the most frequent result (n=87; 37.2%) (Table 3).

**Table 3:** Correlation between health literacy and participant adherence to pharmacological treatment (n=234), Brazil, 2021

S-TOFHLA	MGT	
	r_s_	*p* value^*^
Reading comprehension	0.007	0.914
Numeracy	0.189	0.004
Total	0.170	0.009

* *Statistically significant according with Spearman’s (p<0.05); S-TOFHLA - Test of Functional Health Literacy in Adults - Short version; MGT - Morisky-Green Test.*

Although most participants did not have a diagnosis of other diseases than AH (n=134; 57.3%), among those who did, diabetes mellitus was the most common. Other answers that appeared include renal failure, arterial coronary disease, cerebrovascular accident, and myocardium infarction. Most participants used up to three medications daily (n=141; 60.3%), including the medication to treat AH.

The HL was classified as adequate regardless of body mass index (BMI), alcohol consumption, practice of physical activities, time since AH diagnosis, diagnosis of comorbidities, and number of medications used daily. Participants who did not smoke had a higher S-TOFHLA mean (95.0 ± 7.7; p = 0.022).

Regarding the MGT, none of the clinical characteristics analyzed in Table 2 showed a significant association with the results of this variable. In summation, in this research, participants showed a medium adherence (indicated by the 2-point means), regardless of the clinical characteristics investigated.

**Table 2:** Health literacy and adherence to the pharmacological treatment according with participant clinical characteristics of the participants (n=234), Brazil, 2021

Variables	n	%	S-TOFHLA dimensions		S-TOFHLA	*p* value	MGT	*p* value
			Reading	Numeracy	Mean + SD		Mean + SD	
			Mean + SD	Mean + SD				
BMI								
Low weight	1	0.4	72.0	28.0	100.0	0.474^‡^	4.0	0.134^‡^
Normal weight	27	11.5	71.2 ± 3.0	24.6 ± 5.2	95.8 ± 6.3		2.8 ± 1.3	
Overweight	77	32.9	70.2 ± 5.4	23.1 ± 6.5	93.4 ± 9.6		2.7 ± 1.4	
Obesity	127	54.3	71.5 ± 1.7	23.7 ± 6.1	95.2 ± 6.6		2.3 ± 1.3	
Did not answer	2	0.9	72.0	21.0	93.0		3.5 ± 0.7	
Smoker								
Yes	25	10.7	71.7 ± 0.8	20.7 ± 7.1	92.4 ± 7.0	0.022^*^	2.4 ± 1.6	0.706^*^
No	209	89.3	71.0 ± 3.7	23.9 ± 5.9	95.0 ± 7.7		2.6 ± 1.3	
Drinks alcohol								
Yes, occasionally	110	47.0	71.4 ± 2.6	24.6 ± 4.7	96.0 ± 5.6	0.152^‡^	2.4 ± 1.4	0.077^‡^
Yes, frequently	6	2.6	69.3 ± 4.6	23.3 ± 5.7	92.6 ± 9.8		1.6 ± 1.0	
No	118	50.4	70.9 ± 4.2	22.6 ± 7.1	93.5 ± 9.0		2.7 ± 1.3	
Practices physical exercise								
Yes	80	34.2	71.3 ± 2.2	23.6 ± 6.0	94.9 ± 6.5	0.871^*^	2.6 ± 1.3	0.987^*^
No	154	65.8	70.9 ± 4.0	23.6 ± 6.2	94.6 ± 8.2		2.5 ± 1.4	
Time since AH diagnosis								
Less than 1 year	25	10.7	71.5 ± 0.8	23.2 ± 5.6	94.7 ± 5.8	0.573^‡^	2.6 ± 1.5	0.215^‡^
1 to 5 years	56	23.9	71.1 ± 3.2	23.7 ± 6.8	94.8 ± 8.7		2.4 ± 1.3	
5 to 10 yeras	66	28.2	71.5 ± 1.5	23.0 ± 6.4	94.5 ± 6.6		2.3 ± 1.3	
More than 10 years	87	37.2	71.5 ± 0.8	23.2 ± 5.6	94.7 ± 5.8		2.6 ± 1.5	
Diagnosed comorbidities								
Yes	100	42.7	70.6 ± 4.5	22.8 ± 7.0	93.5 ± 9.4	0.166^*^	2.5 ± 1.3	0.517^*^
No	134	57.3	71.4 ± 2.6	24.2 ± 5.3	95.6 ± 6.0		2.6 ± 1.3	
Number of medications								
Até 3	141	60.3	71.1 ± 3.8	23.9 ± 5.8	95.0 ± 7.5	0.230^*^	2.6 ± 1.4	0.717^*^
4 or more	93	39.7	71.0 ± 3.1	23.1 ± 6.6	94.2 ± 7.9		2.5 ± 1.3	

* *Statistically significant according with Mann-Whitney’s (p<0.05);*

‡ *statistically significant according with Kruskal-Wallis’s; S-TOFHLA - Test of Functional Health Literacy in Adults - Short version; MGT - Morisky-Green Test; SD - standard deviation; BMI - body mass index; AH - arterial hypertension.*

There was no correlation between the best MGT results and the best S-TOFHLA reading comprehension tests (Table 3). However, the relationship between MGT and numeracy participants was positive and significant, and the same was true for the overall result of the HL instrument (r_s_ = 0.170; p = 0.009). Therefore, although almost all participants had an adequate HL, good numeracy abilities were related to better results in regard to adherence to antihypertensive pharmacological treatment. This result is the same when the overall results of the HL evaluation instrument are analyzed.

## DISCUSSION

In this research, the HL of the participants was predominantly satisfactory. This result is unlike that of most studies published in Brazil, which generally indicate that people with AH have low HL^(1,19,21,24-25)^. The on-line application of the S-TOFHLA, which allowed for patients to use as much time as they want to read and reread HL evaluation items, may have influenced results. There is also a different educational profile between participants in researches carried out on-line as opposed to in-person. Most Internet users, especially those who agree participating in researches, are characterized by high educational levels. As an example, most participants finished, at least, high school, which would correspond to approximately 15 years of schooling.

Still, the performance of participants in regard to numeracy had a lower result than in reading comprehension. This shows how necessary it is for nurses (and other professionals responsible for the follow up of these individuals) to remind patients, in consultations, about the time to take the medication, the correct dosages, explaining the results of any exams required and providing information about the scheduling of attention^(1,8)^.

Not only the numeracy result is associated with the schooling of patients, it also seems to reflect how the nurse has been dealing with quantitative information in health during consultations. Although understanding this is important for the user to assume the main role in their own care, this is rarely treated as an important aspect of the process due to several factors, such as the little time to carry out consultations and the non-utilization of instruments to guide the user that can allow one to identify specific needs each time they are attended.

It is also well-known that the consultation is not always focused on the user, with vertical actions of care in which the professional barely listens to the user. This posture does not allow for the identification of issues such as patients who do not take medication at the right time due to the fact they do not understanding the information provided^(16)^.

The association of HL and paid work is, inevitably, reflective of educational level. The literacy of people influences their HL and is an important predictor of income, employability, and educational level. Therefore, aspects related with socioeconomic, cultural, environmental, and biological origins are social determinants of health^(26)^.

Participants who did not smoke also presented a better HL. Still considering schooling, a higher educational level generally results in deeper knowledge about health. This has been shown to reflect on conscious decision making about healthier life habits^(27)^. For people with AH, these results are also important when cardiovascular risk is considered. Literature shows that the educational level has a clear role in the evolution of AH, with strong associations between lower educational levels and global cardiovascular risk. In this regard, it is clear that education should be considered one of the strategies to care for AH and prevent other cardiovascular diseases^(28)^.

Regarding adherence to medication treatment, most presented a moderate adherence. On the other hand, there was a high prevalence of unhealthy clinical characteristics, such as inactivity, drinking, and obesity. This may be an indicative that, on the long term, it will be necessary to increase the number of medications prescribed to reach the adequate pressure levels, although it is important to highlight that less than half respondents were in polypharmacy.

Participants who were elders and retired/subsidy recipients showed a better adherence to the medication treatment when compared to other respondents, a result that is opposed to the findings of other researches. Literature shows that age and adherence are inversely proportional: elders tend to present physical, mental, and cognitive conditions that imply difficulties in comprehending the therapeutic treatment and adhering to it^(29-30)^.

However, the result may be justified by the fact that the AH has been diagnosed for a long period (more than 10 years). In general, the higher the time of diagnosis, the higher the knowledge about the disease and the treatment^(30)^. Moreover, the number of medications and comorbidities is directly proportional to adherence^(30-31)^. Therefore, the small amount of medications ingested by most patients (up to three) and the fact that more than half participants had no associated disease may also justify this result.

Another issue the nurse must consider in regard to elder adherence to medication treatment is their social support. This is represented by partners, children, and other relatives, when there are any. It needs to be considered in the care plan. The results related with adherence in this population group, in this research, may be related to this aspect.

As a result, it is essential for all those involved in interdisciplinary care to be aware of the characteristics of the users, and those that can interfere in HL and treatment adherence, especially when it comes to chronic disease. In this context, establishing a connection between users and professionals is essential to reach good results. Communication is key in this process. The health worker must be aware of verbal, non-verbal, and written elements used during consultation, to be sure that what is being said is being understood, and the therapy prescribed is being followed^(30,32)^.

In scientific publications about the adherence to AH treatment, the adherence rates of individuals with higher HL levels are, on average, 14% higher than those of patients with low HL. Still, it is important to have in mind that, the fact that the patient has knowledge and understands information about their health-disease process is not sufficient in itself to imply on the adherence to pharmacological treatment or lack thereof, since it involves more complex aspects that must be analyzed, such as psychological, behavioral, clinical, and socioeconomic factors^(8,33-34)^.

The fact that a high performance in the S-TOFHLA numeracy dimension was correlated with the highest results of adherence to AH pharmacological treatment reiterate the need for the nurse to consider whether the patient is understanding health guidance that involves numerical reasoning, searching strategies to fixate the information in the users’ memory.

### Study limitations

A limitation of this research was the procedural bias. Data collection through the Internet allowed respondents to read the HL evaluation instrument several times, for a period of time that cannot be standardized (longer than if the application was in person, for instance), which may have led to better HL results. The virtual setting may also have limited the diversity of participants due to the sociodemographic characteristics of the users of digital media. Thus, the profile of the public presented in this study may be different than that found in health services.

### Contributions to the field of nursing

Getting to know the HL of patients with AH is essential to provide care directed at the real needs of the patients. As nurses evaluate HL, they can, in consultations to people with this morbidity, understand how necessary specific guidance is, such as scheduling, dosage, and dates for the attention to those who have some difficulty understanding numerical information in health. Since these are important information for the adherence to the antihypertensive treatment, they must be addressed more emphatically in the attention.

## CONCLUSIONS

The HL level of research participants was classified as adequate, and they presented a mean adherence to pharmacological treatment. The study concluded that a higher numeracy comprehension and a better global HL result were associated with better adherence to AH medication treatment. Those with a higher educational level, who had a paid work, had a better performance in the HL. Participants who did not smoke presented a higher HL mean. The adherence was higher in elders and retired people or subsidy recipients.

Knowing and understanding these findings allows one to adapt the care provided by the nurses and other professionals in the public health services, since the quality of health care and its adequate management are influenced by the HL level of the individual.

## SUPPLEMENTARY MATERIAL

https://doi.org/10.48331/scielodata.0QVJYO.

0034-7167-reben-75-06-e20220008-sup01
