# Supplementary material for: Health literacy and adherence to the pharmacological treatment by people with arterial hypertension
Source: Rev Bras Enferm. 2022 Aug 8;75(6):e20220008. doi: 10.1590/0034-7167-2022-0008 (PMC9749777; doi:10.1590/0034-7167-2022-0008)
Supplement: 0034-7167-reben-75-06-e20220008-sup01 [file 0034-7167-reben-75-06-e20220008-sup01.pdf]

Carimbo de data/hora Voc� j� viu esta imagem em alguma outra pesquisa? O(A) Senhor(a) aceita participar desta pesquisa? Quais s� o as duas primeiras letras do seu nome? (por exemplo, se voc� se chama Jos� , digite JO) Qual foi o ano em que voc� nasceu? Voc� tem 18 anos ou mais? Qual � o seu grau de instru� � o (escolaridade)? E m qual regi� o do Brasil voc� mora hoje? Qual � o seu estado civil? Contando com voc� , quantas pessoas mora m na sua casa? Escrever apenas o n� mero. Se morar sozinho(a), escrever 1. Qual � o seu sexo? Voc� tem algum trabalho, hoje, que lhe permite receber um sal� rio? Algum m� dico j� lhe diagnosticou com hipertens� o arterial ? Voc� toma algum medicamento (rem� dio) prescrito por um profissional de sa� de para tratamento da hipertens � o arterial? Qual � o seu peso em quilos (aproximadamente)? Escreva apenas os n� meros (exemplo: 95). Qual � a sua altura (aproximadamente)? Escreva apenas os n� meros (exemplo: se voc� tem 1,59, escreva 159). Voc� fuma? Voc� toma bebida alco� lica? Voc� pratica exerc� cio f� sico (pelo menos 3 vezes por semana)? H� qua nto tempo voc� foi diagnosticado com hipertens� o arterial? Voc� tem alguma destas doen� as? Voc� pode mar car mais de uma, se for o caso. Incluindo o medicamento para tratamento da hipertens� o, quantos medicamentos, p ara qualquer finalidade, voc� toma num dia? Escreva apenas o n� mero. Seu m� dico encaminhou voc� para tirar um Raio X de __________. Quando vier para o __________ voc� deve estar com o est� mago __________. O ex ame de Raio X vai __________ de 1 a 3 __________. � V� SPERA DO DIA DO RAIO X: No jantar, coma somen te um peda� o __________ de fruta, torradas e geleia, com __________ ou ch� . � V� SPERA DO DIA DO RAI O X: Ap� s __________, voc� n� o deve __________ nem beber __________ at� __________ o Raio X. NO DI A DO RAIO X: N� o tome __________. NO DIA DO RAIO X: N� o _________, nem mesmo _________ . NO D IA DO RAIO X: Se voc� tiver alguma _________, ligue para _________ de Raio X no telefone (19) 2222-2821. E u concordo em dar informa� � es corretas para _________ receber atendimento adequado neste Hospital. Eu _____ ____ que as informa� � es que eu _________ ao m� dico ser� o muito _________ para permitir o correto ______ ___ . Eu _________ que devo relatar para o m� dico qualquer _________ nas minhas condi� � es dentro de _____ ____ (10) dias, a partir do momento em que me tornar _________ da altera� � o. Eu entendo _________ se EU N � O me _________ ao tratamento, tenho _________ de _________ uma nova consulta _________ para o hospital. S e voc� _________ de ajuda para entender estas _________, voc� dever� _________ uma enfermeira ou funcion � ria do _________ Social para _________ todas as suas _________. Se voc� tomasse a primeira c� psula � s 7 horas da manh� , a que horas voc� deveria tomar a pr� xima? Escreva o hor� rio. Se essa fosse sua taxa de glicem ia hoje, estaria normal? Se este fosse seu cart� o, quando seria sua pr� xima consulta? Escreva a data. Se voc� foss e almo� ar � s 12 horas e quisesse tomar a medica� � o antes do almo� o, a que horas voc� deveria tom� -la? Es creva o hor� rio. Voc� � s vezes tem problemas em se lembrar de tomar a sua medica� � o? Voc� � s vezes se descuida de tomar seu medicamento? Quando est� se sentindo melhor, voc� � s vezes p� ra de tomar seu medica mento? � s vezes, se voc� se sentir pior ao tomar a medica� � o, voc� p� ra de tom� -la?

1/6/2021 18:54:52 N� o SIM, aceito participar. Ama 21/01/1990 Sim Ensino m� dio completo (colegial). Centro-O este Casado(a) / Vive junto 2 Feminino Sim Sim Sim 79 165 N� o Sim, � s vezes. N� o Menos de 1 ano. Outra que n� o est� entre as op� � es de resposta 2 est� mago raio x, vazio durar, horas pequeno, caf� a meia-noite, come r, nada, fazer caf� da manh� beba, � gua pergunta, o departamento poder compreendendo, transmitir, importantes, di agn� stico entendi, altera� � o, dez, ciente que, adaptar, direito, solicitar, telefonando precisar, instru� � es, procur ar, Servi� o, esclarecer, d� vidas 13:00 N� o 07/01/2021 11:00 N� o N� o N� o N� o

1/6/2021 19:36:41 N� o SIM, aceito participar. LI 01/09/1978 Sim Ensino m� dio completo (colegial). Nordeste Se parado(a) / Divorciado(a) 4 Feminino N� o Sim Sim 70 160 N� o N� o. N� o Mais de 10 anos. Outra que n� o est � entre as op� � es de resposta 1 est� mago raio x, vazio durar, horas pequeno, caf� a meia-noite, comer, nada, f azer caf� da manh� beba, � gua pergunta, o departamento poder compreendo, transmitir, importantes, diagn� stic o entendi, altera� � o, dez, ciente que, adaptar, direito, solicitar, telefonando precisar, instru� � es, procurar, Servi � o, esclarecer, d� vidas 13:00 N� o 07/01/2021 11:00 Sim N� o N� o N� o

1/6/2021 21:16:30 N� o SIM, aceito participar. MA 24/09/1969 Sim P� s-Gradua� � o (especializa� � o, mestrad o e/ou doutorado). Nordeste Casado(a) / Vive junto 4 Feminino Sim Sim Sim 79 149 N� o N� o. N� o Menos de 1 ano. Outra que n� o est� entre as op� � es de resposta 4 est� mago raio x, vazio durar, horas pequeno, caf� a me ia-noite, comer, nada, fazer caf� da manh� beba, � gua pergunta, o departamento poder compreendo, transmitir, i mportantes, diagn� stico entendi, altera� � o, dez, ciente que, adaptar, direito, solicitar, telefonando precisar, instru � � es, procurar, Servi� o, esclarecer, d� vidas 01:00 N� o 07/01/2021 15:00 N� o N� o N� o N� o

1/6/2021 21:37:20 N� o SIM, aceito participar. LU 03/01/1989 Sim P� s-Gradua� � o (especializa� � o, mestrad o e/ou doutorado). Nordeste Solteiro(a) 4 Masculino Sim Sim Sim 87 174 N� o Sim, � s vezes. Sim 5 a 10 anos. Ou tra que n� o est� entre as op� � es de resposta 2 est� mago raio x, vazio durar, horas pequeno, caf� a meia-noite , comer, nada, fazer caf� da manh� beba, � gua pergunta, o departamento poder compreendo, transmitir, importan tes, diagn� stico entendi, altera� � o, dez, ciente que, adaptar, direito, solicitar, telefonando precisar, instru� � es,

procurar, Servi� o, esclarecer, d� vidas 13:00 N� o 01/07/2021 11:00 Sim Sim Sim N� o
1/6/2021 21:54:16 N� o SIM, aceito participar. Ve 20/03/2021 Sim P� s-Gradua� � o (especializa� � o, mestrado e/ou doutorado). Nordeste Casado(a) / Vive junto 5 Feminino Sim Sim Sim 102 175 N� o N� o. N� o 5 a 10 anos. Outra que n� o est� entre as op� � es de resposta 3 diabetes raio x, vazio durar, horas pequeno, caf� a meia-noit e, comer, nada, fazer caf� da manh� beba, � gua pergunta, o departamento poder compreendo, transmitir, importa ntes, diagn� stico entendi, altera� � o, dez, ciente que, adaptar, direito, solicitar, telefonando precisar, instru� � es, procurar, Servi� o, esclarecer, d� vidas 12 N� o 07/01/2021 11 N� o Sim N� o N� o
1/6/2021 21:55:48 N� o SIM, aceito participar. ID 21/01/1971 Sim P� s-Gradua� � o (especializa� � o, mestrado e/ou doutorado). Nordeste Casado(a) / Vive junto 6 Feminino N� o Sim Sim 75 155 N� o Sim, � s vezes. Sim 5 a 10 anos. Outra que n� o est� entre as op� � es de resposta 2 est� mago raio x, vazio durar, horas pequeno, caf� a meia-noite, comer, nada, fazer caf� da manh� beba, � gua pergunta, o departamento poder compreendo, transmi tir, importantes, diagn� stico entendi, altera� � o, dez, ciente que, adaptar, direito, solicitar, telefonando precisar, in stru� � es, procurar, Servi� o, esclarecer, d� vidas 13h N� o 07/01/2021 11h Sim Sim Sim Sim
1/6/2021 21:57:14 N� o SIM, aceito participar. AL 03/10/1961 Sim Ensino fundamental completo. Nordeste Casad o(a) / Vive junto 5 Masculino N� o Sim Sim 50 155 Sim N� o. Sim 1 a 5 anos. Outra que n� o est� entre as op� � es de resposta 2 est� mago raio x, vazio durar, horas pequeno, caf� a meia-noite, comer, nada, fazer caf� da ma nh� beba, � gua pergunta, o departamento poder compreendo, transmitir, importantes, diagn� stico entendi, altera � � o, dez, ciente que, adaptar, direito, solicitar, telefonando precisar, instru� � es, procurar, Servi� o, esclarecer, d� vidas 12 N� o 07/01/2021 11 Sim Sim Sim Sim
1/6/2021 23:13:07 N� o SIM, aceito participar. RA 23/06/1994 Sim Ensino superior completo (faculdade/universida de). Nordeste Solteiro(a) 1 Masculino N� o Sim Sim 65 168 N� o N� o. N� o Mais de 10 anos. N� o, n� o tenho nenhuma dessas doen� as 0 pontos raio x, vazio durar, horas pequeno, caf� a meia-noite, comer, nada, fazer caf� da manh� beba, � gua pergunta, o departamento poder compreendo, transmitir, importantes, diagn� stico entendi, altera� � o, dez, ciente que, adaptar, direito, solicitar, telefonando precisar, instru� � es, procurar, Servi� o, esclar ecer, d� vidas 13h N� o 07/01/2021 11 da manh� N� o N� o N� o N� o
1/6/2021 23:21:42 N� o SIM, aceito participar. FE 24/11/1989 Sim P� s-Gradua� � o (especializa� � o, mestrado e/ou doutorado). Nordeste Solteiro(a) 5 Feminino N� o Sim Sim 63 153 N� o Sim, � s vezes. Sim 1 a 5 anos. N� o, n� o tenho nenhuma dessas doen� as 3 est� mago raio x, vazio durar, horas pequeno, caf� a meia-noite, comer, nada, fazer caf� da manh� beba, � gua pergunta, o departamento poder compreendo, transmitir, importantes, diag n� stico entendi, altera� � o, dez, ciente que, adaptar, direito, solicitar, telefonando precisar, instru� � es, procurar , Servi� o, esclarecer, d� vidas 13 N� o 07/01/2021 11:00 Sim Sim Sim Sim
1/6/2021 23:22:24 N� o SIM, aceito participar. LU 03/01/1989 Sim P� s-Gradua� � o (especializa� � o, mestrad o e/ou doutorado). Nordeste Solteiro(a) 4 Masculino Sim Sim Sim 87 174 N� o Sim, � s vezes. Sim 5 a 10 anos. Ou tra que n� o est� entre as op� � es de resposta 2 est� mago raio x, vazio durar, horas pequeno, caf� a meia-noite , comer, nada, fazer caf� da manh� beba, � gua pergunta, o departamento poder compreendo, transmitir, importan tes, diagn� stico entendi, altera� � o, dez, ciente que, adaptar, direito, solicitar, telefonando precisar, instru� � es, procurar, Servi� o, esclarecer, d� vidas 13:00 N� o 01/07/2021 11:00 Sim Sim Sim N� o
1/7/2021 4:17:37 N� o SIM, aceito participar. ER 25/04/2021 Sim Ensino m� dio completo (colegial). Nordeste Ca sado(a) / Vive junto 04 Feminino Sim Sim Sim 58 150 N� o Sim, � s vezes. Sim Menos de 1 ano. N� o, n� o tenh o nenhuma dessas doen� as 02 est� mago raio x, vazio durar, horas pequeno, caf� a meia-noite, comer, nada, fazer caf� da manh� beba, � gua pergunta, o departamento poder compreendo, transmitir, importantes, diagn� stico en tendi, altera� � o, dez, ciente que, adaptar, direito, solicitar, telefonando precisar, instru� � es, procurar, Servi� o, esclarecer, d� vidas 1hora N� o 07/01/2021 11h Sim Sim Sim Sim
1/7/2021 7:34:24 N� o SIM, aceito participar. WI 18/06/1997 Sim Ensino superior completo (faculdade/universidad e). Nordeste Casado(a) / Vive junto 2 Feminino Sim Sim Sim 63 162 N� o N� o. Sim Menos de 1 ano. Outra que n � o est� entre as op� � es de resposta 8 est� mago raio x, vazio durar, horas pequeno, caf� a meia-noite, comer, nada, fazer caf� da manh� beba, � gua pergunta, o departamento poder compreendo, transmitir, importantes, diag n� stico entendi, altera� � o, dez, ciente que, adaptar, direito, solicitar, telefonando precisar, instru� � es, procurar , Servi� o, esclarecer, d� vidas 13 N� o 07/01/2021 11 Sim Sim N� o Sim
1/7/2021 18:41:37 N� o SIM, aceito participar. MA 10/06/1975 Sim Ensino m� dio completo (colegial). Nordeste Casado(a) / Vive junto 4 Feminino N� o Sim Sim 110 175 N� o Sim, � s vezes. N� o 5 a 10 anos. N� o, n� o ten ho nenhuma dessas doen� as 0 est� mago raio x, vazio durar, horas pequeno, caf� a meia-noite, comer, nada, fazer caf� da manh� beba, � gua pergunta, o departamento poder compreendo, transmitir, importantes, diagn� stico en tendi, altera� � o, dez, ciente que, adaptar, direito, solicitar, telefonando precisar, instru� � es, procurar, Servi� o, esclarecer, d� vidas 13 N� o 14/01/2021 11 N� o N� o N� o N� o

1/7/2021 20:36:49 N� o SIM, aceito participar. Ca 08/04/1982 Sim Ensino m� dio completo (colegial). Nordeste Casado(a) / Vive junto 4 Feminino N� o Sim Sim 83 160 N� o Sim, � s vezes. N� o 1 a 5 anos. Outra que n� o est� entre as op� � es de resposta 1 est� mago raio x, vazio durar, horas pequeno, caf� a meia-noite, comer, nada, fazer caf� da manh� beba, � gua pergunta, o departamento poder compreendendo, transmitir, importantes, diagn� stico entendi, altera� � o, dez, ciente que, adaptar, direito, solicitar, telefonando precisar, instru� � es, procurar, Servi� o, esclarecer, d� vidas 13 N� o 14/01/2021 11 Sim Sim N� o N� o

1/7/2021 21:03:21 N� o SIM, aceito participar. AR 17/01/1966 Sim Ensino fundamental completo. Nordeste Casado(a) / Vive junto 3 Feminino Sim Sim Sim 76 165 N� o N� o. N� o 1 a 5 anos. Outra que n� o est� entre as op� � es de resposta, N� o, n� o tenho nenhuma dessas doen� as 3 est� mago raio x, vazio durar, horas pequeno, caf� a meia-noite, comer, nada, fazer caf� da manh� beba, � gua pergunta, o departamento poder compreendendo, transmitir, importantes, diagn� stico entendi, altera� � o, dez, ciente que, adaptar, direito, solicitar, telefonando precisar, instru� � es, procurar, Servi� o, esclarecer, d� vidas 13 horas N� o 14/01/2021 11h N� o N� o N� o Sim

1/7/2021 23:55:54 N� o SIM, aceito participar. Ka 06/12/1982 Sim P� s-Gradua� � o (especializa� � o, mestrado e/ou doutorado). Nordeste Separado(a) / Divorciado(a) 3 Feminino Sim Sim Sim 79 157 N� o N� o. Sim 1 a 5 anos. N� o, n� o tenho nenhuma dessas doen� as 1 est� mago raio x, vazio durar, horas pequeno, caf� a meia-noite, comer, nada, fazer caf� da manh� beba, � gua pergunta, o departamento poder compreendendo, transmitir, importantes, diagn� stico entendi, altera� � o, dez, ciente que, adaptar, direito, solicitar, telefonando precisar, instru� � es, procurar, Servi� o, esclarecer, d� vidas 13 N� o 14/01/2021 11 Sim Sim Sim Sim

1/8/2021 23:10:47 N� o SIM, aceito participar. Th 29/12/1988 Sim Ensino m� dio completo (colegial). Sudeste Casado(a) / Vive junto 8 Feminino N� o Sim Sim 108 164 N� o Sim, � s vezes. N� o Menos de 1 ano. N� o, n� o tenho nenhuma dessas doen� as 5 est� mago raio x, vazio durar, horas pequeno, caf� a meia-noite, comer, nada, fazer caf� da manh� beba, � gua pergunta, o departamento poder compreendendo, transmitir, importantes, diagn� stico entendi, altera� � o, dez, ciente que, adaptar, direito, solicitar, telefonando precisar, instru� � es, procurar, Servi� o, esclarecer, d� vidas 13 N� o 14/01/2021 11 horas da manh� Sim Sim Sim Sim

1/10/2021 8:48:23 N� o SIM, aceito participar. Mi 12/10/1977 Sim Ensino m� dio completo (colegial). Sudeste Casado(a) / Vive junto 3 Feminino N� o Sim Sim 116 170 N� o Sim, � s vezes. N� o Mais de 10 anos. Outra que n� o est� entre as op� � es de resposta 6 pontos raio x, vazio durar, horas pequeno, caf� a meia-noite, comer, nada, fazer caf� da manh� beba, � gua pergunta, o departamento poder compreendendo, transmitir, importantes, diagn� stico entendi, altera� � o, dez, ciente que, adaptar, direito, solicitar, telefonando precisar, instru� � es, procurar, Servi� o, esclarecer, d� vidas 13 N� o 14/01/2021 11 Sim Sim Sim N� o

1/11/2021 20:36:37 N� o SIM, aceito participar. Br 17/01/1995 Sim Ensino fundamental completo. Sudeste Solteiro(a) 4 Feminino N� o Sim Sim 135 165 Sim Sim, � s vezes. Sim 1 a 5 anos. N� o, n� o tenho nenhuma dessas doen� as 8 est� mago raio x, vazio durar, horas pequeno, caf� a meia-noite, comer, nada, fazer caf� da manh� beba, � gua pergunta, o departamento poder compreendendo, transmitir, importantes, diagn� stico entendi, altera� � o, dez, ciente que, adaptar, direito, solicitar, telefonando precisar, instru� � es, procurar, Servi� o, esclarecer, d� vidas 13 hrs N� o 11/01/2021 11 hrs N� o N� o N� o N� o

1/12/2021 6:19:44 N� o SIM, aceito participar. Ra 04/01/1984 Sim Ensino m� dio completo (colegial). Centro-Oeste Casado(a) / Vive junto 3 Feminino Sim Sim Sim 110 173 N� o N� o. N� o Mais de 10 anos. Diabetes, Outra que n� o est� entre as op� � es de resposta 5 est� mago raio x, vazio durar, horas pequeno, caf� a meia-noite, comer, nada, fazer caf� da manh� beba, � gua pergunta, o departamento poder compreendendo, transmitir, importantes, diagn� stico entendi, altera� � o, dez, ciente que, adaptar, direito, solicitar, telefonando precisar, instru� � es, procurar, Servi� o, esclarecer, d� vidas 13hrs N� o 14/01/2021 As 11hrs Sim Sim Sim Sim

1/14/2021 14:54:54 N� o SIM, aceito participar. Ju 1975 Sim Ensino m� dio completo (colegial). Sudeste Casado(a) / Vive junto 3 Feminino Sim Sim Sim 100 159 N� o Sim, � s vezes. N� o 5 a 10 anos. N� o, n� o tenho nenhuma dessas doen� as 1 est� mago raio x, vazio durar, horas pequeno, caf� a meia-noite, comer, nada, fazer caf� da manh� beba, � gua pergunta, o departamento poder compreendendo, transmitir, importantes, diagn� stico entendi, altera� � o, dez, ciente que, adaptar, direito, solicitar, telefonando precisar, instru� � es, procurar, Servi� o, esclarecer, d� vidas 13:00 N� o 17/09/2020 11:00hs. N� o Sim N� o N� o

1/14/2021 17:34:28 N� o SIM, aceito participar. El 1973 Sim P� s-Gradua� � o (especializa� � o, mestrado e/ou doutorado). Sudeste Casado(a) / Vive junto 5 Feminino Sim Sim Sim 80 170 N� o N� o. N� o Menos de 1 ano. Outra que n� o est� entre as op� � es de resposta 3 est� mago raio x, vazio durar, horas pequeno, caf� a meia-noite, comer, nada, fazer caf� da manh� beba, � gua pergunta, o departamento poder compreendendo, transmitir, importantes, diagn� stico entendi, altera� � o, dez, ciente que, adaptar, direito, solicitar, telefonando precisar, instru� � es, procurar, Servi� o, esclarecer, d� vidas 13 N� o 14/07/2021 1 N� o N� o N� o N� o

1/14/2021 18:21:40 N� o SIM, aceito participar. Ca 71 Sim Ensino m� dio completo (colegial). Sudeste Casado(a)

/ Vive junto 3 Feminino Sim Sim Sim 96 159 N□ o N□ o. Sim 1 a 5 anos. N□ o, n□ o tenho nenhuma dessas doen
□ as 3 est□ mago raio x, vazio durar, horas pequeno, caf□ a meia-noite, comer, nada, fazer caf□ da manh□ beba,
□ gua pergunta, o departamento poder compreendo, transmitir, importantes, diagn□ stico entendi, altera□ □ o, dez,
ciente que, adaptar, direito, solicitar, telefonando precisar, instru□ □ es, procurar, Servi□ o, esclarecer, d□ vidas 13
horas N□ o 21/01/2021 11horas N□ o N□ o N□ o N□ o
1/15/2021 1:03:13 N□ o SIM, aceito participar. Te 1976 Sim Ensino superior completo (faculdade/universidade). Su
deste Solteiro(a) 2 Feminino Sim Sim Sim 80 163 N□ o N□ o. N□ o 1 a 5 anos. N□ o, n□ o tenho nenhuma dessas
doen□ as 2 est□ mago raio x, vazio durar, horas pequeno, caf□ a meia-noite, comer, nada, fazer caf□ da manh□ b
eba, □ gua pergunta, o departamento poder compreendo, transmitir, importantes, diagn□ stico entendi, altera□ □ o,
dez, ciente que, adaptar, direito, solicitar, telefonando precisar, instru□ □ es, procurar, Servi□ o, esclarecer, d□ vida
s As 13 N□ o 21/01/2021 As 11 N□ o Sim N□ o N□ o
1/15/2021 1:30:10 N□ o SIM, aceito participar. Mi 1975 Sim Ensino superior completo (faculdade/universidade). N
ordeste Casado(a) / Vive junto 3 Feminino Sim Sim Sim 79 157 N□ o Sim, □ s vezes. Sim 5 a 10 anos. Diabetes 4 e
st□ mago raio x, vazio durar, horas pequeno, caf□ a meia-noite, comer, nada, fazer caf□ da manh□ beba, □ gua p
ergunta, o departamento poder compreendo, transmitir, importantes, diagn□ stico entendi, altera□ □ o, dez, ciente q
ue, adaptar, direito, solicitar, telefonando precisar, instru□ □ es, procurar, Servi□ o, esclarecer, d□ vidas 13 horas N
□ o 21/01/2021 11 horas N□ o N□ o N□ o N□ o
1/15/2021 1:31:37 N□ o SIM, aceito participar. Th 1979 Sim P□ s-Gradua□ □ o (especializa□ □ o, mestrado e/ou
doutorado). Sul Casado(a) / Vive junto 3 Feminino Sim Sim Sim 80 172 N□ o Sim, □ s vezes. Sim Menos de 1 ano.
N□ o, n□ o tenho nenhuma dessas doen□ as 1 pontos raio x, vazio durar, horas pequeno, caf□ a meia-noite, comer
, nada, fazer caf□ da manh□ beba, □ gua pergunta, o departamento poder compreendo, transmitir, importantes, dia
gn□ stico entendi, altera□ □ o, dez, ciente que, adaptar, direito, solicitar, telefonando precisar, instru□ □ es, procura
r, Servi□ o, esclarecer, d□ vidas 13 N□ o 14/01/2021 12 Sim Sim Sim Sim
1/15/2021 5:19:19 N□ o SIM, aceito participar. MA 1992 Sim Ensino superior completo (faculdade/universidade). S
udeste Solteiro(a) 5 Feminino N□ o Sim Sim 76 173 N□ o N□ o. Sim Menos de 1 ano. N□ o, n□ o tenho nenhuma
dessas doen□ as 0 diabetes raio x, vazio durar, horas pequeno, caf□ a meia-noite, comer, nada, fazer caf□ da manh
□ beba, □ gua pergunta, o departamento poder compreendo, transmitir, importantes, diagn□ stico entendi, altera□
□ o, dez, ciente que, adaptar, direito, solicitar, telefonando precisar, instru□ □ es, procurar, Servi□ o, esclarecer, d
□ vidas 13h N□ o 21/01/2021 11h N□ o N□ o N□ o N□ o
1/15/2021 9:39:42 N□ o SIM, aceito participar. MJ 1976 Sim Ensino superior completo (faculdade/universidade). N
ordeste Casado(a) / Vive junto 3 Masculino Sim Sim Sim 110 170 N□ o Sim, □ s vezes. N□ o Mais de 10 anos. Dia
betes, Doen□ a nos rins 2 est□ mago raio x, vazio durar, horas pequeno, caf□ a meia-noite, comer, nada, fazer caf
□ da manh□ beba, □ gua pergunta, o departamento poder compreendo, transmitir, importantes, diagn□ stico enten
di, altera□ □ o, dez, ciente que, adaptar, direito, solicitar, telefonando precisar, instru□ □ es, procurar, Servi□ o, esc
larecer, d□ vidas 13 horas N□ o 21/01/2021 11 horas Sim Sim N□ o N□ o
1/15/2021 10:29:41 N□ o SIM, aceito participar. TA 1989 Sim P□ s-Gradua□ □ o (especializa□ □ o, mestrado e/ou
doutorado). Nordeste Casado(a) / Vive junto 3 Feminino Sim Sim Sim 82 154 N□ o Sim, □ s vezes. N□ o Menos d
e 1 ano. N□ o, n□ o tenho nenhuma dessas doen□ as 0 est□ mago raio x, vazio durar, horas pequeno, caf□ a meia-
noite, comer, nada, fazer caf□ da manh□ beba, □ gua pergunta, o departamento poder compreendo, transmitir, imp
ortantes, diagn□ stico entendi, altera□ □ o, dez, ciente que, adaptar, direito, solicitar, telefonando precisar, instru□
□ es, procurar, Servi□ o, esclarecer, d□ vidas 13 N□ o 21/01/2021 11:30 Sim Sim Sim Sim
1/15/2021 10:49:46 Sim SIM, aceito participar. Jo 1990 Sim Ensino superior completo (faculdade/universidade). No
rte Casado(a) / Vive junto 3 Masculino N□ o Sim Sim 97 175 N□ o Sim, □ s vezes. Sim Menos de 1 ano. N□ o, n
□ o tenho nenhuma dessas doen□ as 0 vermes raio x, vazio durar, horas pequeno, caf□ a meia-noite, comer, nada, f
azer caf□ da manh□ beba, □ gua pergunta, o departamento poder compreendo, transmitir, importantes, diagn□ stic
o entendi, altera□ □ o, dez, ciente que, adaptar, direito, solicitar, telefonando precisar, instru□ □ es, procurar, Servi
□ o, esclarecer, d□ vidas 13h N□ o 21/01/2021 11h30 N□ o N□ o N□ o Sim
1/15/2021 10:57:23 N□ o SIM, aceito participar. Cr 1987 Sim Ensino m□ dio completo (colegial). Centro-Oeste Ca
sado(a) / Vive junto 5 Feminino Sim Sim Sim 90 167 N□ o N□ o. N□ o 5 a 10 anos. N□ o, n□ o tenho nenhuma de
ssas doen□ as 0 pontos raio x, vazio durar, horas pequeno, caf□ a meia-noite, comer, nada, fazer caf□ da manh□
beba, □ gua pergunta, o departamento poder compreendo, transmitir, importantes, diagn□ stico entendi, altera□ □ o,
dez, ciente que, adaptar, direito, solicitar, telefonando precisar, instru□ □ es, procurar, Servi□ o, esclarecer, d□ vid
as 13:00 N□ o 21/01/2021 7:00 Sim Sim Sim Sim
1/15/2021 11:04:18 N□ o SIM, aceito participar. Ro 1974 Sim P□ s-Gradua□ □ o (especializa□ □ o, mestrado e/ou
doutorado). Sudeste Separado(a) / Divorciado(a) 1 Feminino Sim Sim Sim 89 157 N□ o N□ o. Sim Menos de 1 ano

. Outra que n□ o est□ entre as op□ □ es de resposta 2 pontos raio x, vazio durar, horas pequeno, caf□ a meia-noite
, comer, nada, fazer caf□ da manh□ beba, □ gua pergunta, o departamento poder compreendo, transmitir, importan
tes, diagn□ stico entendi, altera□ □ o, dez, ciente que, adaptar, direito, solicitar, telefonando precisar, instru□ □ es,
procurar, Servi□ o, esclarecer, d□ vidas 13 N□ o 21/01/2021 11 Sim N□ o N□ o Sim
1/15/2021 12:44:39 N□ o SIM, aceito participar. DA 1970 Sim P□ s-Gradua□ □ o (especializa□ □ o, mestrado e/o
u doutorado). Nordeste Separado(a) / Divorciado(a) 4 Feminino Sou aposentado(a) / pensionista Sim Sim 66 160 N
□ o N□ o. N□ o 5 a 10 anos. N□ o, n□ o tenho nenhuma dessas doen□ as 3 est□ mago raio x, vazio durar, horas pe
queno, caf□ a meia-noite, comer, nada, fazer caf□ da manh□ beba, □ gua pergunta, o departamento poder compre
endo, transmitir, importantes, diagn□ stico entendi, altera□ □ o, dez, ciente que, adaptar, direito, solicitar, telefonan
do precisar, instru□ □ es, procurar, Servi□ o, esclarecer, d□ vidas 13 N□ o 21/01/2021 11 N□ o Sim N□ o N□ o

1/15/2021 14:13:57 N□ o SIM, aceito participar. AD 1972 Sim P□ s-Gradua□ □ o (especializa□ □ o, mestrado e/o
u doutorado). Nordeste Casado(a) / Vive junto 4 Feminino Sim Sim Sim 47 147 N□ o N□ o. Sim 1 a 5 anos. Outra q
ue n□ o est□ entre as op□ □ es de resposta 3 est□ mago raio x, vazio durar, horas pequeno, caf□ a meia-noite, co
mer, nada, fazer caf□ da manh□ beba, □ gua pergunta, o departamento poder compreendo, transmitir, importantes,
diagn□ stico entendi, altera□ □ o, dez, ciente que, adaptar, direito, solicitar, telefonando precisar, instru□ □ es, pro
curar, Servi□ o, esclarecer, d□ vidas 13 N□ o 21/01/2021 11 N□ o N□ o N□ o N□ o
1/15/2021 17:17:10 N□ o SIM, aceito participar. An 1981 Sim Ensino m□ dio completo (colegial). Nordeste Casado
(a) / Vive junto 5 Feminino N□ o Sim Sim 106 167 N□ o N□ o. N□ o 1 a 5 anos. N□ o, n□ o tenho nenhuma dessa
s doen□ as 4 est□ mago raio x, vazio durar, horas pequeno, caf□ a meia-noite, comer, nada, fazer caf□ da manh□
beba, □ gua pergunta, o departamento poder compreendo, transmitir, importantes, diagn□ stico entendi, altera□ □ o,
dez, ciente que, adaptar, direito, solicitar, telefonando precisar, instru□ □ es, procurar, Servi□ o, esclarecer, d□ vid
as Uma da manh□ N□ o 14/01/2021 11 Sim Sim N□ o N□ o
1/15/2021 21:17:22 N□ o SIM, aceito participar. Iz 1953 Sim P□ s-Gradua□ □ o (especializa□ □ o, mestrado e/ou
doutorado). Sudeste Vi□ vo(a) 3 Feminino Sou aposentado(a) / pensionista Sim Sim 64 165 N□ o Sim, □ s vezes. S
im Mais de 10 anos. Diabetes, Outra que n□ o est□ entre as op□ □ es de resposta 5 est□ mago raio x, vazio durar,
horas pequeno, caf□ a meia-noite, comer, nada, fazer caf□ da manh□ beba, □ gua pergunta, o departamento poder
compreendo, transmitir, importantes, diagn□ stico entendi, altera□ □ o, dez, ciente que, adaptar, direito, solicitar, te
lefonando precisar, instru□ □ es, procurar, Servi□ o, esclarecer, d□ vidas 13 N□ o 17/09/2020 11 N□ o N□ o N□ o
Sim
1/16/2021 0:53:20 N□ o SIM, aceito participar. De 1985 Sim Ensino m□ dio completo (colegial). Nordeste Solteiro(
a) 5 Feminino N□ o Sim Sim 100 169 N□ o N□ o. N□ o Mais de 10 anos. Diabetes, Doen□ a nos rins, Outra que n
□ o est□ entre as op□ □ es de resposta 2 diabetes raio x, vazio durar, horas pequeno, caf□ antes, pedir, algum, esta
r caf□ da manh□ beba, □ gua tarefa, a farm□ cia poder compreendo, transmitir, importantes, diagn□ stico entendi,
altera□ □ o, dez, ciente que, adaptar, direito, solicitar, telefonando precisar, instru□ □ es, procurar, Servi□ o, esclar
ecer, d□ vidas 13 N□ o 14/02/2021 9 Sim Sim Sim Sim
1/16/2021 1:38:03 N□ o SIM, aceito participar. Ri 1959 Sim Ensino m□ dio completo (colegial). Sudeste Casado(a)
/ Vive junto 2 Masculino Sou aposentado(a) / pensionista Sim Sim 96 180 N□ o Sim, □ s vezes. N□ o Mais de 10 a
nos. Infarto, Doen□ a arterial coronariana (fiz cateterismo nos vasos do cora□ □ o) 8 pontos raio x, vazio durar, hor
as pequeno, caf□ a meia-noite, comer, nada, fazer caf□ da manh□ beba, □ gua pergunta, o departamento poder co
mpreendo, transmitir, importantes, diagn□ stico entendi, altera□ □ o, dez, ciente que, adaptar, direito, solicitar, telef
onando precisar, instru□ □ es, procurar, Servi□ o, esclarecer, d□ vidas 13 N□ o 21/01/2021 11 N□ o N□ o N□ o N
□ o
1/16/2021 4:49:36 N□ o SIM, aceito participar. Ci 1977p Sim Ensino fundamental completo. Centro-Oeste Vi□ vo(
a) 1 Feminino Sou aposentado(a) / pensionista Sim Sim 110 170 N□ o Sim, □ s vezes. Sim Mais de 10 anos. Diabet
es 2 est□ mago raio x, vazio durar, horas pequeno, caf□ a meia-noite, comer, nada, fazer caf□ da manh□ beba, □
gua pergunta, o departamento poder compreendo, transmitir, importantes, diagn□ stico entendi, altera□ □ o, dez, cie
nte que, adaptar, direito, solicitar, telefonando precisar, instru□ □ es, procurar, Servi□ o, esclarecer, d□ vidas 13hs
N□ o 21/01/2021 11hs N□ o N□ o N□ o Sim
1/16/2021 6:11:24 N□ o SIM, aceito participar. LU 1957 Sim Ensino superior completo (faculdade/universidade). N
ordeste Casado(a) / Vive junto 2 Feminino Sou aposentado(a) / pensionista Sim Sim 55 158 N□ o N□ o. Sim Mais d
e 10 anos. N□ o, n□ o tenho nenhuma dessas doen□ as 3 est□ mago raio x, vazio durar, horas pequeno, caf□ a mei
a-noite, comer, nada, fazer caf□ da manh□ beba, □ gua pergunta, o departamento poder compreendo, transmitir, i
mportantes, diagn□ stico entendi, altera□ □ o, dez, ciente que, adaptar, direito, solicitar, telefonando precisar, instru
□ □ es, procurar, Servi□ o, esclarecer, d□ vidas 13 N□ o 21/01/2021 11:00 N□ o N□ o N□ o Sim

1/16/2021 10:03:06 N□ o SIM, aceito participar. MA 1975 Sim Ensino m□ dio completo (colegial). Sudeste Separa
do(a) / Divorciado(a) 2 Feminino N□ o Sim Sim 75 160 N□ o Sim, □ s vezes. N□ o 5 a 10 anos. Diabetes 4 est□ m
ago raio x, vazio durar, horas pequeno, caf□ a meia-noite, comer, nada, fazer caf□ da manh□ beba, □ gua pergunt
a, o departamento poder compreendo, transmitir, importantes, diagn□ stico entendi, altera□ □ o, dez, ciente que, ada
ptar, direito, solicitar, telefonando precisar, instru□ □ es, procurar, Servi□ o, esclarecer, d□ vidas 13 horas N□ o 21
/01/2021 11 horas Sim Sim N□ o Sim
1/16/2021 11:01:36 N□ o SIM, aceito participar. Ta 1950 Sim Ensino superior completo (faculdade/universidade). S
udeste Casado(a) / Vive junto 2 Feminino Sou aposentado(a) / pensionista Sim Sim 80 159 N□ o Sim, □ s vezes. N
□ o Mais de 10 anos. Diabetes 2 est□ mago raio x, vazio durar, horas pequeno, caf□ a meia-noite, comer, nada, faz
er caf□ da manh□ beba, □ gua pergunta, o departamento poder compreendo, transmitir, importantes, diagn□ stico
entendi, altera□ □ o, dez, ciente que, adaptar, direito, solicitar, telefonando precisar, instru□ □ es, procurar, Servi□
o, esclarecer, d□ vidas 13 N□ o 21/01/2021 11 N□ o N□ o N□ o N□ o
1/16/2021 18:11:56 N□ o SIM, aceito participar. Ja 11.09.1999 Sim N□ o frequentei escola. Sudeste Solteiro(a) 3 F
eminino Sou aposentado(a) / pensionista Sim Sim 21 128 N□ o N□ o. N□ o 5 a 10 anos. Outra que n□ o est□ entre
as op□ □ es de resposta 5 est□ mago raio x, vazio durar, horas pequeno, caf□ a meia-noite, comer, nada, fazer caf
□ da manh□ beba, □ gua resposta, disque poder compreendo, transmitir, importantes, diagn□ stico entendi, altera
□ □ o, dez, ciente que, adaptar, direito, solicitar, telefonando precisar, instru□ □ es, procurar, Servi□ o, esclarecer,
d□ vidas 9 horas N□ o 14/01/2021 09 horas N□ o N□ o N□ o Sim
1/16/2021 22:48:24 N□ o SIM, aceito participar. Ma 1954 Sim Ensino m□ dio completo (colegial). Sudeste Vi□ vo(
a) 3 Feminino Sou aposentado(a) / pensionista Sim Sim 74 162 Sim N□ o. N□ o Mais de 10 anos. N□ o, n□ o tenho
nenhuma dessas doen□ as 7 est□ mago raio x, vazio durar, horas pequeno, caf□ a meia-noite, comer, nada, fazer c
af□ da manh□ beba, □ gua pergunta, o departamento poder compreendo, transmitir, importantes, diagn□ stico ente
ndi, altera□ □ o, dez, ciente que, adaptar, direito, solicitar, telefonando precisar, instru□ □ es, procurar, Servi□ o, es
clarecer, d□ vidas 13h N□ o 21/01/2021 11h N□ o N□ o N□ o N□ o
1/16/2021 23:14:38 N□ o SIM, aceito participar. DP 1978 Sim P□ s-Gradua□ □ o (especializa□ □ o, mestrado e/ou
doutorado). Sudeste Solteiro(a) 4 Masculino Sim Sim Sim 92 170 N□ o N□ o. Sim Mais de 10 anos. Outra que n□
o est□ entre as op□ □ es de resposta 4 est□ mago raio x, vazio durar, horas pequeno, caf□ a meia-noite, comer, na
da, fazer caf□ da manh□ beba, □ gua pergunta, o departamento poder compreendo, transmitir, importantes, diagn
□ stico entendi, altera□ □ o, dez, ciente que, adaptar, direito, solicitar, telefonando precisar, instru□ □ es, procurar,
Servi□ o, esclarecer, d□ vidas 13 N□ o 21/01/2021 11 N□ o N□ o N□ o Sim
1/17/2021 5:28:42 N□ o SIM, aceito participar. Ca 1960 Sim Ensino superior completo (faculdade/universidade). N
ordeste Solteiro(a) 1 Feminino N□ o Sim Sim 70 158 N□ o N□ o. N□ o Mais de 10 anos. Diabetes, Outra que n□ o
est□ entre as op□ □ es de resposta 10 est□ mago raio x, vazio durar, horas pequeno, caf□ a meia-noite, comer, nad
a, fazer caf□ da manh□ beba, □ gua pergunta, o departamento poder compreendo, transmitir, importantes, diagn□
stico entendi, altera□ □ o, dez, ciente que, adaptar, direito, solicitar, telefonando precisar, instru□ □ es, procurar, Se
rvi□ o, esclarecer, d□ vidas Ablock 10h N□ o 22/01/2021 10h N□ o Sim N□ o N□ o
1/17/2021 14:20:23 N□ o SIM, aceito participar. An 1971 Sim Ensino superior completo (faculdade/universidade).
Sudeste Casado(a) / Vive junto 4 Feminino Sim Sim Sim 110 170 N□ o N□ o. N□ o 5 a 10 anos. Diabetes, Outra qu
e n□ o est□ entre as op□ □ es de resposta 4 est□ mago raio x, vazio durar, horas pequeno, caf□ a meia-noite, com
er, nada, fazer caf□ da manh□ beba, □ gua pergunta, o departamento poder compreendo, transmitir, importantes, d
iagn□ stico entendi, altera□ □ o, dez, ciente que, adaptar, direito, solicitar, telefonando precisar, instru□ □ es, procu
rar, Servi□ o, esclarecer, d□ vidas 13 N□ o 21/01/2021 11 N□ o N□ o N□ o N□ o
1/17/2021 15:24:15 N□ o SIM, aceito participar. Lu 1964 Sim P□ s-Gradua□ □ o (especializa□ □ o, mestrado e/ou
doutorado). Sudeste Casado(a) / Vive junto 2 Feminino N□ o Sim Sim 53 163 N□ o N□ o. Sim 5 a 10 anos. N□ o,
n□ o tenho nenhuma dessas doen□ as 2 est□ mago raio x, vazio durar, horas pequeno, caf□ a meia-noite, comer, na
da, fazer caf□ da manh□ beba, □ gua pergunta, o departamento poder compreendo, transmitir, importantes, diagn
□ stico entendi, altera□ □ o, dez, ciente que, adaptar, direito, solicitar, telefonando precisar, instru□ □ es, procurar,
Servi□ o, esclarecer, d□ vidas 13 hs N□ o 21/01/2021 11 hs Sim N□ o N□ o N□ o
1/17/2021 16:20:25 N□ o SIM, aceito participar. Sa 1967 Sim Ensino superior completo (faculdade/universidade). S
udeste Casado(a) / Vive junto 3 Feminino N□ o Sim Sim 75 155 N□ o N□ o. Sim Mais de 10 anos. N□ o, n□ o ten
ho nenhuma dessas doen□ as 5 est□ mago raio x, vazio durar, horas pequeno, caf□ a meia-noite, comer, nada, fazer
caf□ da manh□ beba, □ gua pergunta, o departamento poder compreendo, transmitir, importantes, diagn□ stico en
tendi, altera□ □ o, dez, ciente que, adaptar, direito, solicitar, telefonando precisar, instru□ □ es, procurar, Servi□ o,
esclarecer, d□ vidas 13 N□ o 21/01/2021 11 N□ o N□ o N□ o N□ o
1/17/2021 18:32:35 N□ o SIM, aceito participar. CA 1998 Sim Ensino superior completo (faculdade/universidade).

Nordeste Solteiro(a) 7 Masculino Sim Sim Sim 140 180 Sim Sim, � s vezes. N� o 5 a 10 anos. N� o, n� o tenho nenhuma dessas doen� as 3 est� mago raio x, vazio durar, horas pequeno, caf� a meia-noite, comer, nada, fazer caf� da manh� beba, � gua pergunta, o departamento poder compreendo, transmitir, importantes, diagn� stico entendi, altera� � o, dez, ciente que, adaptar, direito, solicitar, telefonando precisar, instru� � es, procurar, Servi� o, esclarecer, d� vidas 13 Sim 28/04/2021 11 Sim Sim Sim Sim

1/17/2021 19:53:21 N� o SIM, aceito participar. Ma 1952 Sim Ensino superior completo (faculdade/universidade). Sudeste Casado(a) / Vive junto 2 Feminino Sou aposentado(a) / pensionista Sim Sim 64 160 N� o N� o. Sim Mais de 10 anos. Diabetes 2 est� mago raio x, vazio durar, horas pequeno, caf� a meia-noite, comer, nada, fazer caf� da manh� beba, � gua pergunta, o departamento poder compreendo, transmitir, importantes, diagn� stico entendi, altera� � o, dez, ciente que, adaptar, direito, solicitar, telefonando precisar, instru� � es, procurar, Servi� o, esclarecer, d� vidas 13 horas N� o 21/01/2021 07 N� o N� o N� o N� o

1/17/2021 23:27:27 N� o SIM, aceito participar. IS 1996 Sim Ensino m� dio completo (colegial). Sudeste Separado(a) / Divorciado(a) 4 Feminino Sou aposentado(a) / pensionista Sim Sim 105 169 N� o N� o. N� o 5 a 10 anos. Outra que n� o est� entre as op� � es de resposta 20 est� mago raio x, vazio durar, horas pequeno, caf� a meia-noite, comer, nada, fazer caf� da manh� beba, � gua pergunta, o departamento poder compreendo, transmitir, importantes, diagn� stico entendi, altera� � o, dez, ciente que, adaptar, direito, solicitar, telefonando precisar, instru� � es, procurar, Servi� o, esclarecer, d� vidas 13h N� o 21/01/2021 11h N� o N� o N� o N� o

1/18/2021 0:56:06 N� o SIM, aceito participar. SI 1970 Sim Ensino m� dio completo (colegial). Sudeste Solteiro(a) 3 Feminino Sou aposentado(a) / pensionista Sim N� o 42 140 N� o N� o. N� o 5 a 10 anos. N� o, n� o tenho nenhuma dessas doen� as 2 est� mago raio x, vazio durar, horas pequeno, caf� a meia-noite, comer, nada, fazer caf� da manh� beba, � gua pergunta, o departamento poder compreendo, transmitir, importantes, diagn� stico entendi, altera� � o, dez, ciente que, adaptar, direito, solicitar, telefonando precisar, instru� � es, procurar, Servi� o, esclarecer, d� vidas 14h N� o 21/01/2021 11h Sim Sim N� o N� o

1/18/2021 8:34:18 N� o SIM, aceito participar. RE 1976 Sim Ensino superior completo (faculdade/universidade). Sudeste Casado(a) / Vive junto 4 Feminino N� o Sim Sim 91 160 N� o N� o. N� o 5 a 10 anos. Diabetes 2 diabetes raio x, vazio durar, horas pequeno, caf� a meia-noite, comer, nada, fazer caf� da manh� beba, � gua pergunta, o departamento poder compreendo, transmitir, importantes, diagn� stico entendi, altera� � o, dez, ciente que, adaptar, direito, solicitar, telefonando precisar, instru� � es, procurar, Servi� o, esclarecer, d� vidas 13 Sim 21/01/2021 11 N� o Sim N� o Sim

1/18/2021 10:49:49 N� o SIM, aceito participar. Cl1963 1963 Sim Ensino m� dio completo (colegial). Nordeste Solteiro(a) 3 Feminino N� o Sim Sim 69 149 Sim N� o. N� o Mais de 10 anos. Diabetes, Outra que n� o est� entre as op� � es de resposta 12 est� mago raio x, vazio durar, horas pequeno, caf� a meia-noite, comer, nada, fazer caf� da manh� beba, � gua pergunta, o departamento poder compreendo, transmitir, importantes, diagn� stico entendi, altera� � o, dez, ciente que, adaptar, direito, solicitar, telefonando precisar, instru� � es, procurar, Servi� o, esclarecer, d� vidas 7 da noote Sim 17/02/2021 11h N� o N� o N� o N� o

1/18/2021 13:45:22 N� o SIM, aceito participar. JO 1958 Sim Ensino m� dio completo (colegial). Sudeste Casado(a) / Vive junto 3 Feminino Sou aposentado(a) / pensionista Sim Sim 68 163 N� o N� o. N� o Mais de 10 anos. N� o, n� o tenho nenhuma dessas doen� as 1 est� mago raio x, vazio durar, horas pequeno, caf� a meia-noite, comer, nada, fazer caf� da manh� beba, � gua pergunta, o departamento poder compreendo, transmitir, importantes, diagn� stico entendi, altera� � o, dez, ciente que, adaptar, direito, solicitar, telefonando precisar, instru� � es, procurar, Servi� o, esclarecer, d� vidas 13h N� o 21/01/2021 11h N� o N� o N� o N� o

1/18/2021 16:38:43 N� o SIM, aceito participar. Ed 1971 Sim P� s-Gradua� � o (especializa� � o, mestrado e/ou doutorado). Sudeste Casado(a) / Vive junto 02 Masculino Sim Sim Sim 96 169 N� o Sim, � s vezes. Sim 1 a 5 anos. N� o, n� o tenho nenhuma dessas doen� as 0 est� mago raio x, vazio durar, horas pequeno, caf� a meia-noite, comer, nada, fazer caf� da manh� beba, � gua pergunta, o departamento poder compreendo, transmitir, importantes, diagn� stico entendi, altera� � o, dez, ciente que, adaptar, direito, solicitar, telefonando precisar, instru� � es, procurar, Servi� o, esclarecer, d� vidas As 13:00 N� o 21/01/2021 As 11:00 horas Sim Sim N� o Sim

1/18/2021 18:50:09 N� o SIM, aceito participar. An Fortaleza Sim Ensino m� dio completo (colegial). Nordeste Casado(a) / Vive junto 5 Feminino Sim Sim Sim 85 162 N� o N� o. N� o 1 a 5 anos. Doen� a nos rins 2 est� mago raio x, vazio durar, horas pequeno, caf� a meia-noite, comer, nada, fazer caf� da manh� beba, � gua pergunta, o departamento poder compreendo, transmitir, importantes, diagn� stico entendi, altera� � o, dez, ciente que, adaptar, direito, solicitar, telefonando precisar, instru� � es, procurar, Servi� o, esclarecer, d� vidas 13 horas N� o 21/01/2021 11horas N� o N� o N� o N� o

1/18/2021 19:01:21 N� o SIM, aceito participar. NS 1963 Sim Ensino superior completo (faculdade/universidade). Sudeste Casado(a) / Vive junto 3 Feminino Sim Sim Sim 64 163 N� o Sim, � s vezes. Sim 1 a 5 anos. N� o, n� o t

enho nenhuma dessas doen� as 2 est� mago raio x, vazio durar, horas pequeno, caf� a meia-noite, comer, nada, fa zer caf� da manh� beba, � gua pergunta, o departamento poder compreendo, transmitir, importantes, diagn� stico entendi, altera� � o, dez, ciente que, adaptar, direito, solicitar, telefonando precisar, instru� � es, procurar, Servi � o, esclarecer, d� vidas 13 horas N� o 21/01/2021 12 horas N� o N� o N� o Sim
1/18/2021 19:28:14 N� o SIM, aceito participar. CL 1971 Sim Ensino superior completo (faculdade/universidade). Sudeste Solteiro(a) 2 Masculino Sim Sim Sim 78 170 N� o Sim, � s vezes. N� o Mais de 10 anos. N� o, n� o tenh o nenhuma dessas doen� as 0 est� mago raio x, vazio olhar, dietas pequeno, caf� a meia-noite, comer, nada, fazer caf� da manh� beba, � gua pergunta, o departamento poder compreendo, transmitir, importantes, diagn� stico ent endi, altera� � o, dez, ciente que, adaptar, direito, solicitar, telefonando precisar, instru� � es, procurar, Servi� o, e sclarecer, d� vidas 13 N� o 21/01/2021 11 N� o N� o N� o N� o
1/18/2021 20:28:44 N� o SIM, aceito participar. RO 1970 Sim P� s-Gradua� � o (especializa� � o, mestrado e/o u doutorado). Nordeste Casado(a) / Vive junto 4 Masculino Sim Sim Sim 74 162 N� o N� o. N� o Mais de 10 anos . N� o, n� o tenho nenhuma dessas doen� as 2 est� mago raio x, vazio durar, horas pequeno, caf� a meia-noite, c omer, nada, fazer caf� da manh� beba, � gua pergunta, o departamento poder compreendo, transmitir, importante s, diagn� stico entendi, altera� � o, dez, ciente do que, dispensar, errado, reparar, observando lavar, taxas, relaxar, Tumor, encobrir, p� lvis 13 horas N� o 21/01/2021 11 horas N� o N� o Sim N� o
1/18/2021 21:50:41 N� o SIM, aceito participar. So 1974 Sim Ensino fundamental completo. Norte Separado(a) / D ivorciado(a) 4 Feminino Sim Sim Sim 96 170 N� o N� o. N� o Menos de 1 ano. Diabetes, Outra que n� o est� en tre as op� � es de resposta 3 est� mago raio x, vazio durar, horas pequeno, caf� a meia-noite, comer, nada, fazer c af� da manh� beba, � gua pergunta, o departamento poder compreendo, transmitir, importantes, diagn� stico ente ndi, altera� � o, dez, ciente que, adaptar, direito, solicitar, telefonando precisar, instru� � es, procurar, Servi� o, es clarecer, d� vidas 13h N� o 21/01/2021 11h Sim Sim N� o N� o
1/18/2021 22:12:14 N� o SIM, aceito participar. LI 1985 Sim P� s-Gradua� � o (especializa� � o, mestrado e/ou doutorado). Sudeste Solteiro(a) 2 Feminino Sim Sim Sim 110 163 N� o N� o. Sim 5 a 10 anos. N� o, n� o tenho n enhuma dessas doen� as 1 est� mago raio x, vazio durar, horas pequeno, caf� a meia-noite, comer, nada, fazer caf � da manh� beba, � gua pergunta, o departamento poder compreendo, transmitir, importantes, diagn� stico enten di, altera� � o, dez, ciente que, adaptar, direito, solicitar, telefonando precisar, instru� � es, procurar, Servi� o, esc larecer, d� vidas 13 horas N� o 21/01/2021 11 horas Sim N� o N� o N� o
1/19/2021 0:03:59 N� o SIM, aceito participar. Ca 1967 Sim Ensino superior completo (faculdade/universidade). Su deste Casado(a) / Vive junto 2 Feminino N� o Sim Sim 119 167 N� o N� o. Sim 1 a 5 anos. Outra que n� o est� entre as op� � es de resposta 2 est� mago raio x, vazio durar, horas pequeno, caf� a meia-noite, comer, nada, faze r caf� da manh� beba, � gua pergunta, o departamento poder compreendo, transmitir, importantes, diagn� stico e ntendi, altera� � o, dez, ciente que, adaptar, direito, solicitar, telefonando precisar, instru� � es, procurar, Servi� o , esclarecer, d� vidas 13 hs N� o 21/01/2021 11 hs N� o Sim N� o N� o
1/19/2021 0:09:56 N� o SIM, aceito participar. JN 1997 Sim Ensino m� dio completo (colegial). Norte Solteiro(a) 5 Feminino N� o Sim Sim 98 172 Sim N� o. Sim 1 a 5 anos. N� o, n� o tenho nenhuma dessas doen� as 3 est� m ago raio x, vazio durar, horas pequeno, caf� a meia-noite, comer, nada, fazer caf� da manh� beba, � gua pergunt a, o departamento poder compreendo, transmitir, importantes, diagn� stico entendi, altera� � o, dez, ciente que, ada ptar, direito, solicitar, telefonando precisar, instru� � es, procurar, Servi� o, esclarecer, d� vidas As 14 horas Sim 21/01/2021 As 9 da manh� Sim Sim N� o N� o
1/19/2021 8:04:09 N� o SIM, aceito participar. Ha 1964 Sim P� s-Gradua� � o (especializa� � o, mestrado e/ou doutorado). Sudeste Casado(a) / Vive junto 2 Masculino Sim Sim Sim 99 180 N� o N� o. N� o 1 a 5 anos. Diabete s 3 est� mago raio x, vazio durar, horas pequeno, caf� a meia-noite, comer, nada, fazer caf� da manh� beba, � g ua pergunta, o departamento poder compreendo, transmitir, importantes, diagn� stico entendi, altera� � o, dez, cien te que, adaptar, direito, solicitar, telefonando precisar, instru� � es, procurar, Servi� o, esclarecer, d� vidas 13 N� o 21/01/2021 11 Sim Sim N� o N� o
1/19/2021 17:56:53 N� o SIM, aceito participar. Ma 1982 Sim Ensino m� dio completo (colegial). Sudeste Casado(a) / Vive junto 4 Feminino N� o Sim Sim 96 164 N� o N� o. Sim Mais de 10 anos. Infarto, Doen� a arterial coron ariana (fiz cateterismo nos vasos do cora� � o) 6 pontos raio x, vazio durar, horas pequeno, caf� a meia-noite, com er, nada, fazer caf� da manh� beba, � gua pergunta, o departamento poder compreendo, transmitir, importantes, d iagn� stico entendi, altera� � o, dez, ciente que, adaptar, direito, solicitar, telefonando precisar, instru� � es, procu rar, Servi� o, esclarecer, d� vidas 14 horas Sim 21/01/2021 11 Sim Sim N� o N� o
1/19/2021 18:14:39 N� o SIM, aceito participar. Jo 1966 Sim Ensino fundamental completo. Sudeste Casado(a) / Vi ve junto 3 Masculino Sim Sim Sim 89 165 N� o Sim, � s vezes. Sim Mais de 10 anos. Diabetes 1 est� mago raio x, vazio durar, horas pequeno, caf� a meia-noite, comer, nada, fazer caf� da manh� beba, � gua pergunta, o depart

amento poder compreendo, transmitir, importantes, diagn□ stico entendi, altera□ □ o, dez, ciente que, adaptar, direit
o, solicitar, telefonando precisar, instru□ □ es, procurar, Servi□ o, esclarecer, d□ vidas 13 horas N□ o 21/01/2021
11 horas N□ o Sim N□ o N□ o
1/19/2021 21:10:34 N□ o SIM, aceito participar. PA 1965 Sim P□ s-Gradua□ □ o (especializa□ □ o, mestrado e/ou
doutorado). Nordeste Solteiro(a) 2 Masculino Sim Sim Sim 84 173 N□ o Sim, frequentemente. Sim 5 a 10 anos. N
□ o, n□ o tenho nenhuma dessas doen□ as 4 est□ mago raio x, vazio durar, horas pequeno, caf□ a meia-noite, com
er, nada, fazer caf□ da manh□ beba, □ gua pergunta, o departamento poder compreendo, transmitir, importantes, d
iagn□ stico entendi, altera□ □ o, dez, ciente que, adaptar, direito, solicitar, telefonando precisar, instru□ □ es, procu
rar, Servi□ o, esclarecer, d□ vidas 13 N□ o 21/01/2021 11 N□ o N□ o N□ o Sim
1/19/2021 21:17:51 N□ o SIM, aceito participar. Ca 1984 Sim P□ s-Gradua□ □ o (especializa□ □ o, mestrado e/ou
doutorado). Sul Casado(a) / Vive junto 2 Feminino Sim Sim Sim 68 157 N□ o Sim, □ s vezes. N□ o Mais de 10 an
os. N□ o, n□ o tenho nenhuma dessas doen□ as 1 est□ mago raio x, vazio durar, horas pequeno, caf□ a meia-noite,
comer, nada, fazer caf□ da manh□ beba, □ gua pergunta, o departamento poder compreendo, transmitir, important
es, diagn□ stico entendi, altera□ □ o, dez, ciente que, adaptar, direito, solicitar, telefonando precisar, instru□ □ es, p
rocurar, Servi□ o, esclarecer, d□ vidas 13 N□ o 21/01/2021 11 N□ o N□ o N□ o N□ o
1/19/2021 22:46:07 N□ o SIM, aceito participar. Al 1972 Sim Ensino m□ dio completo (colegial). Sudeste Vi□ vo(
a) 2 Feminino Sou aposentado(a) / pensionista Sim Sim 140 170 N□ o Sim, □ s vezes. N□ o Mais de 10 anos. N□ o
, n□ o tenho nenhuma dessas doen□ as 4 est□ mago raio x, vazio durar, horas pequeno, caf□ a meia-noite, comer,
nada, fazer caf□ da manh□ beba, □ gua pergunta, o departamento poder compreendo, transmitir, importantes, diag
n□ stico entendi, altera□ □ o, dez, ciente que, adaptar, direito, solicitar, telefonando precisar, instru□ □ es, procurar
, Servi□ o, esclarecer, d□ vidas 13 N□ o 20/01/2021 11 N□ o N□ o N□ o N□ o
1/19/2021 23:07:17 N□ o SIM, aceito participar. El 1966 Sim Ensino m□ dio completo (colegial). Sul Separado(a) /
Divorciado(a) 3 Feminino Sim Sim Sim 80 150 N□ o Sim, □ s vezes. N□ o 1 a 5 anos. N□ o, n□ o tenho nenhuma
dessas doen□ as 2 est□ mago raio x, vazio durar, horas pequeno, caf□ a meia-noite, comer, nada, fazer caf□ da ma
nh□ beba, □ gua pergunta, o departamento poder compreendo, transmitir, importantes, diagn□ stico entendi, altera
□ □ o, dez, ciente que, adaptar, direito, solicitar, telefonando precisar, instru□ □ es, procurar, Servi□ o, esclarecer,
d□ vidas 13 horas N□ o 21/01/2021 11 horas Sim Sim N□ o N□ o
1/19/2021 23:54:38 N□ o SIM, aceito participar. Vi 1962 Sim Ensino m□ dio completo (colegial). Sul Separado(a) /
Divorciado(a) 1 Feminino N□ o Sim Sim 109 170 N□ o N□ o. N□ o Menos de 1 ano. N□ o, n□ o tenho nenhuma
dessas doen□ as 4 pontos raio x, vazio durar, horas pequeno, caf□ a meia-noite, comer, nada, fazer caf□ da manh
□ beba, □ gua pergunta, o departamento poder compreendo, transmitir, importantes, diagn□ stico entendi, altera□
□ o, dez, ciente que, adaptar, direito, solicitar, telefonando precisar, instru□ □ es, procurar, Servi□ o, esclarecer, d
□ vidas 13hrs Sim 19/01/2021 11hrs N□ o N□ o N□ o N□ o
1/20/2021 1:04:55 N□ o SIM, aceito participar. La 1965 Sim P□ s-Gradua□ □ o (especializa□ □ o, mestrado e/ou d
outorado). Sudeste Vi□ vo(a) 3 Feminino Sou aposentado(a) / pensionista Sim Sim 66 174 N□ o N□ o. N□ o 1 a 5
anos. N□ o, n□ o tenho nenhuma dessas doen□ as 2 est□ mago raio x, vazio durar, horas pequeno, caf□ a meia-noi
te, comer, nada, fazer caf□ da manh□ beba, □ gua pergunta, o departamento poder compreendo, transmitir, import
antes, diagn□ stico entendi, altera□ □ o, dez, ciente que, adaptar, direito, solicitar, telefonando precisar, instru□ □ e
s, procurar, Servi□ o, esclarecer, d□ vidas 13 horas N□ o 21/01/2021 11 horas N□ o Sim Sim N□ o
1/20/2021 6:04:54 N□ o SIM, aceito participar. ML 1959 Sim Ensino m□ dio completo (colegial). Nordeste Vi□ vo
(a) 5 Feminino Sou aposentado(a) / pensionista Sim Sim 70 154 N□ o N□ o. N□ o Mais de 10 anos. Diabetes, Infart
o, Outra que n□ o est□ entre as op□ □ es de resposta 8 est□ mago raio x, vazio durar, horas pequeno, caf□ a meia
-noite, comer, nada, fazer caf□ da manh□ beba, □ gua pergunta, o departamento poder compreendo, transmitir, im
portantes, diagn□ stico entendi, altera□ □ o, dez, ciente que, adaptar, direito, solicitar, telefonando precisar, instru□
□ es, procurar, Servi□ o, esclarecer, d□ vidas 13 horas Sim 21/01/2021 11 horas Sim Sim Sim Sim
1/20/2021 6:17:31 N□ o SIM, aceito participar. Th 1984 Sim P□ s-Gradua□ □ o (especializa□ □ o, mestrado e/ou
doutorado). Sul Casado(a) / Vive junto 2 Masculino Sim Sim Sim 113 185 N□ o Sim, □ s vezes. Sim 1 a 5 anos. N
□ o, n□ o tenho nenhuma dessas doen□ as 2 est□ mago raio x, vazio durar, horas pequeno, caf□ a meia-noite, com
er, nada, fazer caf□ da manh□ beba, □ gua pergunta, o departamento poder compreendo, transmitir, importantes, d
iagn□ stico entendi, altera□ □ o, dez, ciente que, adaptar, direito, solicitar, telefonando precisar, instru□ □ es, procu
rar, Servi□ o, esclarecer, d□ vidas 13h N□ o 21/01/2021 11h N□ o N□ o N□ o Sim
1/20/2021 8:45:32 N□ o SIM, aceito participar. Maria Luiza 1960 Sim P□ s-Gradua□ □ o (especializa□ □ o, mestr
ado e/ou doutorado). Sudeste Vi□ vo(a) 4 Feminino Sim Sim Sim 60 155 N□ o Sim, □ s vezes. N□ o Mais de 10 an
os. N□ o, n□ o tenho nenhuma dessas doen□ as 3 est□ mago raio x, vazio durar, horas pequeno, caf□ a meia-noite,
comer, nada, fazer caf□ da manh□ beba, □ gua pergunta, o departamento poder compreendo, transmitir, important

es, diagn□ stico entendi, altera□ □ o, dez, ciente que, adaptar, direito, solicitar, telefonando precisar, instru□ □ es, p
rocurar, Servi□ o, esclarecer, d□ vidas 13 horas N□ o 21/01/2021 11 horas N□ o N□ o N□ o N□ o

1/20/2021 9:02:46 N□ o SIM, aceito participar. Va 62 Sim Ensino m□ dio completo (colegial). Centro-Oeste Separa
do(a) / Divorciado(a) 01 Feminino N□ o Sim Sim 68 163 N□ o N□ o. N□ o Mais de 10 anos. N□ o, n□ o tenho nen
huma dessas doen□ as 6 est□ mago raio x, vazio durar, horas pequeno, caf□ a meia-noite, comer, nada, fazer caf□
da manh□ beba, □ gua pergunta, o departamento poder compreendo, transmitir, importantes, diagn□ stico entendi,
altera□ □ o, dez, ciente que, adaptar, direito, solicitar, telefonando precisar, instru□ □ es, procurar, Servi□ o, esclar
ecer, d□ vidas 13 N□ o 20/01/2021 11 N□ o Sim N□ o N□ o

1/20/2021 9:36:51 N□ o SIM, aceito participar. So 1967 Sim Ensino superior completo (faculdade/universidade). Su
deste Separado(a) / Divorciado(a) 3 Feminino Sim Sim Sim 86 163 N□ o N□ o. N□ o 5 a 10 anos. Outra que n□ o e
st□ entre as op□ □ es de resposta 3 est□ mago raio x, vazio durar, horas pequeno, caf□ a meia-noite, comer, nada,
fazer caf□ da manh□ beba, □ gua pergunta, o departamento poder compreendo, transmitir, importantes, diagn□ sti
co entendi, altera□ □ o, dez, ciente que, adaptar, direito, solicitar, telefonando precisar, instru□ □ es, procurar, Servi
□ o, esclarecer, d□ vidas 13 N□ o 21/01/2021 11 N□ o Sim N□ o N□ o

1/20/2021 10:33:29 N□ o SIM, aceito participar. DI 1952 Sim Ensino m□ dio completo (colegial). Sul Casado(a) /
Vive junto 3 Feminino Sou aposentado(a) / pensionista Sim Sim 78 167 N□ o Sim, □ s vezes. N□ o 1 a 5 anos. N□
o, n□ o tenho nenhuma dessas doen□ as 3 est□ mago raio x, vazio durar, horas pequeno, caf□ a meia-noite, comer,
 nada, fazer caf□ da manh□ beba, □ gua pergunta, o departamento poder compreendo, transmitir, importantes, diag
n□ stico entendi, altera□ □ o, dez, ciente que, adaptar, direito, solicitar, telefonando precisar, instru□ □ es, procurar
, Servi□ o, esclarecer, d□ vidas 13 horas N□ o 21/01/2021 11 horas N□ o N□ o N□ o N□ o

1/20/2021 12:22:07 N□ o SIM, aceito participar. Fl 1980 Sim Ensino m□ dio completo (colegial). Nordeste Casado(
a) / Vive junto 4 Feminino Sim Sim Sim 43 162 N□ o Sim, □ s vezes. N□ o 1 a 5 anos. Outra que n□ o est□ entre
as op□ □ es de resposta, N□ o, n□ o tenho nenhuma dessas doen□ as 4 est□ mago raio x, vazio durar, horas pequen
o, caf□ a meia-noite, comer, nada, fazer caf□ da manh□ beba, □ gua pergunta, o departamento poder compreendo
, transmitir, importantes, diagn□ stico entendi, altera□ □ o, dez, ciente que, adaptar, direito, solicitar, telefonando pr
ecisar, instru□ □ es, procurar, Servi□ o, esclarecer, d□ vidas 13 horas N□ o 21/01/2021 11 horas N□ o N□ o N□ o
N□ o

1/20/2021 12:50:46 N□ o SIM, aceito participar. El 1965 Sim Ensino superior completo (faculdade/universidade). N
ordeste Casado(a) / Vive junto 3 Feminino Sim Sim Sim 76 165 N□ o N□ o. Sim 5 a 10 anos. N□ o, n□ o tenho nen
huma dessas doen□ as 1 est□ mago raio x, vazio durar, horas pequeno, caf□ a meia-noite, comer, nada, fazer caf□
da manh□ beba, □ gua pergunta, o departamento poder compreendo, transmitir, importantes, diagn□ stico entendi,
altera□ □ o, dez, ciente que, adaptar, direito, solicitar, telefonando precisar, instru□ □ es, procurar, Servi□ o, esclar
ecer, d□ vidas 19 noite N□ o 20/01/2021 15 horas Sim Sim Sim Sim

1/20/2021 13:57:40 N□ o SIM, aceito participar. AN 1961 Sim Ensino superior completo (faculdade/universidade).
Sul Separado(a) / Divorciado(a) 04 Feminino Sim Sim Sim 85 160 N□ o Sim, □ s vezes. N□ o 1 a 5 anos. N□ o, n
□ o tenho nenhuma dessas doen□ as 01 est□ mago raio x, vazio durar, horas pequeno, caf□ a meia-noite, comer, na
da, fazer caf□ da manh□ beba, □ gua pergunta, o departamento poder compreendo, transmitir, importantes, diagn
□ stico entendi, altera□ □ o, dez, ciente que, adaptar, direito, solicitar, telefonando precisar, instru□ □ es, procurar,
Servi□ o, esclarecer, d□ vidas 13 horas N□ o 21/01/2021 11 horas N□ o N□ o N□ o N□ o

1/20/2021 14:07:23 N□ o SIM, aceito participar. Vi 1961 Sim P□ s-Gradua□ □ o (especializa□ □ o, mestrado e/ou
doutorado). Sudeste Separado(a) / Divorciado(a) 3 Feminino Sim Sim Sim 97 155 N□ o Sim, □ s vezes. N□ o Men
os de 1 ano. N□ o, n□ o tenho nenhuma dessas doen□ as 4 est□ mago raio x, vazio durar, horas pequeno, caf□ a m
eia-noite, comer, nada, fazer caf□ da manh□ beba, □ gua pergunta, o departamento poder compreendo, transmitir,
importantes, diagn□ stico entendi, altera□ □ o, dez, ciente que, adaptar, direito, solicitar, telefonando precisar, instr
u□ □ es, procurar, Servi□ o, esclarecer, d□ vidas 13hs N□ o 21/01/2021 11hs N□ o Sim Sim Sim

1/20/2021 16:23:05 N□ o SIM, aceito participar. RO 1973 Sim P□ s-Gradua□ □ o (especializa□ □ o, mestrado e/o
u doutorado). Sudeste Vi□ vo(a) 2 Feminino Sim Sim Sim 100 155 N□ o Sim, □ s vezes. N□ o Mais de 10 anos. N
□ o, n□ o tenho nenhuma dessas doen□ as 3 est□ mago raio x, vazio durar, horas pequeno, caf□ a meia-noite, com
er, nada, fazer caf□ da manh□ beba, □ gua pergunta, o departamento poder compreendo, transmitir, importantes, d
iagn□ stico entendi, altera□ □ o, dez, ciente que, adaptar, direito, solicitar, telefonando precisar, instru□ □ es, procu
rar, Servi□ o, esclarecer, d□ vidas 13 N□ o 21/01/2021 11 Sim Sim N□ o N□ o

1/20/2021 18:28:29 N□ o SIM, aceito participar. Jo 1960 Sim Ensino m□ dio completo (colegial). Sudeste Casado(a
) / Vive junto 4 Masculino Sou aposentado(a) / pensionista Sim Sim 100 185 N□ o Sim, □ s vezes. N□ o Mais de 10
 anos. AVC (derrame), Doen□ a arterial coronariana (fiz cateterismo nos vasos do cora□ □ o) 4 est□ mago raio x, v
azio durar, horas pequeno, caf□ a meia-noite, comer, nada, fazer caf□ da manh□ beba, □ gua pergunta, o departa

mento poder compreendo, transmitir, importantes, diagn□ stico entendi, altera□ □ o, dez, ciente que, adaptar, direito
, solicitar, telefonando precisar, instru□ □ es, procurar, Servi□ o, esclarecer, d□ vidas 13 horas N□ o 21/01/2021 11
horas N□ o Sim N□ o N□ o

1/20/2021 19:58:21 N□ o SIM, aceito participar. Jo 1963 Sim Ensino fundamental completo. Sudeste Casado(a) / Vi
ve junto 3 Feminino Sou aposentado(a) / pensionista Sim Sim 115 160 N□ o N□ o. N□ o Mais de 10 anos. Diabetes
, Doen□ a arterial coronariana (fiz cateterismo nos vasos do cora□ □ o) 15 est□ mago raio x, vazio durar, horas peq
ueno, caf□ a meia-noite, comer, nada, fazer caf□ da manh□ beba, □ gua pergunta, o departamento poder compree
ndo, transmitir, importantes, diagn□ stico entendi, altera□ □ o, dez, ciente que, adaptar, direito, solicitar, telefonand
o precisar, instru□ □ es, procurar, Servi□ o, esclarecer, d□ vidas 13 Sim 10/06/2021 11 N□ o N□ o N□ o Sim

1/20/2021 21:40:22 N□ o SIM, aceito participar. AN 1965 Sim P□ s-Gradua□ □ o (especializa□ □ o, mestrado e/o
u doutorado). Nordeste Separado(a) / Divorciado(a) 3 Feminino Sim Sim Sim 85 158 N□ o N□ o. N□ o Mais de 10
anos. Diabetes 4 est□ mago raio x, vazio durar, horas pequeno, caf□ a meia-noite, comer, nada, fazer caf□ da man
h□ beba, □ gua pergunta, o departamento poder compreendo, transmitir, importantes, diagn□ stico entendi, altera□
□ o, dez, ciente que, adaptar, direito, solicitar, telefonando precisar, instru□ □ es, procurar, Servi□ o, esclarecer, d
□ vidas 13horas N□ o 21/01/2021 11horas N□ o N□ o N□ o N□ o

1/20/2021 21:41:53 N□ o SIM, aceito participar. FA 1957 Sim Ensino m□ dio completo (colegial). Sudeste Casado(
a) / Vive junto 2 Feminino N□ o Sim Sim 80 165 N□ o Sim, □ s vezes. Sim Mais de 10 anos. Outra que n□ o est□
entre as op□ □ es de resposta 10 est□ mago raio x, vazio durar, horas pequeno, caf□ a meia-noite, comer, nada, faz
er caf□ da manh□ beba, □ gua pergunta, o departamento poder compreendo, transmitir, importantes, diagn□ stico
entendi, altera□ □ o, dez, ciente do que, dispensar, errado, reparar, observando precisar, instru□ □ es, procurar, Ser
vi□ o, esclarecer, d□ vidas 13 horas N□ o 21/01/2021 11 horas N□ o N□ o N□ o N□ o

1/21/2021 6:29:28 N□ o SIM, aceito participar. Ja 1964 Sim P□ s-Gradua□ □ o (especializa□ □ o, mestrado e/ou d
outorado). Sul Casado(a) / Vive junto 3 Feminino Sim Sim Sim 80 160 N□ o Sim, □ s vezes. Sim Mais de 10 anos.
Doen□ a arterial coronariana (fiz cateterismo nos vasos do cora□ □ o), Outra que n□ o est□ entre as op□ □ es de r
esposta 6 est□ mago raio x, vazio durar, horas pequeno, caf□ a meia-noite, comer, nada, fazer caf□ da manh□ beb
a, □ gua pergunta, o departamento poder compreendo, transmitir, importantes, diagn□ stico entendi, altera□ □ o, de
z, ciente que, adaptar, direito, solicitar, telefonando precisar, instru□ □ es, procurar, Servi□ o, esclarecer, d□ vidas
13 N□ o 21/01/2021 11 N□ o N□ o N□ o N□ o

1/21/2021 6:33:15 N□ o SIM, aceito participar. TA 1969 Sim Ensino m□ dio completo (colegial). Sudeste Solteiro(
a) 1 Feminino Sim Sim Sim 89 174 N□ o N□ o. N□ o Mais de 10 anos. Diabetes, Infarto, Doen□ a arterial coronari
ana (fiz cateterismo nos vasos do cora□ □ o) 8 pontos raio x, vazio durar, horas pequeno, caf□ a meia-noite, comer,
nada, fazer caf□ da manh□ beba, □ gua pergunta, o departamento poder compreendo, transmitir, importantes, diag
n□ stico entendi, altera□ □ o, dez, ciente que, adaptar, direito, solicitar, telefonando precisar, instru□ □ es, procurar
, Servi□ o, esclarecer, d□ vidas 13 N□ o 21/01/2021 11 Sim Sim N□ o N□ o

1/21/2021 6:49:47 N□ o SIM, aceito participar. Sa 1958 Sim Ensino m□ dio completo (colegial). Sudeste Casado(a)
/ Vive junto 3 Feminino Sou aposentado(a) / pensionista Sim Sim 45 150 Sim Sim, □ s vezes. N□ o Mais de 10 ano
s. N□ o, n□ o tenho nenhuma dessas doen□ as 2 est□ mago raio x, vazio durar, horas pequeno, caf□ a meia-noite,
comer, nada, fazer caf□ da manh□ beba, □ gua pergunta, o departamento poder compreendo, transmitir, important
es, diagn□ stico entendi, altera□ □ o, dez, ciente que, adaptar, direito, solicitar, telefonando precisar, instru□ □ es, p
rocurar, Servi□ o, esclarecer, d□ vidas 13 Sim 21/01/2021 11 Sim N□ o N□ o Sim

1/21/2021 10:53:24 N□ o SIM, aceito participar. Si 1965 Sim Ensino superior completo (faculdade/universidade). S
udeste Solteiro(a) 2 Feminino Sim Sim Sim 54 160 N□ o Sim, □ s vezes. Sim 5 a 10 anos. N□ o, n□ o tenho nenhu
ma dessas doen□ as 0 est□ mago raio x, vazio durar, horas pequeno, caf□ a meia-noite, comer, nada, fazer caf□ da
manh□ beba, □ gua pergunta, o departamento poder compreendo, transmitir, importantes, diagn□ stico entendi, alt
era□ □ o, dez, ciente que, adaptar, direito, solicitar, telefonando precisar, instru□ □ es, procurar, Servi□ o, esclarece
r, d□ vidas 13 hs N□ o 28/01/2021 11 hs Sim N□ o N□ o N□ o

1/21/2021 14:16:27 N□ o SIM, aceito participar. Su 1968 Sim P□ s-Gradua□ □ o (especializa□ □ o, mestrado e/ou
doutorado). Nordeste Solteiro(a) 4 Feminino N□ o Sim Sim 85 154 N□ o N□ o. N□ o 5 a 10 anos. N□ o, n□ o tenh
o nenhuma dessas doen□ as 1 est□ mago raio x, vazio durar, horas pequeno, caf□ a meia-noite, comer, nada, fazer
caf□ da manh□ beba, □ gua pergunta, o departamento poder compreendo, transmitir, importantes, diagn□ stico ent
endi, altera□ □ o, dez, ciente que, adaptar, direito, solicitar, telefonando precisar, instru□ □ es, procurar, Servi□ o, e
sclarecer, d□ vidas 13h N□ o 28/01/2021 6h Sim Sim Sim Sim

1/21/2021 14:18:47 N□ o SIM, aceito participar. Ma 66 Sim Ensino fundamental completo. Nordeste Solteiro(a) 5 F
eminino N□ o Sim Sim 59 153 N□ o N□ o. N□ o Mais de 10 anos. Diabetes 2 est□ mago dormir, anemia olhar, die
tas pensamento, n□ usea antes, pedir, algum, estar caf□ da manh□ beba, □ gua pergunta, o departamento doer com

preendo, transmitir, importantes, diagn□ stico entretenho, anti□ cido, um, longe do que, dispensar, errado, reparar, o
bservando precisar, instru□ □ es, procurar, Servi□ o, esclarecer, d□ vidas 1 da amanh□ N□ o 21/01/2021 11 Sim
N□ o N□ o N□ o
1/21/2021 14:56:59 N□ o SIM, aceito participar. Ja 1967 Sim Ensino m□ dio completo (colegial). Nordeste Solteiro
(a) 2 Feminino N□ o Sim Sim 120 163 N□ o Sim, □ s vezes. N□ o 5 a 10 anos. N□ o, n□ o tenho nenhuma dessas
doen□ as 3 est□ mago raio x, vazio durar, horas pequeno, caf□ a meia-noite, comer, nada, fazer caf□ da manh□ b
eba, □ gua pergunta, o departamento poder compreendo, transmitir, importantes, diagn□ stico entendi, altera□ □ o,
dez, ciente que, adaptar, direito, solicitar, telefonando precisar, instru□ □ es, procurar, Servi□ o, esclarecer, d□ vida
s 13h N□ o 04/02/2021 11h N□ o N□ o N□ o N□ o
1/21/2021 17:02:55 N□ o SIM, aceito participar. CL 1964 Sim Ensino superior completo (faculdade/universidade).
Sudeste Separado(a) / Divorciado(a) 3 Masculino Sou aposentado(a) / pensionista Sim Sim 120 170 N□ o Sim, freq
uentemente. Sim 5 a 10 anos. N□ o, n□ o tenho nenhuma dessas doen□ as 5 pontos raio x, vazio durar, horas peque
no, caf□ a meia-noite, comer, nada, fazer caf□ da manh□ beba, □ gua pergunta, o departamento poder compreend
o, transmitir, importantes, diagn□ stico entendi, altera□ □ o, dez, ciente que, adaptar, direito, solicitar, telefonando p
recisar, instru□ □ es, procurar, Servi□ o, esclarecer, d□ vidas 13 N□ o 28/01/2021 11 Sim Sim N□ o N□ o
1/21/2021 18:36:54 N□ o SIM, aceito participar. Ad 1963 Sim Ensino m□ dio completo (colegial). Nordeste Casado
(a) / Vive junto 2 Masculino Sim Sim Sim 106 170 N□ o Sim, □ s vezes. Sim Mais de 10 anos. N□ o, n□ o tenho n
enhuma dessas doen□ as 1 est□ mago raio x, vazio durar, horas pequeno, caf□ a meia-noite, comer, nada, fazer caf
□ da manh□ beba, □ gua pergunta, o departamento poder compreendo, transmitir, importantes, diagn□ stico enten
di, altera□ □ o, dez, ciente que, adaptar, direito, solicitar, telefonando precisar, instru□ □ es, procurar, Servi□ o, esc
larecer, d□ vidas 13 horas N□ o 21/01/2021 11 horas Sim Sim Sim N□ o
1/21/2021 18:41:19 N□ o SIM, aceito participar. Cl 1972 Sim Ensino superior completo (faculdade/universidade). S
udeste Vi□ vo(a) 3 Feminino N□ o Sim Sim 67 156 N□ o Sim, □ s vezes. Sim 5 a 10 anos. N□ o, n□ o tenho nenh
uma dessas doen□ as 2 est□ mago raio x, vazio durar, horas pequeno, caf□ a meia-noite, comer, nada, fazer caf□ d
a manh□ beba, □ gua pergunta, o departamento poder compreendo, transmitir, importantes, diagn□ stico entendi, al
tera□ □ o, dez, ciente que, adaptar, direito, solicitar, telefonando precisar, instru□ □ es, procurar, Servi□ o, esclarec
er, d□ vidas 13hs N□ o 21/01/2021 11hs Sim Sim N□ o N□ o
1/21/2021 19:29:36 N□ o SIM, aceito participar. He 1954 Sim Ensino fundamental completo. Sudeste Vi□ vo(a) 2
Masculino Sou aposentado(a) / pensionista Sim Sim 65 178 N□ o Sim, □ s vezes. N□ o 5 a 10 anos. Diabetes 4 est
□ mago raio x, vazio durar, horas pequeno, caf□ a meia-noite, comer, nada, fazer caf□ da manh□ beba, □ gua per
gunta, o departamento poder compreendo, transmitir, importantes, diagn□ stico entendi, altera□ □ o, dez, ciente que
, adaptar, direito, solicitar, telefonando precisar, instru□ □ es, procurar, Servi□ o, esclarecer, d□ vidas 13 N□ o 21/
01/2021 11 Sim Sim N□ o Sim
1/21/2021 20:12:49 N□ o SIM, aceito participar. AN 1969 Sim P□ s-Gradua□ □ o (especializa□ □ o, mestrado e/o
u doutorado). Norte Casado(a) / Vive junto 4 Feminino Sim Sim Sim 60 152 N□ o N□ o. Sim 5 a 10 anos. N□ o, n
□ o tenho nenhuma dessas doen□ as 5 est□ mago raio x, vazio durar, horas pequeno, caf□ a meia-noite, comer, nad
a, fazer caf□ da manh□ beba, □ gua pergunta, o departamento poder compreendo, transmitir, importantes, diagn□
stico entendi, altera□ □ o, dez, ciente que, adaptar, direito, solicitar, telefonando precisar, instru□ □ es, procurar, Se
rvi□ o, esclarecer, d□ vidas 13h N□ o 21/01/2021 11h N□ o Sim N□ o N□ o
1/21/2021 20:47:01 N□ o SIM, aceito participar. Na 1967 Sim Ensino m□ dio completo (colegial). Sudeste Casado(
a) / Vive junto 2 Feminino Sou aposentado(a) / pensionista Sim Sim 76 167 N□ o Sim, □ s vezes. N□ o Mais de 10
anos. N□ o, n□ o tenho nenhuma dessas doen□ as 4 est□ mago raio x, vazio durar, horas pequeno, caf□ a meia-noi
te, comer, nada, fazer caf□ da manh□ beba, □ gua pergunta, o departamento poder compreendo, transmitir, import
antes, diagn□ stico entendi, altera□ □ o, dez, ciente que, adaptar, direito, solicitar, telefonando precisar, instru□ □ e
s, procurar, Servi□ o, esclarecer, d□ vidas 13 N□ o 28/01/2021 11 N□ o N□ o N□ o N□ o
1/21/2021 21:19:41 N□ o SIM, aceito participar. An 67 Sim Ensino m□ dio completo (colegial). Sudeste Solteiro(a)
1 Feminino Sou aposentado(a) / pensionista Sim Sim 75 161 N□ o N□ o. Sim 5 a 10 anos. Diabetes 3 diabetes raio
x, vazio durar, horas pequeno, caf□ a meia-noite, comer, nada, fazer caf□ da manh□ beba, □ gua pergunta, o depa
rtamento poder compreendo, transmitir, importantes, diagn□ stico entendi, altera□ □ o, dez, ciente que, adaptar, dire
ito, solicitar, telefonando precisar, instru□ □ es, procurar, Servi□ o, esclarecer, d□ vidas 14 Sim 22/02/2021 11 N□
o N□ o N□ o N□ o
1/21/2021 21:56:00 N□ o SIM, aceito participar. Ya 1961 Sim P□ s-Gradua□ □ o (especializa□ □ o, mestrado e/ou
doutorado). Sudeste Casado(a) / Vive junto 2 Feminino N□ o Sim Sim 85 170 N□ o Sim, □ s vezes. Sim 5 a 10 ano
s. Diabetes 3 pontos raio x, vazio durar, horas pequeno, caf□ a meia-noite, comer, nada, fazer caf□ da manh□ beb
a, □ gua pergunta, o departamento poder compreendo, transmitir, importantes, diagn□ stico entendi, altera□ □ o, de

z, ciente que, adaptar, direito, solicitar, telefonando precisar, instru� � es, procurar, Servi� o, esclarecer, d� vidas 13 N� o 21/01/2021 11 N� o N� o N� o N� o
1/21/2021 22:03:23 N� o SIM, aceito participar. HU 1988 Sim P� s-Gradua� � o (especializa� � o, mestrado e/ou doutorado). Nordeste Solteiro(a) 4 Feminino Sim Sim Sim 76 163 N� o N� o. N� o 1 a 5 anos. N� o, n� o tenho nenhuma dessas doen� as 6 est� mago raio x, vazio durar, horas pequeno, caf� a meia-noite, comer, nada, fazer caf� da manh� beba, � gua pergunta, o departamento poder compreendo, transmitir, importantes, diagn� stico entendi, altera� � o, dez, ciente que, adaptar, direito, solicitar, telefonando precisar, instru� � es, procurar, Servi� o, esclarecer, d� vidas 13 N� o 28/01/2021 11 N� o Sim N� o N� o
1/21/2021 23:04:45 N� o SIM, aceito participar. Fl 1958 Sim Ensino superior completo (faculdade/universidade). Sul Solteiro(a) 4 Feminino Sou aposentado(a) / pensionista Sim Sim 80 170 N� o N� o. N� o Mais de 10 anos. Outra que n� o est� entre as op� � es de resposta 4 est� mago raio x, vazio durar, horas pequeno, caf� a meia-noite, comer, nada, fazer caf� da manh� beba, � gua pergunta, o departamento poder compreendo, transmitir, importantes, diagn� stico entendi, altera� � o, dez, ciente que, adaptar, direito, solicitar, telefonando precisar, instru� � es, procurar, Servi� o, esclarecer, d� vidas 13 horas N� o 28/01/2021 11 horas N� o N� o N� o Sim
1/21/2021 23:45:25 N� o SIM, aceito participar. RO 1976 Sim Ensino m� dio completo (colegial). Sudeste Casado(a) / Vive junto 5 Feminino Sim Sim Sim 139 139 N� o Sim, � s vezes. N� o 1 a 5 anos. Outra que n� o est� entre as op� � es de resposta 10 pontos raio x, vazio durar, horas pequeno, caf� a meia-noite, comer, nada, fazer caf� da manh� beba, � gua pergunta, o departamento poder compreendo, transmitir, importantes, diagn� stico entendi, altera� � o, dez, ciente que, adaptar, direito, solicitar, telefonando precisar, instru� � es, procurar, Servi� o, esclarecer, d� vidas 13 horas Sim 21/01/2021 11 horas N� o N� o N� o Sim
1/22/2021 1:55:34 N� o SIM, aceito participar. Si 1971 Sim Ensino m� dio completo (colegial). Sudeste Vi� vo(a) 3 Feminino Sim Sim Sim 100 158 Sim Sim, frequentemente. N� o Mais de 10 anos. Outra que n� o est� entre as op� � es de resposta 5 est� mago raio x, vazio durar, horas pequeno, caf� a meia-noite, comer, nada, fazer caf� da manh� beba, � gua pergunta, o departamento poder compreendo, transmitir, importantes, diagn� stico entendi, altera� � o, dez, ciente que, adaptar, direito, solicitar, telefonando precisar, instru� � es, procurar, Servi� o, esclarecer, d� vidas 13 N� o 28/01/2021 12 Sim Sim Sim Sim
1/22/2021 2:28:39 N� o SIM, aceito participar. MA 1980 Sim Ensino superior completo (faculdade/universidade). Centro-Oeste Solteiro(a) 2 Feminino N� o Sim Sim 100 150 N� o Sim, frequentemente. N� o Mais de 10 anos. N� o, n� o tenho nenhuma dessas doen� as 4 est� mago raio x, vazio durar, horas pequeno, caf� a meia-noite, comer, nada, fazer caf� da manh� beba, � gua pergunta, o departamento poder compreendo, transmitir, importantes, diagn� stico entendi, altera� � o, dez, ciente que, adaptar, direito, solicitar, telefonando precisar, instru� � es, procurar, Servi� o, esclarecer, d� vidas 13:00 N� o 28/01/2021 11:00 Sim Sim N� o N� o
1/22/2021 6:09:46 N� o SIM, aceito participar. Jo 1965 Sim P� s-Gradua� � o (especializa� � o, mestrado e/ou doutorado). Nordeste Casado(a) / Vive junto 3 Feminino Sim Sim Sim 73 165 N� o N� o. N� o 5 a 10 anos. N� o, n� o tenho nenhuma dessas doen� as 2 est� mago raio x, vazio durar, horas pequeno, caf� a meia-noite, comer, nada, fazer caf� da manh� beba, � gua pergunta, o departamento poder compreendo, transmitir, importantes, diagn� stico entendi, altera� � o, dez, ciente que, adaptar, direito, solicitar, telefonando precisar, instru� � es, procurar, Servi� o, esclarecer, d� vidas 13 horas N� o 25/02/2021 11 horas N� o N� o N� o Sim
1/22/2021 6:51:58 N� o SIM, aceito participar. Va 1968 Sim Ensino m� dio completo (colegial). Nordeste Separado(a) / Divorciado(a) 3 Feminino N� o Sim Sim 85 172 N� o Sim, � s vezes. N� o Mais de 10 anos. N� o, n� o tenho nenhuma dessas doen� as 4 est� mago raio x, vazio durar, horas pequeno, caf� a meia-noite, comer, nada, fazer caf� da manh� beba, � gua pergunta, o departamento poder compreendo, transmitir, importantes, diagn� stico entendi, altera� � o, dez, ciente que, adaptar, direito, solicitar, telefonando precisar, instru� � es, procurar, Servi� o, esclarecer, d� vidas 13hrs N� o 28/01/2021 11hrs Sim Sim N� o Sim
1/22/2021 8:03:42 N� o SIM, aceito participar. MS 1978 Sim Ensino fundamental completo. Sul Separado(a) / Divorciado(a) 4 Feminino N� o Sim Sim 73 162 Sim Sim, � s vezes. N� o 1 a 5 anos. N� o, n� o tenho nenhuma dessas doen� as 2 est� mago raio x, vazio durar, horas pequeno, caf� a meia-noite, comer, nada, fazer caf� da manh� beba, � gua pergunta, o departamento poder compreendo, transmitir, importantes, diagn� stico entendi, altera� � o, dez, ciente que, adaptar, direito, solicitar, telefonando precisar, instru� � es, procurar, Servi� o, esclarecer, d� vidas 13h N� o 27/01/2021 11 Sim Sim Sim N� o
1/22/2021 13:30:51 N� o SIM, aceito participar. Jo 1954 Sim Ensino superior completo (faculdade/universidade). Sul Casado(a) / Vive junto 2 Masculino Sou aposentado(a) / pensionista Sim Sim 110 174 Sim Sim, � s vezes. Sim 1 a 5 anos. N� o, n� o tenho nenhuma dessas doen� as 5 est� mago raio x, vazio durar, horas pequeno, caf� a meia-noite, comer, nada, fazer caf� da manh� beba, � gua pergunta, o departamento poder compreendo, transmitir, importantes, diagn� stico entendi, altera� � o, dez, ciente que, adaptar, direito, solicitar, telefonando precisar, instru�

□ es, procurar, Servi□ o, esclarecer, d□ vidas 13:00 N□ o 28/01/2021 09:00 N□ o N□ o N□ o N□ o
1/22/2021 14:17:15 N□ o SIM, aceito participar. AL 2000 Sim Ensino m□ dio completo (colegial). Sudeste Casado(
a) / Vive junto 2 Feminino N□ o Sim Sim 86 157 N□ o Sim, □ s vezes. N□ o 1 a 5 anos. N□ o, n□ o tenho nenhum
a dessas doen□ as 5 est□ mago raio x, vazio durar, horas pequeno, caf□ a meia-noite, comer, nada, fazer caf□ da
manh□ beba, □ gua pergunta, o departamento poder compreendo, transmitir, importantes, diagn□ stico entendi, alte
ra□ □ o, dez, ciente que, adaptar, direito, solicitar, telefonando precisar, instru□ □ es, procurar, Servi□ o, esclarecer
, d□ vidas 13h N□ o 28/01/2021 11h Sim Sim Sim N□ o
1/22/2021 14:41:04 N□ o SIM, aceito participar. SO 1955 Sim Ensino m□ dio completo (colegial). Sudeste Casado(
a) / Vive junto 1 Feminino N□ o Sim Sim 95 165 N□ o N□ o. Sim Mais de 10 anos. N□ o, n□ o tenho nenhuma des
sas doen□ as 4 est□ mago raio x, vazio durar, horas pequeno, caf□ a meia-noite, comer, nada, fazer caf□ da manh
□ beba, □ gua pergunta, o departamento poder compreendo, transmitir, importantes, diagn□ stico entendi, altera□
□ o, dez, ciente que, adaptar, direito, solicitar, telefonando precisar, instru□ □ es, procurar, Servi□ o, esclarecer, d
□ vidas 13 hs N□ o 28/01/2021 11 hs Sim N□ o N□ o N□ o
1/22/2021 14:43:48 N□ o SIM, aceito participar. HL 1977 Sim Ensino m□ dio completo (colegial). Norte Casado(a)
/ Vive junto 2 Feminino N□ o Sim Sim 110 164 N□ o Sim, □ s vezes. N□ o Menos de 1 ano. N□ o, n□ o tenho ne
nhuma dessas doen□ as 2 est□ mago raio x, vazio durar, horas pequeno, caf□ a meia-noite, comer, nada, fazer caf
□ da manh□ beba, □ gua pergunta, o departamento poder compreendo, transmitir, importantes, diagn□ stico enten
di, altera□ □ o, dez, ciente que, adaptar, direito, solicitar, telefonando precisar, instru□ □ es, procurar, Servi□ o, esc
larecer, d□ vidas 13 Sim 28/01/2021 11 N□ o N□ o Sim Sim
1/22/2021 16:19:29 N□ o SIM, aceito participar. IS 1970 Sim P□ s-Gradua□ □ o (especializa□ □ o, mestrado e/ou
doutorado). Nordeste Separado(a) / Divorciado(a) 5 Feminino Sim Sim Sim 52 160 N□ o N□ o. N□ o 5 a 10 anos.
Diabetes, Outra que n□ o est□ entre as op□ □ es de resposta 3 est□ mago raio x, vazio durar, horas pequeno, caf□
a meia-noite, comer, nada, fazer caf□ da manh□ beba, □ gua pergunta, o departamento poder compreendo, transm
itir, importantes, diagn□ stico entendi, altera□ □ o, dez, ciente que, adaptar, direito, solicitar, telefonando precisar, i
nstru□ □ es, procurar, Servi□ o, esclarecer, d□ vidas 13h N□ o 28/01/2021 11h N□ o N□ o N□ o N□ o
1/22/2021 20:20:01 N□ o SIM, aceito participar. MA 1972 Sim Ensino superior completo (faculdade/universidade).
Sudeste Vi□ vo(a) 2 Feminino Sim Sim Sim 71 155 N□ o N□ o. Sim Menos de 1 ano. Outra que n□ o est□ entre a
s op□ □ es de resposta 1 est□ mago raio x, vazio durar, horas pequeno, caf□ a meia-noite, comer, nada, fazer caf□
da manh□ beba, □ gua pergunta, o departamento poder compreendo, transmitir, importantes, diagn□ stico entendi,
altera□ □ o, dez, ciente que, adaptar, direito, solicitar, telefonando precisar, instru□ □ es, procurar, Servi□ o, esclar
ecer, d□ vidas 13 N□ o 28/01/2021 11 N□ o N□ o N□ o N□ o
1/22/2021 20:46:04 N□ o SIM, aceito participar. Lu 1970 Sim Ensino m□ dio completo (colegial). Sudeste Solteiro(
a) 1 Feminino N□ o Sim Sim 80 163 N□ o Sim, □ s vezes. N□ o Mais de 10 anos. Diabetes 3 est□ mago raio x, vaz
io durar, horas pequeno, caf□ a meia-noite, comer, nada, fazer caf□ da manh□ beba, □ gua pergunta, o departame
nto poder compreendo, transmitir, importantes, diagn□ stico entendi, altera□ □ o, dez, ciente que, adaptar, direito, s
olicitar, telefonando precisar, instru□ □ es, procurar, Servi□ o, esclarecer, d□ vidas 13 N□ o 01/05/2021 11 N□ o
N□ o N□ o N□ o
1/22/2021 21:43:02 N□ o SIM, aceito participar. Fa 79 Sim Ensino fundamental completo. Sul Casado(a) / Vive junt
o 3 Feminino N□ o Sim Sim 120 159 N□ o N□ o. Sim 5 a 10 anos. N□ o, n□ o tenho nenhuma dessas doen□ as 5 e
st□ mago raio x, vazio durar, horas pequeno, caf□ a meia-noite, comer, nada, fazer caf□ da manh□ beba, □ gua p
ergunta, o departamento poder compreendo, transmitir, importantes, diagn□ stico entendi, altera□ □ o, dez, ciente q
ue, adaptar, direito, solicitar, telefonando precisar, instru□ □ es, procurar, Servi□ o, esclarecer, d□ vidas 13:00 N□
o 21/01/2021 11:00 Sim Sim N□ o N□ o
1/22/2021 22:20:59 N□ o SIM, aceito participar. Vi 1979 Sim Ensino m□ dio completo (colegial). Sudeste Casado(
a) / Vive junto 3 Feminino Sou aposentado(a) / pensionista Sim Sim 98.5 160 N□ o Sim, □ s vezes. N□ o Mais de 1
0 anos. Diabetes, Infarto, Outra que n□ o est□ entre as op□ □ es de resposta 6 est□ mago raio x, vazio durar, horas
pequeno, caf□ a meia-noite, comer, nada, fazer caf□ da manh□ beba, □ gua pergunta, o departamento poder comp
reendo, transmitir, importantes, diagn□ stico entendi, altera□ □ o, dez, ciente que, adaptar, direito, solicitar, telefona
ndo precisar, instru□ □ es, procurar, Servi□ o, esclarecer, d□ vidas 13h. N□ o 28/01/2021 11h ou 15h. Sim Sim Si
m Sim
1/22/2021 23:13:03 N□ o SIM, aceito participar. Sh 1979 Sim Ensino m□ dio completo (colegial). Nordeste Casado
(a) / Vive junto 5 Feminino N□ o Sim Sim 66 160 N□ o N□ o. N□ o 1 a 5 anos. N□ o, n□ o tenho nenhuma dessas
doen□ as 0 est□ mago raio x, vazio durar, horas pequeno, caf□ a meia-noite, comer, nada, fazer caf□ da manh□ b
eba, □ gua pergunta, o departamento poder compreendo, transmitir, importantes, diagn□ stico entendi, altera□ □ o,
dez, ciente que, adaptar, direito, solicitar, telefonando precisar, instru□ □ es, procurar, Servi□ o, esclarecer, d□ vida

s 13 horas N� o 25/01/2021 11 N� o Sim Sim Sim
1/23/2021 0:11:22 N� o SIM, aceito participar. SA 19/06/1987 Sim P� s-Gradua� � o (especializa� � o, mestrad
o e/ou doutorado). Sul Solteiro(a) 2 Masculino Sim Sim Sim 90 170 N� o N� o. Sim Menos de 1 ano. N� o, n� o t
enho nenhuma dessas doen� as 0 est� mago raio x, vazio durar, horas pequeno, caf� a meia-noite, comer, nada, fa
zer caf� da manh� beba, � gua pergunta, o departamento poder compreendo, transmitir, importantes, diagn� stico
entendi, altera� � o, dez, ciente que, adaptar, direito, solicitar, telefonando precisar, instru� � es, procurar, Servi
� o, esclarecer, d� vidas 13 N� o 28/01/2021 11 horas N� o N� o N� o N� o
1/23/2021 0:13:40 N� o SIM, aceito participar. AN 1970 Sim Ensino superior completo (faculdade/universidade). S
udeste Solteiro(a) 3 Feminino N� o Sim Sim 75 160 N� o N� o. N� o 5 a 10 anos. Outra que n� o est� entre as o
p� � es de resposta 4 est� mago raio x, vazio durar, horas pequeno, caf� a meia-noite, comer, nada, fazer caf� da
manh� beba, � gua pergunta, o departamento poder compreendo, transmitir, importantes, diagn� stico entendi, alt
era� � o, dez, ciente que, adaptar, direito, solicitar, telefonando precisar, instru� � es, procurar, Servi� o, esclarece
r, d� vidas 15 h Sim 29/01/2021 11h N� o N� o N� o Sim
1/23/2021 0:24:14 N� o SIM, aceito participar. Ro 1963 Sim Ensino superior completo (faculdade/universidade). S
ul Solteiro(a) 3 Feminino N� o Sim Sim 85 163 N� o N� o. N� o 1 a 5 anos. N� o, n� o tenho nenhuma dessas d
oen� as 2 est� mago raio x, vazio durar, horas pequeno, caf� a meia-noite, comer, nada, fazer caf� da manh� be
ba, � gua pergunta, o departamento poder compreendo, transmitir, importantes, diagn� stico entendi, altera� � o, d
ez, ciente que, adaptar, direito, solicitar, telefonando precisar, instru� � es, procurar, Servi� o, esclarecer, d� vidas
19h N� o 28/01/2021 11h N� o N� o N� o N� o
1/23/2021 0:42:19 N� o SIM, aceito participar. Mi 1982 Sim Ensino m� dio completo (colegial). Sudeste Casado(a
) / Vive junto 2 Feminino N� o Sim Sim 100 170 N� o N� o. N� o 5 a 10 anos. Diabetes, Outra que n� o est� en
tre as op� � es de resposta 5 pontos raio x, vazio durar, horas pequeno, caf� a meia-noite, comer, nada, fazer caf�
da manh� beba, � gua pergunta, o departamento poder compreendo, transmitir, importantes, diagn� stico entendi,
altera� � o, dez, ciente que, adaptar, direito, solicitar, telefonando precisar, instru� � es, procurar, Servi� o, esclar
ecer, d� vidas 13 horas N� o 28/01/2021 9 Sim Sim N� o N� o
1/23/2021 0:50:46 N� o SIM, aceito participar. Pa 1966 Sim P� s-Gradua� � o (especializa� � o, mestrado e/ou d
outorado). Sudeste Casado(a) / Vive junto 4 Feminino Sim Sim Sim 76 167 N� o Sim, � s vezes. N� o Mais de 10
anos. Diabetes 2 est� mago raio x, vazio durar, horas pequeno, caf� a meia-noite, comer, nada, fazer caf� da man
h� beba, � gua pergunta, o departamento poder compreendo, transmitir, importantes, diagn� stico entendi, altera�
� o, dez, ciente que, adaptar, direito, solicitar, telefonando precisar, instru� � es, procurar, Servi� o, esclarecer, d
� vidas 13h? N� o 28/01/2021 11h Sim Sim N� o Sim
1/23/2021 7:59:37 N� o SIM, aceito participar. Ro 1972 Sim Ensino m� dio completo (colegial). Sudeste Separado
(a) / Divorciado(a) 3 Feminino Sim Sim Sim 81 170 N� o Sim, � s vezes. Sim Mais de 10 anos. Outra que n� o est
� entre as op� � es de resposta 1 est� mago raio x, vazio durar, horas pequeno, caf� a meia-noite, comer, nada, f
azer caf� da manh� beba, � gua pergunta, o departamento poder compreendo, transmitir, importantes, diagn� stic
o entendi, altera� � o, dez, ciente que, adaptar, direito, solicitar, telefonando precisar, instru� � es, procurar, Servi
� o, esclarecer, d� vidas 13 Sim 28/01/2021 1130 N� o N� o N� o Sim
1/23/2021 12:40:24 N� o SIM, aceito participar. So 1965 Sim Ensino superior completo (faculdade/universidade). S
ul Separado(a) / Divorciado(a) 2 Feminino Sou aposentado(a) / pensionista Sim Sim 98 170 N� o N� o. Sim Mais d
e 10 anos. Diabetes 4 pontos raio x, vazio durar, horas pequeno, caf� a meia-noite, comer, nada, fazer caf� da man
h� beba, � gua pergunta, o departamento poder compreendo, transmitir, importantes, diagn� stico entendi, altera�
� o, dez, ciente que, adaptar, direito, solicitar, telefonando precisar, instru� � es, procurar, Servi� o, esclarecer, d
� vidas 13 horas N� o 28/01/2021 11 horas N� o N� o N� o N� o
1/23/2021 13:48:46 N� o SIM, aceito participar. MA 1980 Sim P� s-Gradua� � o (especializa� � o, mestrado e/o
u doutorado). Nordeste Solteiro(a) 2 Masculino Sim Sim Sim 103 175 N� o Sim, � s vezes. Sim 5 a 10 anos. Diabet
es, Doen� a arterial coronariana (fiz cateterismo nos vasos do cora� � o) 7 pontos raio x, vazio durar, horas pequen
o, caf� a meia-noite, comer, nada, fazer caf� da manh� beba, � gua pergunta, o departamento poder compreendo
, transmitir, importantes, diagn� stico entendi, altera� � o, dez, ciente assim, alimentar, brilho, reciclar, contando pr
ecisar, instru� � es, procurar, Servi� o, esclarecer, d� vidas 13 N� o 28/01/2021 11 Sim Sim N� o Sim
1/23/2021 14:06:42 N� o SIM, aceito participar. Ad 1971 Sim P� s-Gradua� � o (especializa� � o, mestrado e/ou
doutorado). Norte Separado(a) / Divorciado(a) 2 Feminino N� o Sim Sim 76 157 N� o Sim, � s vezes. Sim 5 a 10
anos. N� o, n� o tenho nenhuma dessas doen� as 4 est� mago raio x, vazio durar, horas pequeno, caf� a meia-noi
te, comer, nada, fazer caf� da manh� beba, � gua pergunta, o departamento poder compreendo, transmitir, import
antes, diagn� stico entendi, altera� � o, dez, ciente que, adaptar, direito, solicitar, telefonando precisar, instru� � e
s, procurar, Servi� o, esclarecer, d� vidas 13 N� o 28/01/2021 11 N� o N� o N� o N� o

1/23/2021 14:19:41 N� o SIM, aceito participar. LG 1979 Sim P� s-Gradua� � o (especializa� � o, mestrado e/ou doutorado). Centro-Oeste Casado(a) / Vive junto 4 Feminino Sim Sim Sim 67 157 N� o N� o. N� o 1 a 5 anos. N� o, n� o tenho nenhuma dessas doen� as 3 est� mago raio x, vazio durar, horas pequeno, caf� a meia-noite, comer, nada, fazer caf� da manh� beba, � gua pergunta, o departamento poder compreendo, transmitir, importantes, diagn� stico entendi, altera� � o, dez, ciente que, adaptar, direito, solicitar, telefonando precisar, instru� � es, procurar, Servi� o, esclarecer, d� vidas 13 N� o 28/01/2021 11 N� o N� o N� o N� o

1/23/2021 14:54:34 N� o SIM, aceito participar. El 1957 Sim P� s-Gradua� � o (especializa� � o, mestrado e/ou doutorado). Nordeste Solteiro(a) 05 Feminino Sou aposentado(a) / pensionista Sim Sim 67 161 N� o N� o. Sim Mais de 10 anos. Diabetes, Doen� a nos rins 10 est� mago raio x, vazio durar, horas pequeno, caf� a meia-noite, comer, nada, fazer caf� da manh� beba, � gua pergunta, o departamento poder compreendo, transmitir, importantes, diagn� stico entendi, altera� � o, dez, ciente que, adaptar, direito, solicitar, telefonando precisar, instru� � es, procurar, Servi� o, esclarecer, d� vidas 13 N� o 28/01/2021 11 N� o N� o N� o N� o

1/23/2021 16:49:59 N� o SIM, aceito participar. S� 1956 Sim Ensino superior completo (faculdade/universidade). Sudeste Casado(a) / Vive junto 2 Feminino Sou aposentado(a) / pensionista Sim Sim 59 154 N� o Sim, � s vezes. N� o Mais de 10 anos. N� o, n� o tenho nenhuma dessas doen� as 5 pontos raio x, vazio durar, horas pequeno, caf� a meia-noite, comer, nada, fazer caf� da manh� beba, � gua pergunta, o departamento poder compreendo, transmitir, importantes, diagn� stico entendi, altera� � o, dez, ciente que, adaptar, direito, solicitar, telefonando precisar, instru� � es, procurar, Servi� o, esclarecer, d� vidas 13:00 horas N� o 28/01/2021 11:00 hs N� o N� o N� o N� o

1/23/2021 17:28:18 N� o SIM, aceito participar. CS 1975 Sim Ensino m� dio completo (colegial). Sul Solteiro(a) 2 Feminino N� o Sim Sim 66 160 N� o N� o. N� o Menos de 1 ano. N� o, n� o tenho nenhuma dessas doen� as 3 est� mago raio x, vazio durar, horas pequeno, caf� a meia-noite, comer, nada, fazer caf� da manh� beba, � gua pergunta, o departamento poder compreendo, transmitir, importantes, diagn� stico entendi, altera� � o, dez, ciente que, adaptar, direito, solicitar, telefonando precisar, instru� � es, procurar, Servi� o, esclarecer, d� vidas 19 hs N� o 28/01/2021 9hs N� o N� o N� o Sim

1/23/2021 17:37:19 N� o SIM, aceito participar. Ad 1968 Sim Ensino superior completo (faculdade/universidade). Centro-Oeste Solteiro(a) 3 Feminino N� o Sim Sim 93 160 N� o Sim, � s vezes. Sim Mais de 10 anos. N� o, n� o tenho nenhuma dessas doen� as 6 est� mago raio x, vazio durar, horas pequeno, caf� a meia-noite, comer, nada, fazer caf� da manh� beba, � gua pergunta, o departamento poder compreendo, transmitir, importantes, diagn� stico entendi, altera� � o, dez, ciente que, adaptar, direito, solicitar, telefonando precisar, instru� � es, procurar, Servi� o, esclarecer, d� vidas 13 N� o 28/01/2021 11 N� o Sim N� o N� o

1/23/2021 18:34:13 N� o SIM, aceito participar. RU 1976 Sim Ensino m� dio completo (colegial). Sudeste Casado(a) / Vive junto 4 Feminino N� o Sim Sim 59 159 N� o Sim, � s vezes. N� o 5 a 10 anos. N� o, n� o tenho nenhuma dessas doen� as 1 est� mago raio x, vazio durar, horas pequeno, caf� a meia-noite, comer, nada, fazer caf� da manh� beba, � gua pergunta, o departamento poder compreendo, transmitir, importantes, diagn� stico entendi, altera� � o, dez, ciente que, adaptar, direito, solicitar, telefonando precisar, instru� � es, procurar, Servi� o, esclarecer, d� vidas 13 N� o 28/01/2021 11 Sim Sim N� o N� o

1/23/2021 19:58:03 N� o SIM, aceito participar. Cl 1969 Sim Ensino m� dio completo (colegial). Sudeste Solteiro(a) 2 Feminino N� o Sim Sim 65 162 Sim Sim, � s vezes. N� o 5 a 10 anos. N� o, n� o tenho nenhuma dessas doen� as 2 est� mago raio x, vazio durar, horas pequeno, caf� a meia-noite, comer, nada, fazer caf� da manh� beba, � gua pergunta, o departamento poder compreendo, transmitir, importantes, diagn� stico entendi, altera� � o, dez, ciente que, adaptar, direito, solicitar, telefonando precisar, instru� � es, procurar, Servi� o, esclarecer, d� vidas 13 Sim 28/01/2021 11 N� o N� o Sim Sim

1/23/2021 20:05:02 N� o SIM, aceito participar. Ar 1981 Sim Ensino superior completo (faculdade/universidade). Sul Casado(a) / Vive junto 4 Feminino N� o Sim Sim 70 167 N� o Sim, � s vezes. N� o Menos de 1 ano. N� o, n� o tenho nenhuma dessas doen� as 2 est� mago raio x, vazio durar, horas pequeno, caf� a meia-noite, comer, nada, fazer caf� da manh� beba, � gua pergunta, o departamento poder compreendo, transmitir, importantes, diagn� stico entendi, altera� � o, dez, ciente que, adaptar, direito, solicitar, telefonando precisar, instru� � es, procurar, Servi� o, esclarecer, d� vidas 13horas N� o 28/01/2021 11:30 N� o N� o N� o N� o

1/23/2021 21:49:26 N� o SIM, aceito participar. Iv 1978 Sim Ensino m� dio completo (colegial). Nordeste Casado(a) / Vive junto 2 Feminino Sim Sim Sim 100 158 N� o Sim, � s vezes. Sim 5 a 10 anos. Outra que n� o est� entre as op� � es de resposta 6 est� mago raio x, vazio durar, horas pequeno, caf� a meia-noite, comer, nada, fazer caf� da manh� beba, � gua pergunta, o departamento poder compreendo, transmitir, importantes, diagn� stico entendi, altera� � o, dez, ciente que, adaptar, direito, solicitar, telefonando precisar, instru� � es, procurar, Servi� o, esclarecer, d� vidas 13:00 hrs N� o 28/01/2021 11:00 hrs N� o Sim N� o N� o

1/23/2021 22:38:20 N□ o SIM, aceito participar. Si 13/10/1968 Sim Ensino m□ dio completo (colegial). Sul Casado
(a) / Vive junto 2 Feminino Sim Sim Sim 82 156 N□ o N□ o. N□ o 5 a 10 anos. Diabetes 4 est□ mago raio x, vazio
durar, horas pequeno, caf□ a meia-noite, comer, nada, fazer caf□ da manh□ beba, □ gua pergunta, o departament
o poder compreendo, transmitir, importantes, diagn□ stico entendi, altera□ □ o, dez, ciente que, adaptar, direito, soli
citar, telefonando precisar, instru□ □ es, procurar, Servi□ o, esclarecer, d□ vidas 13 N□ o 21/01/2021 11 N□ o N□
o N□ o N□ o
1/23/2021 23:10:35 N□ o SIM, aceito participar. SI 1961 Sim Ensino superior completo (faculdade/universidade). C
entro-Oeste Solteiro(a) 2 Feminino Sou aposentado(a) / pensionista Sim Sim 69 170 N□ o N□ o. Sim 1 a 5 anos. Ou
tra que n□ o est□ entre as op□ □ es de resposta 3 est□ mago raio x, vazio durar, horas pequeno, caf□ a meia-noite
, comer, nada, fazer caf□ da manh□ beba, □ gua pergunta, o departamento poder compreendo, transmitir, importan
tes, diagn□ stico entendi, altera□ □ o, dez, ciente que, adaptar, direito, solicitar, telefonando precisar, instru□ □ es,
procurar, Servi□ o, esclarecer, d□ vidas 13 horas N□ o 28/01/2021 11 horas Sim N□ o N□ o N□ o
1/24/2021 0:05:50 N□ o SIM, aceito participar. An 1979 Sim Ensino m□ dio completo (colegial). Nordeste Separad
o(a) / Divorciado(a) 5 Feminino Sim Sim Sim 85 165 N□ o Sim, □ s vezes. N□ o 1 a 5 anos. N□ o, n□ o tenho nen
huma dessas doen□ as 1 est□ mago raio x, vazio durar, horas pequeno, caf□ a meia-noite, comer, nada, fazer caf□
da manh□ beba, □ gua pergunta, o departamento poder compreendo, transmitir, importantes, diagn□ stico entendi,
altera□ □ o, dez, ciente que, adaptar, direito, solicitar, telefonando precisar, instru□ □ es, procurar, Servi□ o, esclar
ecer, d□ vidas 13 horas Sim 28/01/2021 11h N□ o N□ o N□ o N□ o
1/24/2021 4:35:13 N□ o SIM, aceito participar. Sa 1973 Sim Ensino m□ dio completo (colegial). Sudeste Casado(a)
/ Vive junto 5 Feminino N□ o Sim Sim 84 168 Sim N□ o. N□ o 5 a 10 anos. N□ o, n□ o tenho nenhuma dessas do
en□ as 2 est□ mago raio x, vazio durar, horas pequeno, caf□ a meia-noite, comer, nada, fazer caf□ da manh□ beb
a, □ gua pergunta, o departamento poder compreendo, transmitir, importantes, diagn□ stico entendi, altera□ □ o, de
z, ciente que, adaptar, direito, solicitar, telefonando precisar, instru□ □ es, procurar, Servi□ o, esclarecer, d□ vidas
13 N□ o 28/01/2021 11 N□ o N□ o N□ o N□ o
1/24/2021 9:38:37 N□ o SIM, aceito participar. Mr 1969 Sim Ensino fundamental completo. Centro-Oeste Casado(a
) / Vive junto 1 Masculino Sim Sim Sim 93 170 Sim Sim, frequentemente. N□ o Menos de 1 ano. AVC (derrame) 1
7 pontos raio x, vazio durar, horas pequeno, caf□ a meia-noite, comer, nada, fazer caf□ da manh□ beba, □ gua pe
rgunta, o departamento poder compreendo, transmitir, importantes, diagn□ stico entendi, altera□ □ o, dez, ciente qu
e, adaptar, direito, solicitar, telefonando precisar, instru□ □ es, procurar, Servi□ o, esclarecer, d□ vidas 13h N□ o 1
3/02/2021 11 Sim Sim N□ o N□ o
1/24/2021 10:05:30 N□ o SIM, aceito participar. Re 1962 Sim Ensino m□ dio completo (colegial). Nordeste Solteir
o(a) 3 Feminino N□ o Sim Sim 106 173 N□ o N□ o. N□ o Mais de 10 anos. N□ o, n□ o tenho nenhuma dessas doe
n□ as 5 pontos raio x, vazio durar, horas pequeno, caf□ a meia-noite, comer, nada, fazer caf□ da manh□ beba, □
gua pergunta, o departamento poder compreendo, transmitir, importantes, diagn□ stico entendi, altera□ □ o, dez, cie
nte que, adaptar, direito, solicitar, telefonando precisar, instru□ □ es, procurar, Servi□ o, esclarecer, d□ vidas 13 N
□ o 24/07/2021 11 N□ o N□ o N□ o N□ o
1/24/2021 11:16:38 N□ o SIM, aceito participar. Ro 1966 Sim Ensino superior completo (faculdade/universidade).
Sul Casado(a) / Vive junto 2 Feminino Sou aposentado(a) / pensionista Sim Sim 88 149 N□ o Sim, □ s vezes. Sim
Mais de 10 anos. Outra que n□ o est□ entre as op□ □ es de resposta 3 est□ mago raio x, vazio durar, horas pequen
o, caf□ a meia-noite, comer, nada, fazer caf□ da manh□ beba, □ gua pergunta, o departamento poder compreendo
, transmitir, importantes, diagn□ stico entendi, altera□ □ o, dez, ciente que, adaptar, direito, solicitar, telefonando pr
ecisar, instru□ □ es, procurar, Servi□ o, esclarecer, d□ vidas 13 N□ o 28/01/2021 11 N□ o N□ o N□ o N□ o
1/24/2021 11:36:33 N□ o SIM, aceito participar. Si 1972 Sim Ensino fundamental completo. Sul Casado(a) / Vive j
unto 3 Feminino N□ o Sim Sim 130 160 N□ o Sim, □ s vezes. N□ o Menos de 1 ano. Diabetes, Outra que n□ o est
□ entre as op□ □ es de resposta 3 est□ mago raio x, vazio durar, horas pequeno, caf□ a meia-noite, comer, nada, f
azer caf□ da manh□ beba, □ gua pergunta, o departamento poder compreendo, transmitir, importantes, diagn□ stic
o entendi, altera□ □ o, dez, ciente que, adaptar, direito, solicitar, telefonando precisar, instru□ □ es, procurar, Servi
□ o, esclarecer, d□ vidas 13 hs N□ o 28/01/2021 11 hs Sim Sim Sim Sim
1/24/2021 12:47:02 N□ o SIM, aceito participar. Ma 1963 Sim Ensino m□ dio completo (colegial). Sudeste Separad
o(a) / Divorciado(a) 2 Masculino Sim Sim Sim 90 180 Sim Sim, □ s vezes. N□ o Mais de 10 anos. N□ o, n□ o tenh
o nenhuma dessas doen□ as 01 est□ mago raio x, vazio durar, horas pequeno, caf□ a meia-noite, comer, nada, fazer
caf□ da manh□ beba, □ gua pergunta, o departamento poder compreendo, transmitir, importantes, diagn□ stico en
tendi, altera□ □ o, dez, ciente que, adaptar, direito, solicitar, telefonando precisar, instru□ □ es, procurar, Servi□ o,
esclarecer, d□ vidas 13 N□ o 28/01/2021 11 N□ o N□ o N□ o N□ o
1/24/2021 14:47:26 N□ o SIM, aceito participar. cr 1968 Sim Ensino superior completo (faculdade/universidade). S

udeste Casado(a) / Vive junto 2 Feminino Sim Sim Sim 64 160 N□ o N□ o. N□ o 5 a 10 anos. N□ o, n□ o tenho ne
nhuma dessas doen□ as 2 est□ mago raio x, vazio durar, horas pequeno, caf□ a meia-noite, comer, nada, fazer caf
□ da manh□ beba, □ gua pergunta, o departamento poder compreendo, transmitir, importantes, diagn□ stico enten
di, altera□ □ o, dez, ciente que, adaptar, direito, solicitar, telefonando precisar, instru□ □ es, procurar, Servi□ o, esc
larecer, d□ vidas 13 horas N□ o 27/01/2021 11 horas N□ o Sim N□ o N□ o
1/24/2021 15:12:52 N□ o SIM, aceito participar. JA 1979 Sim Ensino m□ dio completo (colegial). Sudeste Casado(
a) / Vive junto 3 Feminino Sim Sim Sim 89 158 N□ o N□ o. N□ o Mais de 10 anos. N□ o, n□ o tenho nenhuma des
sas doen□ as 3 est□ mago raio x, vazio durar, horas pequeno, caf□ a meia-noite, comer, nada, fazer caf□ da manh
□ beba, □ gua pergunta, o departamento poder compreendo, transmitir, importantes, diagn□ stico entendi, altera□
□ o, dez, ciente que, adaptar, direito, solicitar, telefonando precisar, instru□ □ es, procurar, Servi□ o, esclarecer, d
□ vidas 13 horas N□ o 28/01/2021 11 horas N□ o N□ o N□ o N□ o
1/24/2021 15:47:36 N□ o SIM, aceito participar. SI 1966 Sim Ensino m□ dio completo (colegial). Centro-Oeste Cas
ado(a) / Vive junto 1 Feminino N□ o Sim Sim 54 151 N□ o Sim, □ s vezes. N□ o 5 a 10 anos. N□ o, n□ o tenho ne
nhuma dessas doen□ as 5 est□ mago raio x, vazio durar, horas pequeno, caf□ a meia-noite, comer, nada, fazer caf
□ da manh□ beba, □ gua pergunta, o departamento poder compreendo, transmitir, importantes, diagn□ stico enten
di, altera□ □ o, dez, ciente que, adaptar, direito, solicitar, telefonando precisar, instru□ □ es, procurar, Servi□ o, esc
larecer, d□ vidas 13h N□ o 28/01/2021 11h Sim Sim N□ o Sim
1/24/2021 18:55:10 N□ o SIM, aceito participar. Fa 1976 Sim Ensino m□ dio completo (colegial). Sudeste Casado(
a) / Vive junto 3 Feminino Sim Sim Sim 100 166 N□ o N□ o. N□ o 1 a 5 anos. N□ o, n□ o tenho nenhuma dessas d
oen□ as 1 est□ mago raio x, vazio durar, horas pequeno, caf□ a meia-noite, comer, nada, fazer caf□ da manh□ be
ba, □ gua pergunta, o departamento poder compreendo, transmitir, importantes, diagn□ stico entendi, altera□ □ o, d
ez, ciente que, adaptar, direito, solicitar, telefonando precisar, instru□ □ es, procurar, Servi□ o, esclarecer, d□ vidas
13 N□ o 28/01/2021 11 N□ o N□ o N□ o N□ o
1/24/2021 18:59:29 N□ o SIM, aceito participar. Mo 1973 Sim Ensino superior completo (faculdade/universidade).
Sudeste Casado(a) / Vive junto 4 Feminino Sim Sim Sim 73 163 N□ o N□ o. N□ o Mais de 10 anos. N□ o, n□ o te
nho nenhuma dessas doen□ as 3 est□ mago raio x, vazio durar, horas pequeno, caf□ a meia-noite, comer, nada, faz
er caf□ da manh□ beba, □ gua pergunta, o departamento poder compreendo, transmitir, importantes, diagn□ stico
entendi, altera□ □ o, dez, ciente que, adaptar, direito, solicitar, telefonando precisar, instru□ □ es, procurar, Servi□
o, esclarecer, d□ vidas 13 horas N□ o 28/01/2021 As 11 horas N□ o N□ o N□ o N□ o
1/24/2021 19:32:02 N□ o SIM, aceito participar. An 1971 Sim Ensino m□ dio completo (colegial). Nordeste Solteir
o(a) 3 Feminino N□ o Sim Sim 57 148 N□ o Sim, □ s vezes. Sim Mais de 10 anos. N□ o, n□ o tenho nenhuma dess
as doen□ as 5 est□ mago raio x, vazio durar, horas pequeno, caf□ a meia-noite, comer, nada, fazer caf□ da manh
□ beba, □ gua pergunta, o departamento poder compreendo, transmitir, importantes, diagn□ stico entendi, altera□
□ o, dez, ciente que, adaptar, direito, solicitar, telefonando precisar, instru□ □ es, procurar, Servi□ o, esclarecer, d
□ vidas 13 Sim 28/01/2021 11 Sim Sim Sim Sim
1/24/2021 20:39:31 N□ o SIM, aceito participar. FS 1980 Sim P□ s-Gradua□ □ o (especializa□ □ o, mestrado e/ou
doutorado). Nordeste Casado(a) / Vive junto 2 Feminino Sim Sim Sim 98 163 N□ o Sim, □ s vezes. Sim 5 a 10 ano
s. Diabetes 7 est□ mago raio x, vazio durar, horas pequeno, caf□ a meia-noite, comer, nada, fazer caf□ da manh□
beba, □ gua pergunta, o departamento poder compreendo, transmitir, importantes, diagn□ stico entendi, altera□ □ o,
dez, ciente que, adaptar, direito, solicitar, telefonando precisar, instru□ □ es, procurar, Servi□ o, esclarecer, d□ vid
as 13 N□ o 28/01/2021 11 N□ o N□ o N□ o N□ o
1/24/2021 20:49:02 N□ o SIM, aceito participar. Fe 1971 Sim Ensino m□ dio completo (colegial). Sudeste Separad
o(a) / Divorciado(a) 4 Feminino Sim Sim Sim 89 164 N□ o Sim, □ s vezes. N□ o 1 a 5 anos. Diabetes, Outra que n
□ o est□ entre as op□ □ es de resposta 5 est□ mago raio x, vazio durar, horas pequeno, caf□ a meia-noite, comer,
nada, fazer caf□ da manh□ beba, □ gua pergunta, o departamento poder compreendo, transmitir, importantes, diag
n□ stico entendi, altera□ □ o, dez, ciente que, adaptar, direito, solicitar, telefonando precisar, instru□ □ es, procurar
, Servi□ o, esclarecer, d□ vidas 13hs N□ o 28/01/2021 11hs Sim Sim Sim N□ o
1/24/2021 21:02:40 N□ o SIM, aceito participar. Si 1970 Sim Ensino m□ dio completo (colegial). Sudeste Casado(a
) / Vive junto 3 Feminino Sim Sim Sim 79 161 N□ o Sim, □ s vezes. N□ o 1 a 5 anos. N□ o, n□ o tenho nenhuma d
essas doen□ as 3 est□ mago raio x, vazio durar, horas pequeno, caf□ a meia-noite, comer, nada, fazer caf□ da man
h□ beba, □ gua pergunta, o departamento poder compreendo, transmitir, importantes, diagn□ stico entendi, altera□
□ o, dez, ciente que, adaptar, direito, solicitar, telefonando precisar, instru□ □ es, procurar, Servi□ o, esclarecer, d
□ vidas 13 N□ o 28/01/2021 11 Sim Sim N□ o Sim
1/24/2021 21:13:48 N□ o SIM, aceito participar. Ro 1963 Sim Ensino superior completo (faculdade/universidade).
Nordeste Solteiro(a) 2 Feminino Sou aposentado(a) / pensionista Sim Sim 75 156 N□ o N□ o. N□ o Mais de 10 ano

s. N□ o, n□ o tenho nenhuma dessas doen□ as 1 est□ mago raio x, vazio durar, horas pequeno, caf□ a meia-noite, comer, nada, fazer caf□ da manh□ beba, □ gua pergunta, o departamento poder compreendo, transmitir, important es, diagn□ stico entendi, altera□ □ o, dez, ciente que, adaptar, direito, solicitar, telefonando precisar, instru□ □ es, p rocurar, Servi□ o, esclarecer, d□ vidas 13 N□ o 28/01/2021 11 N□ o N□ o N□ o Sim
1/24/2021 21:21:57 N□ o SIM, aceito participar. Ms 78 Sim Ensino m□ dio completo (colegial). Sul Solteiro(a) 3 F eminino N□ o Sim Sim 120 159 N□ o N□ o. N□ o 5 a 10 anos. Diabetes, Outra que n□ o est□ entre as op□ □ es d e resposta 14 est□ mago raio x, vazio durar, horas pequeno, caf□ a meia-noite, comer, nada, fazer cl□ nica beba, □ gua pergunta, o departamento poder compreendo, transmitir, importantes, diagn□ stico entendi, altera□ □ o, dez, cie nte que, adaptar, direito, solicitar, telefonando precisar, instru□ □ es, procurar, Servi□ o, esclarecer, d□ vidas 19hs Sim 28/02/2021 11h30 N□ o N□ o N□ o N□ o
1/24/2021 23:26:31 N□ o SIM, aceito participar. Ma 1962 Sim Ensino m□ dio completo (colegial). Sudeste Casado(a) / Vive junto 3 Feminino N□ o Sim Sim 85 159 N□ o N□ o. N□ o 5 a 10 anos. N□ o, n□ o tenho nenhuma dessas doen□ as 3 est□ mago raio x, vazio durar, horas pequeno, caf□ a meia-noite, comer, nada, fazer caf□ da manh□ beba, □ gua pergunta, o departamento poder compreendo, transmitir, importantes, diagn□ stico entendi, altera□ □ o, dez, ciente que, adaptar, direito, solicitar, telefonando precisar, instru□ □ es, procurar, Servi□ o, esclarecer, d□ vid as 13 hrs N□ o 24/01/2021 11hrs Sim Sim N□ o N□ o
1/25/2021 1:12:09 N□ o SIM, aceito participar. Sh 1966 Sim P□ s-Gradua□ □ o (especializa□ □ o, mestrado e/ou d outorado). Sudeste Separado(a) / Divorciado(a) 2 Feminino N□ o Sim Sim 82 172 N□ o N□ o. N□ o Mais de 10 an os. N□ o, n□ o tenho nenhuma dessas doen□ as 4 est□ mago raio x, vazio durar, horas pequeno, caf□ a meia-noite, comer, nada, fazer caf□ da manh□ beba, □ gua pergunta, o departamento poder compreendo, transmitir, important es, diagn□ stico entendi, altera□ □ o, dez, ciente que, adaptar, direito, solicitar, telefonando precisar, instru□ □ es, p rocurar, Servi□ o, esclarecer, d□ vidas 13 horas N□ o 28/01/2021 11 horas N□ o N□ o N□ o N□ o
1/25/2021 6:04:23 N□ o SIM, aceito participar. He 1967 Sim P□ s-Gradua□ □ o (especializa□ □ o, mestrado e/ou doutorado). Norte Casado(a) / Vive junto 2 Feminino Sim Sim Sim 60 158 N□ o N□ o. N□ o 1 a 5 anos. N□ o, n□ o tenho nenhuma dessas doen□ as 1 est□ mago raio x, vazio durar, horas pequeno, caf□ a meia-noite, comer, nada, fazer caf□ da manh□ beba, □ gua pergunta, o departamento poder compreendo, transmitir, importantes, diagn□ sti co entendi, altera□ □ o, dez, ciente que, adaptar, direito, solicitar, telefonando precisar, instru□ □ es, procurar, Servi □ o, esclarecer, d□ vidas 13 horas N□ o 28/01/2021 11 horas N□ o N□ o N□ o N□ o
1/25/2021 7:33:57 N□ o SIM, aceito participar. Ag 1934 Sim Ensino fundamental completo. Nordeste Casado(a) / V ive junto 5 Masculino Sou aposentado(a) / pensionista Sim Sim 74 168 N□ o N□ o. N□ o 5 a 10 anos. N□ o, n□ o t enho nenhuma dessas doen□ as 3 est□ mago raio x, vazio durar, horas pequeno, caf□ a meia-noite, comer, nada, fa zer caf□ da manh□ beba, □ gua pergunta, o departamento poder compreendo, transmitir, importantes, diagn□ stico entendi, altera□ □ o, dez, ciente que, adaptar, direito, solicitar, telefonando precisar, instru□ □ es, procurar, Servi □ o, esclarecer, d□ vidas 13 hrs N□ o 28/01/2021 11 hrs N□ o N□ o N□ o N□ o
1/25/2021 7:42:35 N□ o SIM, aceito participar. Is 1978 Sim Ensino superior completo (faculdade/universidade). Ce ntro-Oeste Casado(a) / Vive junto 5 Feminino N□ o Sim Sim 63 150 N□ o N□ o. Sim Menos de 1 ano. N□ o, n□ o tenho nenhuma dessas doen□ as 4 est□ mago raio x, vazio durar, horas pequeno, caf□ a meia-noite, comer, nada, fa zer caf□ da manh□ beba, □ gua pergunta, o departamento poder compreendo, transmitir, importantes, diagn□ stico entendi, altera□ □ o, dez, ciente que, adaptar, direito, solicitar, telefonando precisar, instru□ □ es, procurar, Servi □ o, esclarecer, d□ vidas 13h Sim 28/01/2021 11h N□ o N□ o N□ o N□ o
1/25/2021 8:28:19 N□ o SIM, aceito participar. Ma 1963 Sim Ensino m□ dio completo (colegial). Sudeste Separado (a) / Divorciado(a) 1 Feminino N□ o Sim Sim 59 152 N□ o Sim, □ s vezes. N□ o 1 a 5 anos. N□ o, n□ o tenho nen huma dessas doen□ as 1 est□ mago raio x, vazio durar, horas pequeno, caf□ a meia-noite, comer, nada, fazer caf□ da manh□ beba, □ gua pergunta, o departamento poder compreendo, transmitir, importantes, diagn□ stico entendi, altera□ □ o, dez, ciente que, adaptar, direito, solicitar, telefonando precisar, instru□ □ es, procurar, Servi□ o, esclar ecer, d□ vidas 13hs N□ o 28/01/2021 11hs Sim Sim Sim Sim
1/25/2021 14:24:21 N□ o SIM, aceito participar. RI 1965 Sim P□ s-Gradua□ □ o (especializa□ □ o, mestrado e/ou doutorado). Nordeste Casado(a) / Vive junto 3 Feminino Sim Sim Sim 69 160 N□ o Sim, □ s vezes. Sim Menos de 1 ano. N□ o, n□ o tenho nenhuma dessas doen□ as 1 est□ mago raio x, vazio durar, horas pequeno, caf□ a meia-no ite, comer, nada, fazer caf□ da manh□ beba, □ gua pergunta, o departamento poder compreendo, transmitir, import antes, diagn□ stico entendi, altera□ □ o, dez, ciente que, adaptar, direito, solicitar, telefonando precisar, instru□ □ e s, procurar, Servi□ o, esclarecer, d□ vidas 13 N□ o 28/01/2021 11 N□ o N□ o N□ o Sim
1/25/2021 15:05:26 N□ o SIM, aceito participar. Ma 1962 Sim Ensino superior completo (faculdade/universidade). Sudeste Casado(a) / Vive junto 3 Feminino Sou aposentado(a) / pensionista Sim Sim 87 175 N□ o Sim, □ s vezes. S im 1 a 5 anos. N□ o, n□ o tenho nenhuma dessas doen□ as 4 est□ mago raio x, vazio durar, horas pequeno, caf□ a

meia-noite, comer, nada, fazer caf□ da manh□ beba, □ gua pergunta, o departamento poder compreendo, transmiti
r, importantes, diagn□ stico entendi, altera□ □ o, dez, ciente que, adaptar, direito, solicitar, telefonando precisar, ins
tru□ □ es, procurar, Servi□ o, esclarecer, d□ vidas 13 N□ o 28/01/2021 11 N□ o Sim N□ o N□ o
1/25/2021 15:37:35 N□ o SIM, aceito participar. An 1975 Sim Ensino m□ dio completo (colegial). Sul Casado(a) /
Vive junto 3 Feminino Sim Sim Sim 107 162 N□ o N□ o. N□ o 5 a 10 anos. N□ o, n□ o tenho nenhuma dessas doe
n□ as 4 est□ mago raio x, vazio durar, horas pequeno, caf□ a meia-noite, comer, nada, fazer caf□ da manh□ beba
, □ gua pergunta, o departamento poder compreendo, transmitir, importantes, diagn□ stico entendi, altera□ □ o, dez,
ciente que, adaptar, direito, solicitar, telefonando precisar, instru□ □ es, procurar, Servi□ o, esclarecer, d□ vidas 13
hrs N□ o 28/01/2021 11hrs N□ o N□ o N□ o N□ o
1/25/2021 18:33:51 N□ o SIM, aceito participar. Ve 1969 Sim Ensino m□ dio completo (colegial). Sudeste Casado(
a) / Vive junto 6 Feminino Sim Sim Sim 103 158 N□ o N□ o. N□ o Mais de 10 anos. Diabetes, Doen□ a nos rins,
Outra que n□ o est□ entre as op□ □ es de resposta 7 est□ mago raio x, vazio durar, horas pequeno, caf□ a meia-n
oite, comer, nada, fazer caf□ da manh□ beba, □ gua pergunta, o departamento poder compreendo, transmitir, impo
rtantes, diagn□ stico entendi, altera□ □ o, dez, ciente que, adaptar, direito, solicitar, telefonando precisar, instru□ □
es, procurar, Servi□ o, esclarecer, d□ vidas 13 N□ o 28/01/2021 11 Sim Sim N□ o Sim
1/25/2021 19:07:22 N□ o SIM, aceito participar. Cl 1970 Sim Ensino superior completo (faculdade/universidade). S
udeste Separado(a) / Divorciado(a) 4 Feminino Sou aposentado(a) / pensionista Sim Sim 74 160 N□ o N□ o. Sim M
ais de 10 anos. Diabetes, Outra que n□ o est□ entre as op□ □ es de resposta 6 est□ mago raio x, vazio durar, horas
pequeno, caf□ a meia-noite, comer, nada, fazer caf□ da manh□ beba, □ gua pergunta, o departamento poder comp
reendo, transmitir, importantes, diagn□ stico entendi, altera□ □ o, dez, ciente que, adaptar, direito, solicitar, telefona
ndo precisar, instru□ □ es, procurar, Servi□ o, esclarecer, d□ vidas 13 hs N□ o 17/09/2020 11hs N□ o Sim N□ o N
□ o
1/25/2021 20:21:20 N□ o SIM, aceito participar. Ma 1954 Sim Ensino superior completo (faculdade/universidade).
Nordeste Vi□ vo(a) 4 Feminino Sou aposentado(a) / pensionista Sim Sim 96 164 N□ o N□ o. N□ o Mais de 10 ano
s. Outra que n□ o est□ entre as op□ □ es de resposta 3 est□ mago raio x, vazio durar, horas pequeno, caf□ a meia-
noite, comer, nada, fazer caf□ da manh□ beba, □ gua pergunta, o departamento poder compreendo, transmitir, imp
ortantes, diagn□ stico entendi, altera□ □ o, dez, ciente que, adaptar, direito, solicitar, telefonando precisar, instru□
□ es, procurar, Servi□ o, esclarecer, d□ vidas 13 horas N□ o 28/01/2021 11 horas N□ o N□ o N□ o Sim
1/25/2021 21:03:15 N□ o SIM, aceito participar. Ve 1960 Sim Ensino m□ dio completo (colegial). Nordeste Casado
(a) / Vive junto 5 Feminino N□ o Sim Sim 65 150 N□ o N□ o. Sim 5 a 10 anos. Outra que n□ o est□ entre as op□
□ es de resposta 4 est□ mago raio x, vazio durar, horas pequeno, caf□ a meia-noite, comer, nada, fazer caf□ da ma
nh□ beba, □ gua pergunta, o departamento poder compreendo, transmitir, importantes, diagn□ stico entendi, altera
□ □ o, dez, ciente que, adaptar, direito, solicitar, telefonando precisar, instru□ □ es, procurar, Servi□ o, esclarecer,
d□ vidas 13 N□ o 28/01/2021 11 N□ o Sim N□ o N□ o
1/25/2021 23:59:36 N□ o SIM, aceito participar. SA 1963 Sim Ensino superior completo (faculdade/universidade).
Sudeste Casado(a) / Vive junto 3 Feminino Sou aposentado(a) / pensionista Sim Sim 76 158 N□ o Sim, □ s vezes. N
□ o 5 a 10 anos. N□ o, n□ o tenho nenhuma dessas doen□ as 2 est□ mago raio x, vazio durar, horas pequeno, caf□
a meia-noite, comer, nada, fazer caf□ da manh□ beba, □ gua pergunta, o departamento poder compreendo, transm
itir, importantes, diagn□ stico entendi, altera□ □ o, dez, ciente que, adaptar, direito, solicitar, telefonando precisar, i
nstru□ □ es, procurar, Servi□ o, esclarecer, d□ vidas 13 N□ o 28/01/2021 11 N□ o N□ o N□ o N□ o
1/26/2021 0:13:23 N□ o SIM, aceito participar. Ci 1977 Sim Ensino m□ dio completo (colegial). Centro-Oeste Vi□
vo(a) 1 Feminino Sou aposentado(a) / pensionista Sim Sim 103 168 N□ o Sim, □ s vezes. Sim Mais de 10 anos. Dia
betes 5 est□ mago raio x, vazio durar, horas pequeno, caf□ a meia-noite, comer, nada, fazer caf□ da manh□ beba,
□ gua pergunta, o departamento poder compreendo, transmitir, importantes, diagn□ stico entendi, altera□ □ o, dez,
ciente que, adaptar, direito, solicitar, telefonando precisar, instru□ □ es, procurar, Servi□ o, esclarecer, d□ vidas U
ma hora da tarde N□ o 28/01/2021 11 horas N□ o N□ o N□ o Sim
1/26/2021 0:59:36 N□ o SIM, aceito participar. Ce 1962 Sim Ensino m□ dio completo (colegial). Sudeste Casado(a
) / Vive junto 4 Feminino Sou aposentado(a) / pensionista Sim Sim 67 168 N□ o Sim, □ s vezes. Sim Menos de 1 an
o. N□ o, n□ o tenho nenhuma dessas doen□ as 1 est□ mago raio x, vazio durar, horas pequeno, caf□ a meia-noite,
comer, nada, fazer caf□ da manh□ beba, □ gua pergunta, o departamento poder compreendo, transmitir, important
es, diagn□ stico entendi, altera□ □ o, dez, ciente que, adaptar, direito, solicitar, telefonando precisar, instru□ □ es, p
rocurar, Servi□ o, esclarecer, d□ vidas 13 N□ o 28/01/2021 11 N□ o N□ o N□ o N□ o
1/26/2021 9:43:48 N□ o SIM, aceito participar. Ca 1958 Sim Ensino m□ dio completo (colegial). Sudeste Vi□ vo(a
) 2 Masculino Sou aposentado(a) / pensionista Sim Sim 93 175 N□ o Sim, □ s vezes. N□ o Mais de 10 anos. N□ o,
n□ o tenho nenhuma dessas doen□ as 0 est□ mago raio x, vazio durar, horas pequeno, caf□ a meia-noite, comer, na

da, fazer caf� da manh� beba, � gua pergunta, o departamento poder compreendo, transmitir, importantes, diagn
� stico entendi, altera� � o, dez, ciente que, adaptar, direito, solicitar, telefonando precisar, instru� � es, procurar,
Servi� o, esclarecer, d� vidas 13 N� o 28/01/2021 11h30 N� o N� o N� o N� o
1/26/2021 13:33:42 N� o SIM, aceito participar. Re 1966 Sim Ensino m� dio completo (colegial). Nordeste Separa
do(a) / Divorciado(a) 3 Feminino N� o Sim Sim 55 150 N� o N� o. N� o Mais de 10 anos. N� o, n� o tenho nenh
uma dessas doen� as 1 est� mago raio x, vazio durar, horas pequeno, caf� a meia-noite, comer, nada, fazer caf� d
a manh� beba, � gua pergunta, o departamento poder compreendo, transmitir, importantes, diagn� stico entendi, al
tera� � o, dez, ciente que, adaptar, direito, solicitar, telefonando precisar, instru� � es, procurar, Servi� o, esclarec
er, d� vidas 13:00hs N� o 28/01/2021 11:00hs N� o N� o N� o N� o
1/26/2021 13:49:05 N� o SIM, aceito participar. Ro 1952 Sim Ensino superior completo (faculdade/universidade).
Sul Casado(a) / Vive junto 2 Masculino Sou aposentado(a) / pensionista Sim Sim 76 156 N� o N� o. N� o Mais de
10 anos. N� o, n� o tenho nenhuma dessas doen� as 7 est� mago raio x, vazio durar, horas pequeno, caf� a meia
-noite, comer, nada, fazer caf� da manh� beba, � gua pergunta, o departamento poder compreendo, transmitir, im
portantes, diagn� stico entendi, altera� � o, dez, ciente que, adaptar, direito, solicitar, telefonando precisar, instru�
� es, procurar, Servi� o, esclarecer, d� vidas 13 Sim 28/01/2021 11 N� o N� o N� o Sim
1/26/2021 14:09:10 N� o SIM, aceito participar. Ro 1961 Sim Ensino fundamental completo. Sudeste Casado(a) / V
ive junto 2 Feminino Sim Sim Sim 78 150 N� o N� o. N� o Mais de 10 anos. N� o, n� o tenho nenhuma dessas d
oen� as 4 est� mago raio x, vazio durar, horas pequeno, caf� a meia-noite, comer, nada, fazer caf� da manh� be
ba, � gua pergunta, o departamento poder compreendo, transmitir, importantes, diagn� stico entendi, altera� � o, d
ez, ciente que, adaptar, direito, solicitar, telefonando precisar, instru� � es, procurar, Servi� o, esclarecer, d� vidas
13 N� o 28/01/2021 11 Sim N� o N� o Sim
1/26/2021 14:33:08 N� o SIM, aceito participar. Lu 1982 Sim Ensino m� dio completo (colegial). Centro-Oeste Se
parado(a) / Divorciado(a) 4 Feminino N� o Sim Sim 100 165 Sim N� o. N� o 1 a 5 anos. N� o, n� o tenho nenhu
ma dessas doen� as 5 est� mago raio x, vazio durar, horas pequeno, caf� a meia-noite, comer, nada, fazer caf� da
manh� beba, � gua pergunta, o departamento poder compreendo, transmitir, importantes, diagn� stico entendi, alt
era� � o, dez, ciente que, adaptar, direito, solicitar, telefonando precisar, instru� � es, procurar, Servi� o, esclarece
r, d� vidas As 13 horas N� o 28/01/2021 11 horas Sim Sim Sim Sim
1/26/2021 16:22:11 N� o SIM, aceito participar. Ad 1977 Sim Ensino m� dio completo (colegial). Sudeste Solteiro
(a) 5 Feminino N� o Sim Sim 170 183 N� o N� o. N� o Mais de 10 anos. Diabetes 3 est� mago raio x, vazio dura
r, horas pequeno, caf� a meia-noite, comer, nada, fazer caf� da manh� beba, � gua pergunta, o departamento pod
er compreendo, transmitir, importantes, diagn� stico entendi, altera� � o, dez, ciente que, adaptar, direito, solicitar,
telefonando precisar, instru� � es, procurar, Servi� o, esclarecer, d� vidas 13 Sim 28/01/2021 15 N� o Sim N� o
N� o
1/26/2021 18:57:49 N� o SIM, aceito participar. No 68 Sim Ensino m� dio completo (colegial). Sudeste Casado(a)
/ Vive junto 3 Feminino N� o Sim Sim 75 170 N� o N� o. Sim 5 a 10 anos. N� o, n� o tenho nenhuma dessas doe
n� as 6 est� mago raio x, vazio durar, horas pequeno, caf� a meia-noite, comer, nada, fazer caf� da manh� beba
, � gua pergunta, o departamento poder compreendo, transmitir, importantes, diagn� stico entendi, altera� � o, dez,
ciente que, adaptar, direito, solicitar, telefonando precisar, instru� � es, procurar, Servi� o, esclarecer, d� vidas Tr
eze horas N� o 29/01/2021 Onze horas Sim Sim Sim N� o
1/26/2021 21:46:03 N� o SIM, aceito participar. EU 1960 Sim Ensino superior completo (faculdade/universidade).
Nordeste Casado(a) / Vive junto 2 Feminino N� o Sim Sim 65 161 N� o N� o. Sim Mais de 10 anos. Diabetes 2 es
t� mago raio x, vazio durar, horas pequeno, caf� a meia-noite, comer, nada, fazer caf� da manh� beba, � gua pe
rgunta, o departamento poder compreendo, transmitir, importantes, diagn� stico entendi, altera� � o, dez, ciente qu
e, adaptar, direito, solicitar, telefonando precisar, instru� � es, procurar, Servi� o, esclarecer, d� vidas 13 N� o 28/
01/2021 11 N� o N� o N� o N� o
1/26/2021 22:04:09 N� o SIM, aceito participar. KA 1978 Sim P� s-Gradua� � o (especializa� � o, mestrado e/o
u doutorado). Sudeste Casado(a) / Vive junto 4 Feminino Sim Sim Sim 110 165 N� o N� o. Sim Mais de 10 anos.
N� o, n� o tenho nenhuma dessas doen� as 2 est� mago raio x, vazio durar, horas pequeno, caf� a meia-noite, co
mer, nada, fazer caf� da manh� beba, � gua pergunta, o departamento poder compreendo, transmitir, importantes,
diagn� stico entendi, altera� � o, dez, ciente que, adaptar, direito, solicitar, telefonando precisar, instru� � es, pro
curar, Servi� o, esclarecer, d� vidas 13h N� o 28/01/2021 11h N� o N� o Sim Sim
1/26/2021 22:22:58 N� o SIM, aceito participar. Ma 1965 Sim Ensino m� dio completo (colegial). Sudeste Casado(
a) / Vive junto 3 Feminino Sim Sim Sim 65 145 N� o N� o. N� o 5 a 10 anos. N� o, n� o tenho nenhuma dessas d
oen� as 5 est� mago raio x, vazio durar, horas pequeno, caf� a meia-noite, comer, nada, fazer caf� da manh� be
ba, � gua pergunta, o departamento poder compreendo, transmitir, importantes, diagn� stico entendi, altera� � o, d

ez, ciente que, adaptar, direito, solicitar, telefonando precisar, instru□ □ es, procurar, Servi□ o, esclarecer, d□ vidas
13 N□ o 28/01/2021 11:30 Sim Sim N□ o Sim
1/26/2021 22:37:43 N□ o SIM, aceito participar. El 1966 Sim Ensino m□ dio completo (colegial). Sudeste Casado(a
) / Vive junto 6 Feminino N□ o Sim Sim 80 156 Sim Sim, □ s vezes. Sim 1 a 5 anos. N□ o, n□ o tenho nenhuma de
ssas doen□ as 1 est□ mago raio x, vazio durar, horas pequeno, caf□ a meia-noite, comer, nada, fazer caf□ da manh
□ beba, □ gua pergunta, o departamento poder compreendo, transmitir, importantes, diagn□ stico entendi, altera□
□ o, dez, ciente que, adaptar, direito, solicitar, telefonando precisar, instru□ □ es, procurar, Servi□ o, esclarecer, d
□ vidas 13h N□ o 28/01/2021 11h Sim Sim N□ o N□ o
1/26/2021 22:59:46 N□ o SIM, aceito participar. Re 1970 Sim Ensino m□ dio completo (colegial). Nordeste Separa
do(a) / Divorciado(a) 3 Feminino N□ o Sim Sim 90 160 N□ o Sim, □ s vezes. Sim 1 a 5 anos. N□ o, n□ o tenho ne
nhuma dessas doen□ as 1 est□ mago raio x, vazio durar, horas pequeno, caf□ a meia-noite, comer, nada, fazer caf
□ da manh□ beba, □ gua pergunta, o departamento poder compreendo, transmitir, importantes, diagn□ stico enten
di, altera□ □ o, dez, ciente que, adaptar, direito, solicitar, telefonando precisar, instru□ □ es, procurar, Servi□ o, esc
larecer, d□ vidas 13 horas N□ o 28/01/2021 11 horas Sim Sim Sim N□ o
1/26/2021 23:27:02 N□ o SIM, aceito participar. Ja 1961 Sim Ensino m□ dio completo (colegial). Sudeste Separado
(a) / Divorciado(a) 1 Feminino Sim Sim Sim 90 165 N□ o N□ o. N□ o 1 a 5 anos. N□ o, n□ o tenho nenhuma dess
as doen□ as 3 est□ mago raio x, vazio durar, horas pequeno, caf□ a meia-noite, comer, nada, fazer caf□ da manh
□ beba, □ gua pergunta, o departamento poder compreendo, transmitir, importantes, diagn□ stico entendi, altera□
□ o, dez, ciente que, adaptar, direito, solicitar, telefonando precisar, instru□ □ es, procurar, Servi□ o, esclarecer, d
□ vidas 12 Sim 25/02/2021 10 Sim Sim Sim Sim
1/26/2021 23:52:06 N□ o SIM, aceito participar. AN 1961 Sim Ensino superior completo (faculdade/universidade).
Sul Separado(a) / Divorciado(a) 04 Feminino Sim Sim Sim 85 160 N□ o Sim, □ s vezes. N□ o 1 a 5 anos. N□ o, n
□ o tenho nenhuma dessas doen□ as 01 est□ mago raio x, vazio durar, horas pequeno, caf□ a meia-noite, comer, na
da, fazer caf□ da manh□ beba, □ gua pergunta, o departamento poder compreendo, transmitir, importantes, diagn
□ stico entendi, altera□ □ o, dez, ciente que, adaptar, direito, solicitar, telefonando precisar, instru□ □ es, procurar,
Servi□ o, esclarecer, d□ vidas 13 horas N□ o 28/01/2021 11 horas N□ o N□ o N□ o N□ o
1/27/2021 0:52:36 N□ o SIM, aceito participar. MA 1960 Sim P□ s-Gradua□ □ o (especializa□ □ o, mestrado e/ou
doutorado). Sul Separado(a) / Divorciado(a) 1 Feminino Sim Sim Sim 72 160 N□ o Sim, □ s vezes. Sim 1 a 5 anos.
N□ o, n□ o tenho nenhuma dessas doen□ as 2 est□ mago raio x, vazio durar, horas pequeno, caf□ a meia-noite, co
mer, nada, fazer caf□ da manh□ beba, □ gua pergunta, o departamento poder compreendo, transmitir, importantes,
diagn□ stico entendi, altera□ □ o, dez, ciente que, adaptar, direito, solicitar, telefonando precisar, instru□ □ es, pro
curar, Servi□ o, esclarecer, d□ vidas 13 horas Sim 28/01/2021 9 horas N□ o N□ o N□ o Sim
1/27/2021 1:10:07 N□ o SIM, aceito participar. RO 1975 Sim Ensino m□ dio completo (colegial). Sudeste Solteiro(
a) 4 Feminino N□ o Sim Sim 108 168 N□ o N□ o. N□ o 5 a 10 anos. Outra que n□ o est□ entre as op□ □ es de res
posta 3 est□ mago raio x, vazio durar, horas pequeno, caf□ a meia-noite, comer, nada, fazer caf□ da manh□ beba,
□ gua pergunta, o departamento poder compreendo, transmitir, importantes, diagn□ stico entendi, altera□ □ o, dez,
ciente que, adaptar, direito, solicitar, telefonando precisar, instru□ □ es, procurar, Servi□ o, esclarecer, d□ vidas 13
N□ o 28/01/2021 11 Sim Sim N□ o Sim
1/27/2021 1:26:54 N□ o SIM, aceito participar. Ma 1970 Sim Ensino m□ dio completo (colegial). Sul Solteiro(a) 3
Feminino Sou aposentado(a) / pensionista Sim Sim 73 155 N□ o Sim, □ s vezes. N□ o 1 a 5 anos. N□ o, n□ o tenh
o nenhuma dessas doen□ as 1 pontos raio x, vazio durar, horas pequeno, caf□ a meia-noite, comer, nada, fazer caf
□ da manh□ beba, □ gua pergunta, o departamento poder compreendo, transmitir, importantes, diagn□ stico enten
di, altera□ □ o, dez, ciente que, adaptar, direito, solicitar, telefonando precisar, instru□ □ es, procurar, Servi□ o, esc
larecer, d□ vidas 13 horas N□ o 28/01/2021 11 horas Sim Sim N□ o N□ o
1/27/2021 3:57:24 N□ o SIM, aceito participar. Si 1963 Sim Ensino m□ dio completo (colegial). Sudeste Casado(a)
/ Vive junto 4 Feminino N□ o Sim Sim 59 151 Sim Sim, □ s vezes. N□ o Menos de 1 ano. N□ o, n□ o tenho nenhu
ma dessas doen□ as 3 est□ mago raio x, vazio durar, horas pequeno, caf□ a meia-noite, comer, nada, fazer caf□ da
manh□ beba, □ gua pergunta, o departamento poder compreendo, transmitir, importantes, diagn□ stico entendi, alt
era□ □ o, dez, ciente que, adaptar, direito, solicitar, telefonando precisar, instru□ □ es, procurar, Servi□ o, esclarece
r, d□ vidas As13horas N□ o 28/01/2021 11:00horas N□ o N□ o N□ o N□ o
1/27/2021 8:30:13 N□ o SIM, aceito participar. MA 1970 Sim Ensino m□ dio completo (colegial). Sudeste Separad
o(a) / Divorciado(a) 3 Feminino Sim Sim Sim 85 150 N□ o N□ o. N□ o Mais de 10 anos. N□ o, n□ o tenho nenhu
ma dessas doen□ as 1 est□ mago raio x, vazio durar, horas pequeno, caf□ a meia-noite, comer, nada, fazer caf□ da
manh□ beba, □ gua pergunta, o departamento poder compreendo, transmitir, importantes, diagn□ stico entendi, alt
era□ □ o, dez, ciente que, adaptar, direito, solicitar, telefonando precisar, instru□ □ es, procurar, Servi□ o, esclarece

r, d� vidas 13 N� o 28/01/2021 11 N� o N� o N� o N� o
1/27/2021 9:25:46 N� o SIM, aceito participar. JU 1975 Sim P� s-Gradua� � o (especializa� � o, mestrado e/ou doutorado). Sudeste Casado(a) / Vive junto 3 Feminino Sou aposentado(a) / pensionista Sim Sim 120 165 N� o Sim , � s vezes. Sim Mais de 10 anos. Diabetes, Infarto, AVC (derrame), Outra que n� o est� entre as op� � es de res posta 7 est� mago raio x, vazio durar, horas pequeno, caf� a meia-noite, comer, nada, fazer caf� da manh� beba, � gua pergunta, o departamento poder compreendo, transmitir, importantes, diagn� stico entendi, altera� � o, dez, ciente que, adaptar, direito, solicitar, telefonando precisar, instru� � es, procurar, Servi� o, esclarecer, d� vidas As 13:00 N� o 28/01/2021 As 11:00 N� o N� o N� o Sim
1/27/2021 10:48:37 N� o SIM, aceito participar. Jo 1972 Sim P� s-Gradua� � o (especializa� � o, mestrado e/ou doutorado). Sul Casado(a) / Vive junto 3 Feminino N� o Sim Sim 97 170 N� o N� o. N� o Mais de 10 anos. Diab etes, Outra que n� o est� entre as op� � es de resposta 18 est� mago raio x, vazio durar, horas pequeno, caf� a meia-noite, comer, nada, fazer caf� da manh� beba, � gua pergunta, o departamento poder compreendo, transmiti r, importantes, diagn� stico entendi, altera� � o, dez, ciente que, adaptar, direito, solicitar, telefonando precisar, ins tru� � es, procurar, Servi� o, esclarecer, d� vidas 13h N� o 28/01/2021 11h Sim Sim N� o N� o
1/27/2021 10:53:19 N� o SIM, aceito participar. Lu 1986 Sim Ensino m� dio completo (colegial). Centro-Oeste Sol teiro(a) 4 Feminino N� o Sim Sim 49 157 N� o Sim, � s vezes. N� o Mais de 10 anos. Diabetes, Infarto, AVC (der rame), Doen� a nos rins, Outra que n� o est� entre as op� � es de resposta 8 est� mago raio x, vazio durar, horas pequeno, caf� a meia-noite, comer, nada, fazer caf� da manh� beba, � gua pergunta, o departamento poder com preendo, transmitir, importantes, diagn� stico entendi, altera� � o, dez, ciente que, adaptar, direito, solicitar, telefon ando precisar, instru� � es, procurar, Servi� o, esclarecer, d� vidas 13 horas N� o 27/01/2021 11 horas N� o N � o N� o Sim
1/27/2021 11:43:38 N� o SIM, aceito participar. Ut 1971 Sim Ensino m� dio completo (colegial). Sudeste Casado(a) / Vive junto 3 Feminino N� o Sim Sim 73 171 N� o Sim, � s vezes. N� o 1 a 5 anos. Outra que n� o est� entre as op� � es de resposta 5 est� mago raio x, vazio durar, horas pequeno, caf� a meia-noite, comer, nada, fazer caf � da manh� beba, � gua pergunta, o departamento poder compreendo, transmitir, importantes, diagn� stico enten di, altera� � o, dez, ciente que, adaptar, direito, solicitar, telefonando precisar, instru� � es, procurar, Servi� o, esc larecer, d� vidas 13 N� o 28/01/2021 11 N� o N� o N� o N� o
1/27/2021 12:27:35 N� o SIM, aceito participar. IA 1964 Sim P� s-Gradua� � o (especializa� � o, mestrado e/ou doutorado). Nordeste Casado(a) / Vive junto 2 Feminino Sou aposentado(a) / pensionista Sim Sim 70 150 N� o N� o. N� o 5 a 10 anos. N� o, n� o tenho nenhuma dessas doen� as 2 est� mago raio x, vazio durar, horas pequeno, c af� a meia-noite, comer, nada, fazer caf� da manh� beba, � gua pergunta, o departamento poder compreendo, tra nsmitir, importantes, diagn� stico entendi, altera� � o, dez, ciente que, adaptar, direito, solicitar, telefonando precis ar, instru� � es, procurar, Servi� o, esclarecer, d� vidas 13h N� o 28/01/2021 11h N� o Sim N� o Sim
1/27/2021 13:37:06 N� o SIM, aceito participar. MA 1960 Sim Ensino m� dio completo (colegial). Sudeste Casado (a) / Vive junto 3 Feminino Sou aposentado(a) / pensionista Sim Sim 78 169 N� o Sim, � s vezes. N� o 1 a 5 anos. N� o, n� o tenho nenhuma dessas doen� as 4 est� mago raio x, vazio durar, horas pequeno, caf� a meia-noite, co mer, nada, fazer caf� da manh� beba, � gua pergunta, o departamento poder compreendo, transmitir, importantes, diagn� stico entendi, altera� � o, dez, ciente que, adaptar, direito, solicitar, telefonando precisar, instru� � es, pro curar, Servi� o, esclarecer, d� vidas 13 N� o 28/01/2021 12 N� o N� o N� o N� o
1/27/2021 13:45:14 N� o SIM, aceito participar. Fa 1965 Sim Ensino fundamental completo. Nordeste Casado(a) / Vive junto 3 Feminino N� o Sim Sim 85 147 N� o N� o. Sim 5 a 10 anos. Diabetes 3 est� mago raio x, vazio dura r, horas pequeno, caf� a meia-noite, comer, nada, fazer caf� da manh� beba, � gua pergunta, o departamento pod er compreendo, transmitir, importantes, diagn� stico entendi, altera� � o, dez, ciente que, adaptar, direito, solicitar, telefonando precisar, instru� � es, procurar, Servi� o, esclarecer, d� vidas 4 vezes ao dia Sim 29/01/2021 11:00hrs Sim Sim N� o N� o
1/27/2021 14:03:43 N� o SIM, aceito participar. Ma 63 Sim Ensino fundamental completo. Sul Casado(a) / Vive ju nto 2 Feminino Sou aposentado(a) / pensionista Sim Sim 69 150 Sim Sim, � s vezes. N� o Mais de 10 anos. Outra que n� o est� entre as op� � es de resposta 4 est� mago raio x, vazio durar, horas pequeno, caf� a meia-noite, co mer, nada, fazer caf� da manh� beba, � gua pergunta, o departamento poder compreendo, transmitir, importantes, diagn� stico entendi, altera� � o, dez, ciente que, adaptar, direito, solicitar, telefonando precisar, instru� � es, pro curar, Servi� o, esclarecer, d� vidas 13.00 Sim 27/01/2021 11.30 Sim Sim Sim Sim
1/27/2021 14:27:52 N� o SIM, aceito participar. Ed 1961 Sim Ensino fundamental completo. Nordeste Solteiro(a) 2 Feminino N� o Sim Sim 71 161 N� o Sim, � s vezes. N� o 5 a 10 anos. Outra que n� o est� entre as op� � es d e resposta 5 pontos raio x, vazio durar, horas pequeno, caf� a meia-noite, comer, nada, fazer caf� da manh� beba, � gua pergunta, o departamento poder compreendo, transmitir, importantes, diagn� stico entendi, altera� � o, dez,

ciente que, adaptar, direito, solicitar, telefonando precisar, instru□ □ es, procurar, Servi□ o, esclarecer, d□ vidas 13 N□ o 28/01/2021 11 N□ o N□ o N□ o Sim
1/27/2021 17:43:07 N□ o SIM, aceito participar. MA 1965 Sim P□ s-Gradua□ □ o (especializa□ □ o, mestrado e/o u doutorado). Nordeste Solteiro(a) 1 Feminino Sim Sim Sim 70 150 N□ o Sim, □ s vezes. N□ o 1 a 5 anos. N□ o, n □ o tenho nenhuma dessas doen□ as 3 est□ mago raio x, vazio durar, horas pequeno, caf□ a meia-noite, comer, nad a, fazer caf□ da manh□ beba, □ gua pergunta, o departamento poder compreendo, transmitir, importantes, diagn□ stico entendi, altera□ □ o, dez, ciente que, adaptar, direito, solicitar, telefonando precisar, instru□ □ es, procurar, Se rvi□ o, esclarecer, d□ vidas 1 N□ o 28/01/2021 11 Sim Sim N□ o N□ o
1/27/2021 18:42:24 N□ o SIM, aceito participar. An 1970 Sim Ensino superior completo (faculdade/universidade). Nordeste Casado(a) / Vive junto 7 Feminino N□ o Sim Sim 105 171 N□ o Sim, □ s vezes. Sim 5 a 10 anos. N□ o, n □ o tenho nenhuma dessas doen□ as 3 est□ mago raio x, vazio durar, horas pequeno, caf□ a meia-noite, comer, nad a, fazer caf□ da manh□ beba, □ gua pergunta, o departamento poder compreendo, transmitir, importantes, diagn□ stico entendi, altera□ □ o, dez, ciente que, adaptar, direito, solicitar, telefonando precisar, instru□ □ es, procurar, Se rvi□ o, esclarecer, d□ vidas 13 N□ o 28/01/2021 11 Sim Sim N□ o N□ o
1/27/2021 19:06:56 N□ o SIM, aceito participar. Ma 1964 Sim Ensino m□ dio completo (colegial). Nordeste Solteir o(a) 2 Feminino Sou aposentado(a) / pensionista Sim Sim 78 160 N□ o Sim, □ s vezes. Sim 5 a 10 anos. Diabetes 4 est□ mago raio x, vazio durar, horas pequeno, caf□ a meia-noite, comer, nada, fazer caf□ da manh□ beba, □ gua pergunta, o departamento poder compreendo, transmitir, importantes, diagn□ stico entendi, altera□ □ o, dez, ciente que, adaptar, direito, solicitar, telefonando precisar, instru□ □ es, procurar, Servi□ o, esclarecer, d□ vidas As 13:00 N□ o 06/01/2021 11:00 N□ o N□ o N□ o N□ o
1/27/2021 20:11:04 N□ o SIM, aceito participar. Na 1960 Sim Ensino m□ dio completo (colegial). Nordeste Casado (a) / Vive junto 5 Feminino Sou aposentado(a) / pensionista Sim Sim 63 157 N□ o N□ o. N□ o 1 a 5 anos. Diabetes, Infarto 12 diabetes raio x, vazio durar, horas pequeno, caf□ a meia-noite, comer, nada, fazer caf□ da manh□ beba , □ gua resposta, disque poder compreendo, transmitir, importantes, diagn□ stico estabele□ o, custo, cinco, devedor que, adaptar, direito, solicitar, telefonando precisar, instru□ □ es, procurar, Servi□ o, esclarecer, d□ vidas 19 horas Sim 04/02/2021 12 horas Sim Sim N□ o Sim
1/27/2021 20:41:27 N□ o SIM, aceito participar. CC10 1957 Sim Ensino m□ dio completo (colegial). Sudeste Separ ado(a) / Divorciado(a) 4 Feminino N□ o Sim Sim 78 160 Sim Sim, □ s vezes. Sim Mais de 10 anos. N□ o, n□ o ten ho nenhuma dessas doen□ as 1 est□ mago raio x, vazio durar, horas pequeno, caf□ a meia-noite, comer, nada, fazer caf□ da manh□ beba, □ gua pergunta, o departamento poder compreendo, transmitir, importantes, diagn□ stico en tendi, altera□ □ o, dez, ciente que, adaptar, direito, solicitar, telefonando precisar, instru□ □ es, procurar, Servi□ o, esclarecer, d□ vidas As13h N□ o 27/01/2021 11h N□ o N□ o N□ o Sim
1/27/2021 21:40:10 N□ o SIM, aceito participar. RC 1967 Sim P□ s-Gradua□ □ o (especializa□ □ o, mestrado e/ou doutorado). Sudeste Casado(a) / Vive junto 2 Feminino N□ o Sim Sim 67 160 N□ o Sim, □ s vezes. N□ o 5 a 10 a nos. N□ o, n□ o tenho nenhuma dessas doen□ as 3 est□ mago raio x, vazio durar, horas pequeno, caf□ a meia-noit e, comer, nada, fazer caf□ da manh□ beba, □ gua pergunta, o departamento poder compreendo, transmitir, importa ntes, diagn□ stico entendi, altera□ □ o, dez, ciente que, adaptar, direito, solicitar, telefonando precisar, instru□ □ es, procurar, Servi□ o, esclarecer, d□ vidas 13 horas N□ o 28/01/2021 11 horas N□ o N□ o N□ o N□ o
1/27/2021 22:05:04 N□ o SIM, aceito participar. Va 1975 Sim Ensino m□ dio completo (colegial). Norte Solteiro(a) 2 Feminino Sim Sim Sim 120 163 N□ o N□ o. N□ o 5 a 10 anos. Diabetes 6 est□ mago raio x, vazio durar, horas p equeno, caf□ a meia-noite, comer, nada, fazer caf□ da manh□ beba, □ gua pergunta, o departamento poder compr eendo, transmitir, importantes, diagn□ stico entendi, altera□ □ o, dez, ciente que, adaptar, direito, solicitar, telefona ndo precisar, instru□ □ es, procurar, Servi□ o, esclarecer, d□ vidas 13 hrs Sim 28/01/2021 11 hrs Sim Sim N□ o N □ o
1/27/2021 22:25:07 N□ o SIM, aceito participar. De 1985 Sim Ensino superior completo (faculdade/universidade). Sul Separado(a) / Divorciado(a) 8 Feminino N□ o Sim Sim 76 173 Sim Sim, □ s vezes. N□ o 5 a 10 anos. N□ o, n □ o tenho nenhuma dessas doen□ as 1 est□ mago raio x, vazio durar, horas pequeno, caf□ a meia-noite, comer, nad a, fazer caf□ da manh□ beba, □ gua pergunta, o departamento poder compreendo, transmitir, importantes, diagn□ stico entendi, altera□ □ o, dez, ciente que, adaptar, direito, solicitar, telefonando precisar, instru□ □ es, procurar, Se rvi□ o, esclarecer, d□ vidas 13 N□ o 28/01/2021 11 N□ o Sim N□ o N□ o
1/27/2021 22:53:12 N□ o SIM, aceito participar. AM 1953 Sim Ensino m□ dio completo (colegial). Nordeste Separ ado(a) / Divorciado(a) 1 Feminino Sou aposentado(a) / pensionista Sim Sim 75 162 Sim N□ o. N□ o 1 a 5 anos. N □ o, n□ o tenho nenhuma dessas doen□ as 0 est□ mago raio x, vazio durar, horas caldo, lentes a meia-noite, comer, nada, fazer caf□ da manh□ beba, □ gua pergunta, o departamento poder compreendo, transmitir, importantes, diag n□ stico entendi, altera□ □ o, dez, ciente que, adaptar, direito, solicitar, telefonando precisar, instru□ □ es, procurar

, Servi□ o, esclarecer, d□ vidas 13 N□ o 28/01/2021 11 Sim N□ o N□ o N□ o

1/28/2021 1:04:23 N□ o SIM, aceito participar. Ed 1964 Sim P□ s-Gradua□ □ o (especializa□ □ o, mestrado e/ou doutorado). Nordeste Solteiro(a) 3 Feminino Sim Sim Sim 108 170 N□ o N□ o. N□ o 5 a 10 anos. Outra que n□ o e st□ entre as op□ □ es de resposta 3 est□ mago raio x, vazio durar, horas pequeno, caf□ a meia-noite, comer, nada, fazer caf□ da manh□ beba, □ gua pergunta, o departamento poder compreendo, transmitir, importantes, diagn□ sti co entendi, altera□ □ o, dez, ciente que, adaptar, direito, solicitar, telefonando precisar, instru□ □ es, procurar, Servi □ o, esclarecer, d□ vidas 13h N□ o 28/01/2021 11h N□ o N□ o N□ o N□ o

1/28/2021 3:54:16 N□ o SIM, aceito participar. De 1972 Sim Ensino m□ dio completo (colegial). Nordeste Casado(a) / Vive junto 3 Feminino Sou aposentado(a) / pensionista Sim Sim 65 155 N□ o Sim, frequentemente. Sim 1 a 5 an os. N□ o, n□ o tenho nenhuma dessas doen□ as 0 pontos raio x, vazio durar, horas pequeno, caf□ a meia-noite, co mer, nada, fazer caf□ da manh□ beba, □ gua pergunta, o departamento poder compreendo, transmitir, importantes, diagn□ stico entendi, altera□ □ o, dez, ciente isto, ocupar, esquerdo, falhar, lendo precisar, instru□ □ es, procurar, Servi□ o, esclarecer, d□ vidas 13 N□ o 28/01/2021 1 Sim Sim Sim N□ o

1/28/2021 6:37:18 N□ o SIM, aceito participar. El 1952 Sim Ensino m□ dio completo (colegial). Sudeste Vi□ vo(a) 3 Feminino Sou aposentado(a) / pensionista Sim Sim 54 148 N□ o N□ o. N□ o 1 a 5 anos. N□ o, n□ o tenho nenhu ma dessas doen□ as 0 diabetes livro, asma durar, horas pequeno, caf□ a meia-noite, comer, nada, fazer caf□ da ma nh□ dirija, cora□ □ o resposta, disque cabelo compreendo, transmitir, importantes, diagn□ stico entendi, altera□ □ o, dez, ciente que, adaptar, direito, solicitar, telefonando precisar, instru□ □ es, procurar, Servi□ o, esclarecer, d □ vidas 13 N□ o 03/02/2021 11 N□ o N□ o N□ o N□ o

1/28/2021 7:00:22 N□ o SIM, aceito participar. Ji 1969 Sim Ensino superior completo (faculdade/universidade). Su deste Separado(a) / Divorciado(a) 2 Feminino Sim Sim Sim 83 154 N□ o N□ o. N□ o 5 a 10 anos. N□ o, n□ o tenh o nenhuma dessas doen□ as 1 est□ mago raio x, vazio durar, horas pequeno, caf□ a meia-noite, comer, nada, fazer caf□ da manh□ beba, □ gua pergunta, o departamento poder compreendo, transmitir, importantes, diagn□ stico ent endi, altera□ □ o, dez, ciente que, adaptar, direito, solicitar, telefonando precisar, instru□ □ es, procurar, Servi□ o, e sclarecer, d□ vidas 13 Sim 28/01/2021 11 Sim Sim Sim Sim

1/28/2021 9:25:54 N□ o SIM, aceito participar. Re 1957 Sim Ensino m□ dio completo (colegial). Sul Separado(a) / Divorciado(a) 4 Feminino N□ o Sim Sim 77 166 Sim N□ o. N□ o Mais de 10 anos. N□ o, n□ o tenho nenhuma des sas doen□ as 3 est□ mago raio x, vazio durar, horas pequeno, caf□ a meia-noite, comer, nada, fazer caf□ da manh □ beba, □ gua pergunta, o departamento poder compreendo, transmitir, importantes, diagn□ stico entendi, altera□ □ o, dez, ciente que, adaptar, direito, solicitar, telefonando precisar, instru□ □ es, procurar, Servi□ o, esclarecer, d □ vidas 13h N□ o 28/01/2021 11h N□ o N□ o N□ o N□ o

1/28/2021 12:20:27 N□ o SIM, aceito participar. D□ 1968 Sim Ensino superior completo (faculdade/universidade). Sudeste Separado(a) / Divorciado(a) 2 Feminino N□ o Sim Sim 57 158 Sim N□ o. N□ o 1 a 5 anos. AVC (derrame) 2 est□ mago raio x, vazio durar, horas pequeno, caf□ a meia-noite, comer, nada, fazer caf□ da manh□ beba, □ g ua pergunta, o departamento poder compreendo, transmitir, importantes, diagn□ stico entendi, altera□ □ o, dez, cien te que, adaptar, direito, solicitar, telefonando precisar, instru□ □ es, procurar, Servi□ o, esclarecer, d□ vidas 13 N□ o 04/03/2021 11 N□ o N□ o N□ o N□ o

1/28/2021 13:20:03 N□ o SIM, aceito participar. An 1989 Sim Ensino m□ dio completo (colegial). Sul Separado(a) / Divorciado(a) 2 Feminino Sim Sim Sim 67 154 N□ o N□ o. Sim Mais de 10 anos. N□ o, n□ o tenho nenhuma des sas doen□ as 3 est□ mago raio x, vazio durar, horas pequeno, caf□ a meia-noite, comer, nada, fazer caf□ da manh □ beba, □ gua pergunta, o departamento poder compreendo, transmitir, importantes, diagn□ stico entendi, altera□ □ o, dez, ciente que, adaptar, direito, solicitar, telefonando precisar, instru□ □ es, procurar, Servi□ o, esclarecer, d □ vidas 13 horas N□ o 04/02/2021 11 horas N□ o N□ o N□ o N□ o

1/28/2021 14:52:29 N□ o SIM, aceito participar. LR 1972 Sim P□ s-Gradua□ □ o (especializa□ □ o, mestrado e/ou doutorado). Sul Separado(a) / Divorciado(a) 2 Feminino Sim Sim Sim 126 175 N□ o Sim, □ s vezes. Sim 1 a 5 ano s. N□ o, n□ o tenho nenhuma dessas doen□ as 0 est□ mago raio x, vazio durar, horas pequeno, caf□ a meia-noite, comer, nada, fazer caf□ da manh□ beba, □ gua pergunta, o departamento poder compreendo, transmitir, important es, diagn□ stico entendi, altera□ □ o, dez, ciente que, adaptar, direito, solicitar, telefonando precisar, instru□ □ es, p rocurar, Servi□ o, esclarecer, d□ vidas 13 Sim 04/02/2021 11 Sim Sim N□ o Sim

1/28/2021 16:45:44 N□ o SIM, aceito participar. Id 1978 Sim Ensino superior completo (faculdade/universidade). S udeste Solteiro(a) 1 Feminino Sim Sim Sim 100 170 N□ o Sim, □ s vezes. N□ o 5 a 10 anos. Diabetes 5 est□ mago raio x, vazio durar, horas pequeno, caf□ a meia-noite, comer, nada, fazer caf□ da manh□ beba, □ gua pergunta, o departamento poder compreendo, transmitir, importantes, diagn□ stico entendi, altera□ □ o, dez, ciente que, adaptar , direito, solicitar, telefonando precisar, instru□ □ es, procurar, Servi□ o, esclarecer, d□ vidas 13 N□ o 04/02/2021 11 Sim Sim N□ o N□ o

1/28/2021 17:05:34 N� o SIM, aceito participar. Ve 1945 Sim Ensino superior completo (faculdade/universidade). Sudeste Separado(a) / Divorciado(a) 1 Feminino Sou aposentado(a) / pensionista Sim Sim 72 153 N� o Sim, � s vezes. N� o Mais de 10 anos. N� o, n� o tenho nenhuma dessas doen� as 6 est� mago raio x, vazio durar, horas pequeno, caf� a meia-noite, comer, nada, fazer caf� da manh� beba, � gua pergunta, o departamento poder compreendo, transmitir, importantes, diagn� stico entendi, altera� � o, dez, ciente que, adaptar, direito, solicitar, telefonando precisar, instru� � es, procurar, Servi� o, esclarecer, d� vidas 13 N� o 04/02/2021 11 N� o N� o N� o N� o

1/28/2021 17:45:39 N� o SIM, aceito participar. Lu 1963 Sim Ensino superior completo (faculdade/universidade). Sudeste Casado(a) / Vive junto 3 Feminino N� o Sim Sim 79 164 N� o Sim, � s vezes. N� o 5 a 10 anos. N� o, n� o tenho nenhuma dessas doen� as 2 est� mago raio x, vazio durar, horas pequeno, caf� a meia-noite, comer, nada, fazer caf� da manh� beba, � gua pergunta, o departamento poder compreendo, transmitir, importantes, diagn� stico entendi, altera� � o, dez, ciente que, adaptar, direito, solicitar, telefonando precisar, instru� � es, procurar, Servi� o, esclarecer, d� vidas 13 N� o 04/02/2021 11 N� o N� o N� o N� o
1/28/2021 17:47:33 N� o SIM, aceito participar. SP 1969 Sim Ensino fundamental completo. Sul Casado(a) / Vive junto 2 Feminino N� o Sim Sim 70 154 N� o Sim, � s vezes. Sim Mais de 10 anos. N� o, n� o tenho nenhuma dessas doen� as 3 est� mago raio x, vazio durar, horas pequeno, caf� a meia-noite, comer, nada, fazer caf� da manh� beba, � gua pergunta, o departamento poder compreendo, transmitir, importantes, diagn� stico entendi, altera� � o, dez, ciente que, adaptar, direito, solicitar, telefonando precisar, instru� � es, procurar, Servi� o, esclarecer, d� vidas 14hrs N� o 04/02/2021 11hrs N� o N� o N� o N� o
1/28/2021 18:16:29 N� o SIM, aceito participar. An 70 Sim Ensino fundamental completo. Sudeste Solteiro(a) 2 Feminino Sim Sim Sim 107 166 N� o Sim, � s vezes. N� o Mais de 10 anos. N� o, n� o tenho nenhuma dessas doen� as 2 est� mago raio x, vazio durar, horas pequeno, caf� a meia-noite, comer, nada, fazer caf� da manh� beba, � gua pergunta, o departamento poder compreendo, transmitir, importantes, diagn� stico entendi, altera� � o, dez, ciente que, adaptar, direito, solicitar, telefonando precisar, instru� � es, procurar, Servi� o, esclarecer, d� vidas 13 N� o 04/02/2021 11 Sim Sim N� o N� o
1/28/2021 18:47:08 N� o SIM, aceito participar. GI 1974 Sim P� s-Gradua� � o (especializa� � o, mestrado e/ou doutorado). Sudeste Casado(a) / Vive junto 3 Feminino Sim Sim Sim 99 163 N� o N� o. N� o 1 a 5 anos. Outra que n� o est� entre as op� � es de resposta 1 est� mago raio x, vazio durar, horas pequeno, caf� a meia-noite, comer, nada, fazer caf� da manh� beba, � gua pergunta, o departamento poder compreendo, transmitir, importantes, diagn� stico entendi, altera� � o, dez, ciente que, adaptar, direito, solicitar, telefonando precisar, instru� � es, procurar, Servi� o, esclarecer, d� vidas 13 N� o 04/02/2021 11 N� o N� o N� o N� o
1/28/2021 19:00:05 N� o SIM, aceito participar. Re 1973 Sim Ensino m� dio completo (colegial). Sudeste Casado(a) / Vive junto 4 Feminino N� o Sim Sim 60 158 Sim N� o. Sim 5 a 10 anos. Doen� a nos rins, Outra que n� o est� entre as op� � es de resposta 0 est� mago raio x, vazio durar, horas pequeno, caf� a meia-noite, comer, nada, fazer caf� da manh� beba, � gua pergunta, o departamento poder compreendo, transmitir, importantes, diagn� stico entendi, altera� � o, dez, ciente que, adaptar, direito, solicitar, telefonando precisar, instru� � es, procurar, Servi� o, esclarecer, d� vidas 14 horas Sim 28/01/2021 11 horas Sim Sim Sim Sim
1/28/2021 19:03:14 N� o SIM, aceito participar. Li 1980 Sim Ensino superior completo (faculdade/universidade). Nordeste Solteiro(a) 2 Feminino Sim Sim Sim 105 170 N� o N� o. N� o 1 a 5 anos. Diabetes 7 est� mago raio x, vazio durar, horas pequeno, caf� a meia-noite, comer, nada, fazer caf� da manh� beba, � gua pergunta, o departamento poder compreendo, transmitir, importantes, diagn� stico entendi, altera� � o, dez, ciente que, adaptar, direito, solicitar, telefonando precisar, instru� � es, procurar, Servi� o, esclarecer, d� vidas 13 N� o 28/01/2021 11 Sim Sim N� o N� o
1/28/2021 19:40:14 N� o SIM, aceito participar. SO 1954 Sim Ensino superior completo (faculdade/universidade). Sudeste Solteiro(a) 1 Feminino Sim Sim Sim 72 163 N� o Sim, � s vezes. N� o Mais de 10 anos. N� o, n� o tenho nenhuma dessas doen� as 3 est� mago raio x, vazio durar, horas pequeno, caf� a meia-noite, comer, nada, fazer caf� da manh� beba, � gua pergunta, o departamento poder compreendo, transmitir, importantes, diagn� stico entendi, altera� � o, dez, ciente que, adaptar, direito, solicitar, telefonando precisar, instru� � es, procurar, Servi� o, esclarecer, d� vidas 13 horas N� o 26/02/2021 1100 hs N� o Sim N� o Sim
1/28/2021 20:40:29 N� o SIM, aceito participar. AL 1977 Sim P� s-Gradua� � o (especializa� � o, mestrado e/ou doutorado). Norte Solteiro(a) 4 Feminino Sim Sim Sim 113 170 N� o N� o. N� o 5 a 10 anos. Diabetes 2 est� mago raio x, vazio durar, horas pequeno, caf� a meia-noite, comer, nada, fazer caf� da manh� beba, � gua pergunta , o departamento poder compreendo, transmitir, importantes, diagn� stico entendi, altera� � o, dez, ciente que, adaptar, direito, solicitar, telefonando precisar, instru� � es, procurar, Servi� o, esclarecer, d� vidas 13h N� o 04/02/2021 11h Sim Sim Sim N� o

1/28/2021 21:15:40 N□ o SIM, aceito participar. Ju 1977 Sim Ensino m□ dio completo (colegial). Sudeste Solteiro(
a) 3 Feminino Sim Sim Sim 85 168 N□ o Sim, □ s vezes. N□ o Mais de 10 anos. Outra que n□ o est□ entre as op
□ □ es de resposta 4 est□ mago raio x, vazio durar, horas pequeno, caf□ a meia-noite, comer, nada, fazer caf□ da
manh□ beba, □ gua pergunta, o departamento poder compreendo, transmitir, importantes, diagn□ stico entendi, alte
ra□ □ o, dez, ciente que, adaptar, direito, solicitar, telefonando precisar, instru□ □ es, procurar, Servi□ o, esclarecer
, d□ vidas 13 horas N□ o 04/02/2021 11 horas N□ o Sim N□ o Sim
1/28/2021 21:47:57 N□ o SIM, aceito participar. Sa 1965 Sim Ensino m□ dio completo (colegial). Sudeste Casado(
a) / Vive junto 5 Feminino N□ o Sim Sim 70 152 Sim N□ o. N□ o Mais de 10 anos. Outra que n□ o est□ entre as o
p□ □ es de resposta 3 est□ mago raio x, vazio durar, horas pequeno, caf□ a meia-noite, comer, nada, fazer caf□ da
manh□ beba, □ gua pergunta, o departamento poder compreendo, transmitir, importantes, diagn□ stico entendi, alt
era□ □ o, dez, ciente que, adaptar, direito, solicitar, telefonando precisar, instru□ □ es, procurar, Servi□ o, esclarece
r, d□ vidas 13 N□ o 28/01/2021 11 N□ o N□ o N□ o N□ o
1/28/2021 22:27:27 N□ o SIM, aceito participar. Si 19/04/1972 Sim Ensino m□ dio completo (colegial). Sudeste Se
parado(a) / Divorciado(a) 3 Feminino Sim Sim Sim 82 170 N□ o Sim, □ s vezes. N□ o 5 a 10 anos. N□ o, n□ o ten
ho nenhuma dessas doen□ as 2 est□ mago raio x, vazio durar, horas pequeno, caf□ a meia-noite, comer, nada, fazer
caf□ da manh□ beba, □ gua pergunta, o departamento poder compreendo, transmitir, importantes, diagn□ stico en
tendi, altera□ □ o, dez, ciente que, adaptar, direito, solicitar, telefonando precisar, instru□ □ es, procurar, Servi□ o,
esclarecer, d□ vidas As 13 hs N□ o 28/02/2021 11 horas Sim Sim Sim Sim
1/28/2021 23:26:28 N□ o SIM, aceito participar. Ro 1972 Sim Ensino superior completo (faculdade/universidade).
Sul Separado(a) / Divorciado(a) 2 Feminino N□ o Sim Sim 68 156 N□ o Sim, □ s vezes. N□ o Mais de 10 anos. Ou
tra que n□ o est□ entre as op□ □ es de resposta 0 est□ mago raio x, vazio durar, horas pequeno, caf□ a meia-noite
, comer, nada, fazer caf□ da manh□ beba, □ gua pergunta, o departamento poder compreendo, transmitir, importan
tes, diagn□ stico entendi, altera□ □ o, dez, ciente que, adaptar, direito, solicitar, telefonando precisar, instru□ □ es,
procurar, Servi□ o, esclarecer, d□ vidas 13 N□ o 04/02/2021 11 Sim Sim Sim Sim
1/28/2021 23:32:40 N□ o SIM, aceito participar. Ed 79 Sim Ensino m□ dio completo (colegial). Sudeste Casado(a)
/ Vive junto 4 Feminino N□ o Sim Sim 83 158 N□ o N□ o. N□ o 5 a 10 anos. Diabetes 8 est□ mago raio x, vazio d
urar, horas pequeno, caf□ a meia-noite, comer, nada, fazer caf□ da manh□ beba, □ gua pergunta, o departamento
poder compreendo, transmitir, importantes, diagn□ stico entendi, altera□ □ o, dez, ciente que, adaptar, direito, solici
tar, telefonando precisar, instru□ □ es, procurar, Servi□ o, esclarecer, d□ vidas 13 horas N□ o 28/01/2021 11horas
Sim Sim N□ o Sim
1/28/2021 23:46:13 N□ o SIM, aceito participar. Li 1971 Sim Ensino m□ dio completo (colegial). Nordeste Casado(
a) / Vive junto 3 Feminino N□ o Sim Sim 78 158 N□ o N□ o. N□ o Mais de 10 anos. N□ o, n□ o tenho nenhuma d
essas doen□ as 3 est□ mago raio x, vazio durar, horas pequeno, caf□ a meia-noite, comer, nada, fazer caf□ da man
h□ beba, □ gua pergunta, o departamento poder compreendo, transmitir, importantes, diagn□ stico entendi, altera□
□ o, dez, ciente que, adaptar, direito, solicitar, telefonando precisar, instru□ □ es, procurar, Servi□ o, esclarecer, d
□ vidas 13 horas N□ o 28/01/2021 11 Sim Sim Sim Sim
1/29/2021 0:12:58 N□ o SIM, aceito participar. Is 1997 Sim Ensino superior completo (faculdade/universidade). Ce
ntro-Oeste Solteiro(a) 7 Feminino N□ o Sim Sim 70 152 N□ o Sim, □ s vezes. N□ o 1 a 5 anos. N□ o, n□ o tenho
nenhuma dessas doen□ as 5 est□ mago raio x, vazio durar, horas pequeno, caf□ a meia-noite, comer, nada, fazer ca
f□ da manh□ beba, □ gua pergunta, o departamento poder compreendo, transmitir, importantes, diagn□ stico ente
ndi, altera□ □ o, dez, ciente que, adaptar, direito, solicitar, telefonando precisar, instru□ □ es, procurar, Servi□ o, es
clarecer, d□ vidas 14:00 N□ o 28/01/2021 11 N□ o Sim Sim Sim
1/29/2021 0:45:55 N□ o SIM, aceito participar. Ce 1962 Sim Ensino m□ dio completo (colegial). Sudeste Separado(
a) / Divorciado(a) 2 Feminino Sim Sim Sim 84 150 N□ o N□ o. Sim Menos de 1 ano. N□ o, n□ o tenho nenhuma d
essas doen□ as 0 est□ mago raio x, vazio durar, horas pequeno, caf□ a meia-noite, comer, nada, fazer caf□ da man
h□ beba, □ gua pergunta, o departamento poder compreendo, transmitir, importantes, diagn□ stico entendi, altera□
□ o, dez, ciente que, adaptar, direito, solicitar, telefonando precisar, instru□ □ es, procurar, Servi□ o, esclarecer, d
□ vidas 13 horas Sim 29/01/2021 11 horas Sim N□ o N□ o Sim
1/29/2021 7:24:10 N□ o SIM, aceito participar. An 1965 Sim Ensino m□ dio completo (colegial). Nordeste Casado(
a) / Vive junto 05 Feminino N□ o Sim Sim 66 158 N□ o Sim, □ s vezes. N□ o Mais de 10 anos. Outra que n□ o est
□ entre as op□ □ es de resposta 4 est□ mago raio x, vazio durar, horas pequeno, caf□ a meia-noite, comer, nada, f
azer caf□ da manh□ beba, □ gua resposta, disque poder compreendo, transmitir, importantes, diagn□ stico estabel
e□ o, custo, cinco, devedor assim, alimentar, brilho, reciclar, contando precisar, instru□ □ es, procurar, Servi□ o, es
clarecer, d□ vidas 13 N□ o 11/02/2021 9 Sim Sim Sim Sim
1/29/2021 7:55:38 N□ o SIM, aceito participar. Cl 1972 Sim Ensino m□ dio completo (colegial). Sudeste Casado(a)

/ Vive junto 5 Feminino N□ o Sim Sim 68 165 N□ o N□ o. N□ o Menos de 1 ano. Diabetes, Outra que n□ o est□
entre as op□ □ es de resposta 5 est□ mago raio x, vazio durar, horas pequeno, caf□ a meia-noite, comer, nada, faze
r caf□ da manh□ beba, □ gua pergunta, o departamento poder compreendo, transmitir, importantes, diagn□ stico e
ntendi, altera□ □ o, dez, ciente que, adaptar, direito, solicitar, telefonando precisar, instru□ □ es, procurar, Servi□ o
, esclarecer, d□ vidas 13 N□ o 04/02/2021 11 Sim Sim Sim Sim

1/29/2021 8:25:25 N□ o SIM, aceito participar. Ju 1976 Sim Ensino m□ dio completo (colegial). Sudeste Casado(a)
/ Vive junto 3 Feminino Sim Sim Sim 139 178 N□ o Sim, □ s vezes. N□ o 1 a 5 anos. N□ o, n□ o tenho nenhuma
dessas doen□ as 3 est□ mago raio x, vazio durar, horas pequeno, caf□ a meia-noite, comer, nada, fazer caf□ da ma
nh□ beba, □ gua pergunta, o departamento poder compreendo, transmitir, importantes, diagn□ stico entendi, altera
□ □ o, dez, ciente que, adaptar, direito, solicitar, telefonando precisar, instru□ □ es, procurar, Servi□ o, esclarecer,
d□ vidas 13:00 N□ o 03/02/2021 11:00 Sim Sim N□ o N□ o

1/29/2021 9:41:17 N□ o SIM, aceito participar. LE 1977 Sim Ensino m□ dio completo (colegial). Sudeste Solteiro(a
) 1 Masculino N□ o Sim Sim 96 171 Sim Sim, frequentemente. N□ o Mais de 10 anos. N□ o, n□ o tenho nenhuma
dessas doen□ as 2 est□ mago raio x, vazio durar, horas pequeno, caf□ a meia-noite, comer, nada, fazer caf□ da ma
nh□ beba, □ gua pergunta, o departamento poder compreendo, transmitir, importantes, diagn□ stico entendi, altera
□ □ o, dez, ciente que, adaptar, direito, solicitar, telefonando precisar, instru□ □ es, procurar, Servi□ o, esclarecer,
d□ vidas 13 N□ o 04/02/2021 11 Sim Sim N□ o Sim

1/29/2021 11:26:32 N□ o SIM, aceito participar. Kr 1984 Sim Ensino fundamental completo. Centro-Oeste Casado(
a) / Vive junto 4 Feminino N□ o Sim Sim 80 155 N□ o Sim, □ s vezes. N□ o Menos de 1 ano. N□ o, n□ o tenho ne
nhuma dessas doen□ as 0 pontos raio x, vazio durar, horas pequeno, caf□ a meia-noite, comer, nada, fazer caf□ da
manh□ beba, □ gua resposta, disque poder compreendo, transmitir, importantes, diagn□ stico entendi, altera□ □ o,
dez, ciente que, adaptar, direito, solicitar, telefonando precisar, instru□ □ es, procurar, Servi□ o, esclarecer, d□ vida
s 7 Sim 29/01/2021 Duas ou tr□ s hiras ap□ s refei□ □ o Sim Sim N□ o N□ o
